# Selectivity of Rh⋅⋅⋅H−C Binding in a σ‐Alkane Complex Controlled by the Secondary Microenvironment in the Solid State

**DOI:** 10.1002/chem.202004585

**Published:** 2021-01-12

**Authors:** Samantha K. Furfari, Bengt E. Tegner, Arron L. Burnage, Laurence R. Doyle, Alexander J. Bukvic, Stuart A. Macgregor, Andrew S. Weller

**Affiliations:** ^1^ Department of Chemistry University of York York YO10 5DD UK; ^2^ Institute of Chemical Sciences Heriot-Watt University Edinburgh EH14 4AS UK; ^3^ Department of Chemistry University of Oxford Mansfield Road Oxford OX1 3TA UK

**Keywords:** density functional calculations, isomerization, periodic DFT, rhodium, selectivity, SMOM

## Abstract

Single‐crystal to single‐crystal solid‐state molecular organometallic (SMOM) techniques are used for the synthesis and structural characterization of the σ‐alkane complex [Rh(*t*Bu_2_PCH_2_CH_2_CH_2_P*t*Bu_2_)(η^2^,η^2^‐C_7_H_12_)][BAr^F^_4_] (Ar^F^=3,5‐(CF_3_)_2_C_6_H_3_), in which the alkane (norbornane) binds through two *exo*‐C−H⋅⋅⋅Rh interactions. In contrast, the bis‐cyclohexyl phosphine analogue shows *endo*‐alkane binding. A comparison of the two systems, supported by periodic DFT calculations, NCI plots and Hirshfeld surface analyses, traces this different regioselectivity to subtle changes in the local microenvironment surrounding the alkane ligand. A tertiary periodic structure supporting a secondary microenvironment that controls binding at the metal site has parallels with enzymes. The new σ‐alkane complex is also a catalyst for solid/gas 1‐butene isomerization, and catalyst resting states are identified for this.

## Graphical abstract

**Importance of microenvironment**: By using solid‐state molecular organometallic (SMOM) techniques, the σ‐alkane complex [Rh(*t*Bu_2_PCH_2_CH_2_CH_2_P*t*Bu_2_)(η^2^,η^2^‐C_7_H_12_)][BAr^F^_4_], in which the alkane binds through two *exo*‐C−H⋅⋅⋅Rh interactions, is synthesized and structurally characterized. This is different from the analogous complex with PCy_2_ groups. Subtle differences in the microenvironment, as encoded by the diene precursor complex, are shown to determine the selectivity of alkane binding.

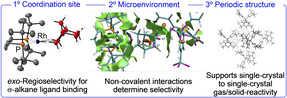

## Introduction

**Figure 1:**
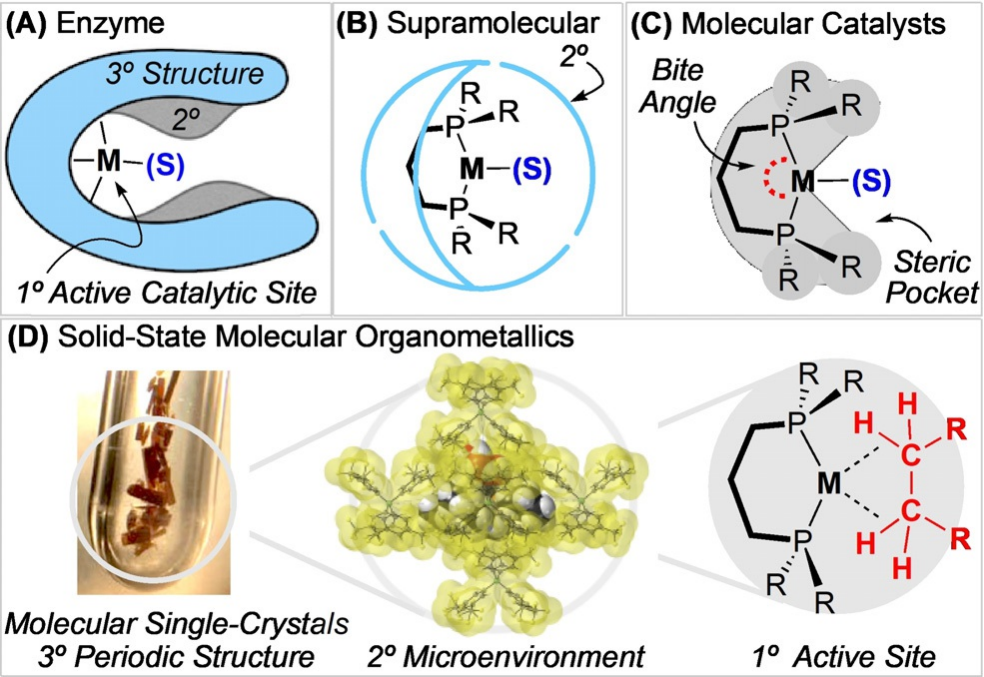
Catalyst systems: A) enzyme, B) supramolecular, C) molecular. D) Solid‐state molecular organometallic (SMOM) chemistry, highlighting the 3° periodic crystalline structure, 2° microenvironment, and 1° metal/ligand site.

**Scheme 1:**
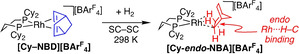
Synthesis of **[Cy‐*endo*‐NBA][BAr^F^**_**4**_ **]** using SMOM techniques.

Metalloenzyme catalysis is often characterized by high selectivity. This is a consequence of confinement effects at the primary catalytic site, the secondary microenvironment that controls substrate binding, and a tertiary structure that promotes substrate selectivity and mobility (Figure 1 A).^[1]^ For example, in P450 enzymes, modification of such chemical space results in a change in selectivity for alkane oxidation.[^2^, ^3^] Inspired by enzymatic processes, supramolecular catalysts exploit the influence of the secondary coordination sphere, allowing the stability, reactivity, and selectivity of transition metal catalysts to be modified by the installed microenvironment (Figure 1 B).[^4^, ^5^, ^6^, ^7^] In contrast, the optimization of catalytic processes promoted by molecular transition metal complexes in solution is primarily limited to varying only the primary coordination sphere, for example, by changing ligand bite‐angle and steric profile (Figure 1 C).^[8]^ We have recently exploited the stabilizing secondary microenvironment using solid‐state molecular organometallic (SMOM) chemistry and single‐crystal to single‐crystal (SC‐SC) solid/gas reactivity[^9^, ^10^] for the synthesis, structural characterization, and study of the reactivity of cationic group 9 alkane σ‐complexes.[^11^, ^12^, ^13^] For example, [Rh(dcpp)(*endo*‐η^2^,η^2^‐C_7_H_12_)][BAr^F^_4_], **[Cy‐*endo*‐NBA][BAr^F^**_**4**_ **]** (dcpp=Cy_2_PCH_2_CH_2_CH_2_PCy_2_, NBA=norbornane, Ar^F^=3,5‐(CF_3_)_2_C_6_H_3_, Scheme 1), is formed by the addition of H_2_ to single crystals of a norbornadiene (NBD) precursor.^[14]^ In solution, σ‐alkane complexes can only be observed transiently, even at very low temperatures.[^15^, ^16^] In contrast, **[Cy‐*endo*‐NBA][BAr^F^**_**4**_ **]** is stable at room temperature as single crystals, although it decomposes immediately in solution at 183 K.

Such remarkable reactivity and stability in the solid state results from the periodic arrangement of [BAr^F^_4_]^−^ anions. These form an encapsulating, approximately octahedral (*O*_h_) microenvironment around the cationic σ‐complex (Figure 1 D),[^11^, ^14^] which provides stabilizing noncovalent interactions and substrate‐accessible hydrophobic channels.^[17]^ We now report that the *regioselectivity* of alkane binding can be controlled by subtle changes to this periodic microenvironment, which are encoded by the cationic precursor metal–ligand fragment. This parallels selectivity in enzymes that is promoted using primary and secondary coordination sphere control, as supported by the tertiary structure.

## Results and Discussion

### Synthesis and structural analysis of the σ‐alkane complex

**Figure 2:**
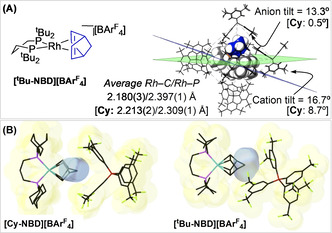
A) **[*t*Bu‐NBD][BAr^F^**_**4**_ **]** and the *O*_h_ anion motif showing the tilt of cation (van der Waals radii) and anion. Metrics for **[Cy‐NBD][BAr^F^**_**4**_ **]** in square brackets. B) Comparison of proximal cation/anion pair. Pocket volumes defined by removal of the NBD ligand: **[*t*Bu‐NBD][BAr^F^**_**4**_ **]** (62 Å^3^), **[Cy‐NBD][BAr^F^**_**4**_ **]** (65 Å^3^).^[19]^

The new precursor complex [Rh(dtbpp)(NBD)][BAr^F^_4_], **[*t*Bu‐NBD][BAr^F^**_**4**_ **]** (dtbpp=*t*Bu_2_PCH_2_CH_2_CH_2_P*t*Bu_2_) was prepared as a red crystalline solid. The solid‐state structure (from single‐crystal X‐ray diffraction) shows an approximate *O*_h_ cage of [BAr^F^_4_]^−^ anions (Figure S6, Supporting Information). Although this motif of anions is similar to that observed for **[Cy‐NBD][BAr^F^**_**4**_ **]** (Figure 2 A), close inspection of the environment around the cation shows that the metal fragment is orientated differently (Figure S34, Supporting Information). In **[Cy‐NBD][BAr^F^**_**4**_ **]**, the cation sits rather symmetrically in this ≈*O*_h_ cage with the NBD ligand centered between two aryl rings (Figure 2 B), as we have commented upon previously.^[13]^ In **[*t*Bu‐NBD][BAr^F^**_**4**_ **]**, the cation and anion‐aryl groups are slightly tilted (Figure 2 A), but in opposite directions, which combine to direct the NBD methylene bridge toward a single anion‐aryl face. The drivers for this change may come from differences in ligand peripheries^[18]^ and the more compact Rh−C_NBD_ distances in **[*t*Bu‐NBD][BAr^F^**_**4**_ **]**. Whereas the pocket volume defined by removal of the NBD does not change significantly between the Cy and *t*Bu variants, the shape does (Figure 2 B). These structural differences carry over into the resulting σ‐alkane complex, **[*t*Bu‐*exo*‐NBA][BAr^F^**_**4**_ **]**.

**Figure 3:**
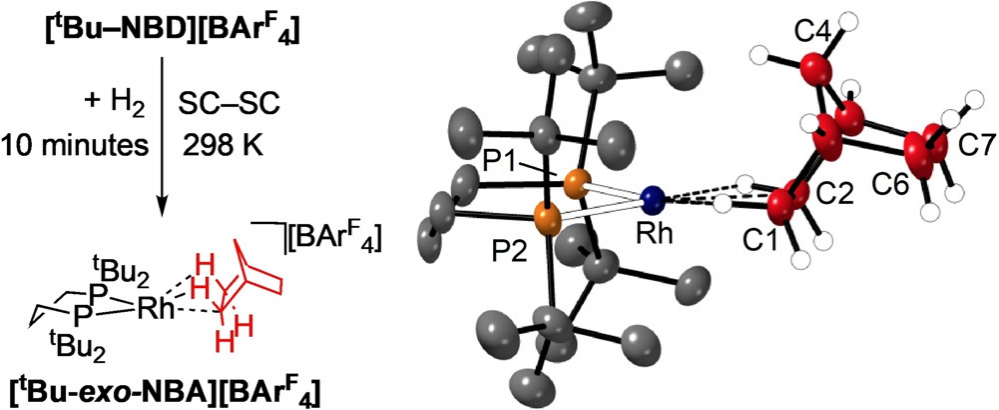
Synthesis and solid‐state structure of **[*t*Bu‐*exo*‐NBA][BAr^F^**_**4**_ **]**. 30 % displacement ellipsoids. Only the cation is shown. Selected distances (Å) and angles (°): C1−C2, 1.53(1); C6−C7, 1.53(1), Rh⋅⋅⋅C1, 2.363(7); Rh⋅⋅⋅C2, 2.382(7); Rh−P1, 2.244(2); Rh−P2, 2.235(2), ∠P1P2Rh/RhC1C2=2.0°. Selected hydrogen atoms shown (calculated positions).

Addition of H_2_ (1 atm, 10 min, optimized) to orange single crystals of **[*t*Bu‐NBD][BAr^F^**_**4**_ **]** resulted in an SC‐SC transformation and the formation of dark red **[*t*Bu‐*exo*‐NBA][BAr^F^**_**4**_ **]**. A crystal was transferred rapidly to a precooled diffractometer at 150 K for analysis. Figure 3 shows the solid‐state structure of the cation (*R=*7.5 %). Longer reaction times result in loss of crystallinity and the formation of hydride species,^[20]^ which we have not characterized further. The bond lengths in the hydrocarbon ligand indicate that C−C single bonds have formed upon addition of H_2_, with the NBA binding through two η^2^,η^2^ Rh⋅⋅⋅H−C^[14]^ 3c–2e interactions at the Rh^I^ center. The metrical data are very similar to those of **[Cy‐*endo*‐NBA][BAr^F^**_**4**_ **]**,^[14]^ despite the slightly increased P−Rh−P bite angle [96.39(7)° versus 93.91(2)°] and decreased buried volume [%*V*_bur_=60.4 versus 57.9,^[21]^ Figure S36, Supporting Information] in the *t*Bu congener. The similarities in the Rh⋅⋅⋅H−C interactions were confirmed by Quantum Theory of Atoms in Molecules (QTAIM) and Natural Bond Orbital (NBO) studies on the **[*t*Bu‐*exo*‐NBA]^+^** and **[Cy‐*endo*‐NBA]^+^** cations (Figures S44 and S47, Supporting Information).

**Figure 4:**
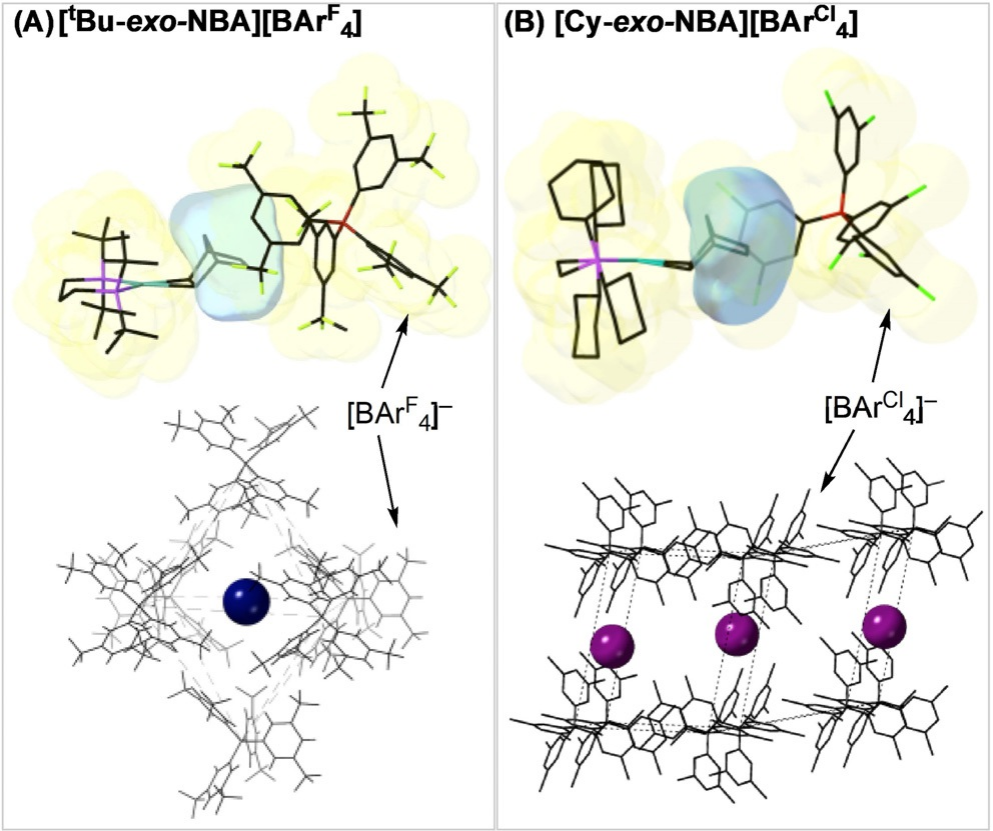
Comparison of the local environment and motif of anions that surround the cations for A) **[*t*Bu‐*exo*‐NBA][BAr^F^**_**4**_ **]** and B) **[Cy‐*exo*‐NBA][BAr^Cl^**_**4**_ **]**. Cation location indicated by spheres of arbitrary radius.

The major difference between the *t*Bu and Cy variants is the regioselectivity of alkane binding: *exo*‐C−H binding for *t*Bu and *endo* for Cy. As both retain an ≈*O*_h_ motif for the arrangement of the [BAr^F^_4_]^−^ anions around the cation in the lattice, it falls to more subtle differences in the microenvironment, as encoded in the tertiary periodic structure of the NBD precursors, to influence the regioselectivity of alkane binding. Figure 4 A shows the cation, proximal anion, and cage motif for **[*t*Bu‐*exo*‐NBA][BAr^F^**_**4**_ **]**, which highlight the similarity with the NBD precursor (i.e., Figure 2), in particular the orientation of the NBA ligand, with an ethylene bridge (C6/C7) directed toward a *single* aryl face.

**Figure 5:**
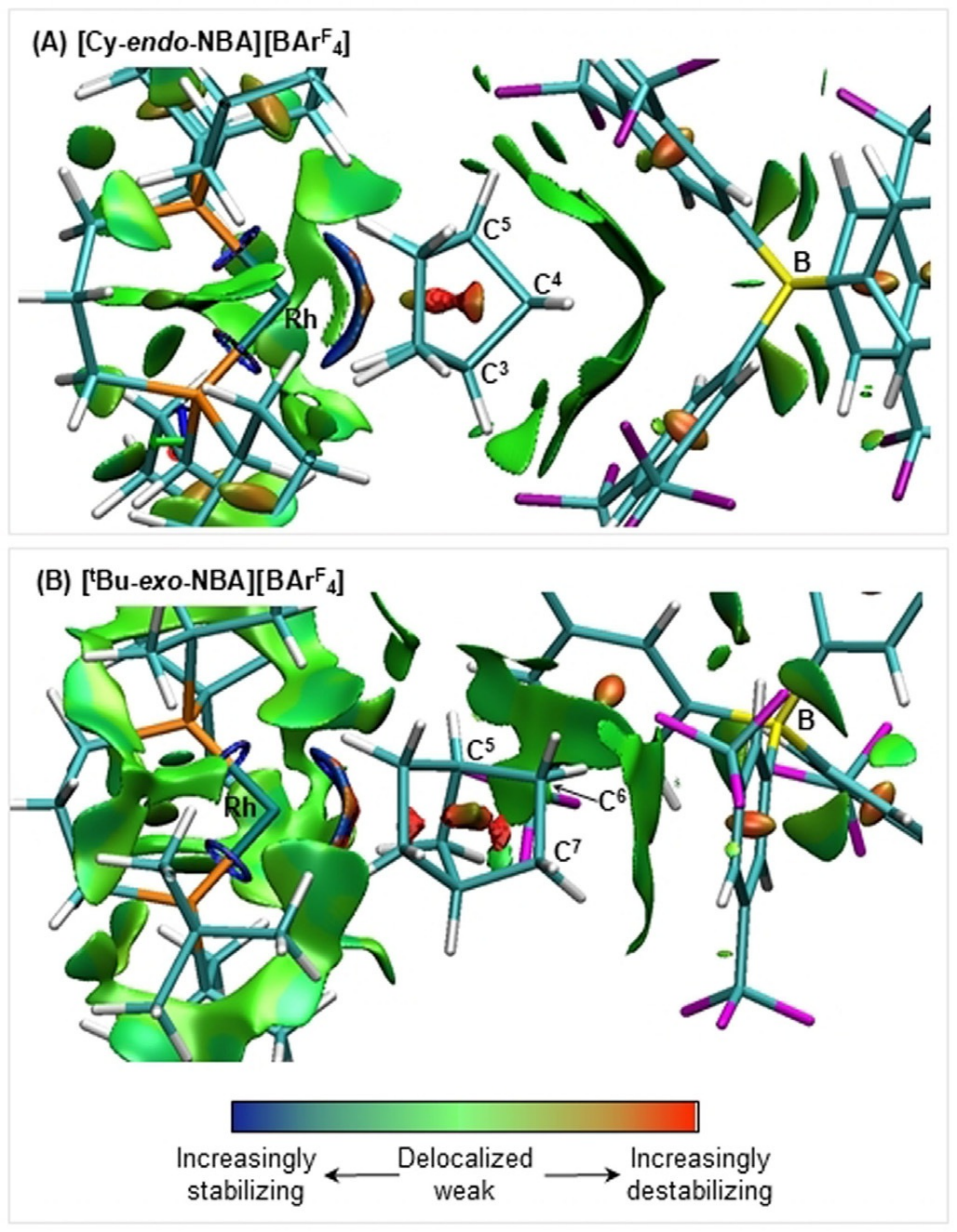
NCI plots of A) the **[Cy‐*endo*‐NBA][BAr^F^**_**4**_ **]** ion pair and B) the **[*t*Bu‐*exo*‐NBA][BAr^F^**_**4**_ **]** ion pair; isosurfaces generated for *σ*=0.3 a.u. and *ρ* between −0.07 and 0.07 a.u.

The differences between the interactions of the NBA ligands in **[*t*Bu‐*exo*‐NBA][BAr^F^**_**4**_ **]** and **[Cy‐*endo*‐NBA][BAr^F^**_**4**_ **]** with the local microenvironment are further highlighted in the noncovalent interaction (NCI) plots calculated for the proximal ion pairs (Figure 5). In both cases, broad areas of green, weakly stabilizing, dispersive interactions are seen between the NBA and two aryl groups of the neighboring [BAr^F^_4_]^−^ anion. This feature is rather symmetric for **[Cy‐*endo*‐NBA][BAr^F^**_**4**_ **]**, and involves the C^3^H−C^4^H_2_−C^5^H bridge of the NBA ligand (Figure 5 A). In contrast, the *exo*‐NBA ligand in **[*t*Bu‐*exo*‐NBA][BAr^F^**_**4**_ **]** interacts primarily along C^7^H_2_−C^6^H_2_−C^5^H (Figure 5 B). In both cases, more localized disc‐like features reflect the presence of stabilizing nonclassical C−H^δ+^⋅⋅⋅F^δ−^−C hydrogen bonds. Underscoring the importance of this local anion environment, the *exo*‐regioselectivity for NBA binding is also observed in [Rh(dcpe)‐*exo*‐(NBA)][BAr^Cl^_4_], **[Cy‐*exo*‐NBA][BAr^Cl^**_**4**_ **]** (Ar^Cl^=3,5‐Cl_2_‐C_6_H_3_).^[22]^ Despite the [BAr^Cl^_4_]^−^ adopting a very different periodic arrangement of anions (Figure 4 B), the microenvironment around the NBA ligand is broadly similar to that of **[*t*Bu‐*exo*‐NBA][BAr^F^**_**4**_ **]**. Tilting of the anion results in a rotation of one of the aryl groups, and the corresponding NCI plot for **[Cy‐*exo*‐NBA][BAr^Cl^**_**4**_ **]** reflects this, with a broad stabilizing feature attributed to the interaction of the NBA with the face of one aryl substituent (as seen for **[*t*Bu‐*exo*‐NBA][BAr^F^**_**4**_ **]**), and more localized features attributed to atom⋅⋅⋅atom contacts with the rotated aryl group (Figure S49, Supporting Information).

**Figure 6:**
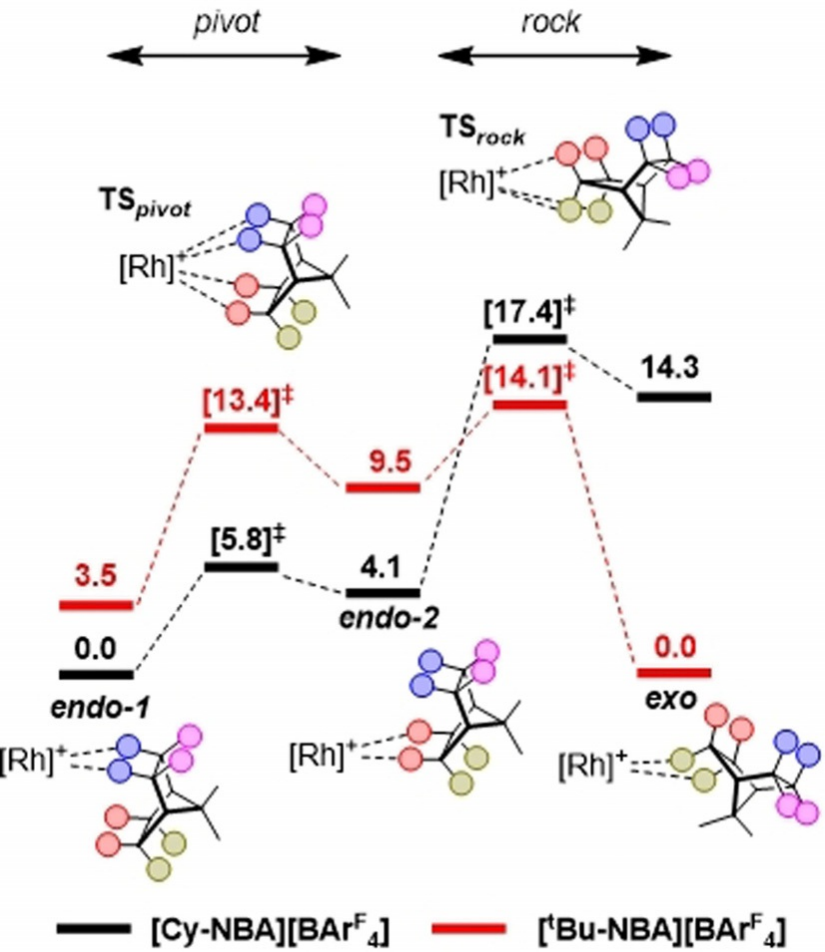
Free energy profiles (kcal mol^−1^) computed in the solid state for *exo*↔*endo* rearrangement in **[*t*Bu‐NBA][BAr^F^**_**4**_ **]** (red) and **[Cy‐NBA][BAr^F^**_**4**_ **]** (black).

To quantify the impact of the microenvironment and primary coordination sphere (i.e., the chelating ligand) on the regioselectivity of alkane coordination, we performed periodic‐DFT calculations on both the observed **[*t*Bu‐*exo*‐NBA][BAr^F^**_**4**_ **]** and **[Cy‐*endo*‐NBA][BAr^F^**_**4**_ **]** structures, and the complementary (non‐observed) **[*t*Bu‐*endo*‐NBA][BAr^F^**_**4**_ **]** and **[Cy‐*exo*‐NBA][BAr^F^**_**4**_ **]**. The latter structures were generated by a rock/pivot motion^[23]^ of one NBA ligand within the unit cell to generate the alternative NBA binding mode. Figure 6 shows the computed free energy profiles for this process. In each case, the crystallographically observed structure is computed to be more stable: by 3.5 kcal mol^−1^ for **[*t*Bu‐*exo*‐NBA][BAr^F^**_**4**_ **]** and by 14.3 kcal mol^−1^ for **[Cy‐*endo*‐NBA][BAr^F^**_**4**_ **]**. The barrier for interconversion is also higher for **[Cy‐*endo*‐NBA][BAr^F^**_**4**_ **]** (+17.4 kcal mol^−1^ cf. +14.1 kcal mol^−1^ for **[*t*Bu‐*exo*‐NBA][BAr^F^**_**4**_ **]**), and in both systems **TS**_***rock***_ is the higher transition state along the rearrangement profile.

In addition to these periodic‐DFT calculations, molecular calculations were also run on the isolated **[*t*Bu‐NBA]^+^** and **[Cy‐NBA]^+^** cations. In this case, *both* systems showed a preference for the *exo* isomer, by 1.0 and 1.5 kcal mol^−1^, respectively. Therefore, in the absence of the solid‐state microenvironment, there is a small intrinsic preference for the *exo* binding mode, irrespective of the nature of the chelating ligand.^[24]^ This preference is further enhanced in the solid state for **[*t*Bu‐*exo*‐NBA][BAr^F^**_**4**_ **]** but is strongly disfavored in **[Cy‐*endo*‐NBA][BAr^F^**_**4**_ **]**, underlining the defining role that the microenvironment can have in the selectivity of alkane binding.

**Figure 7:**
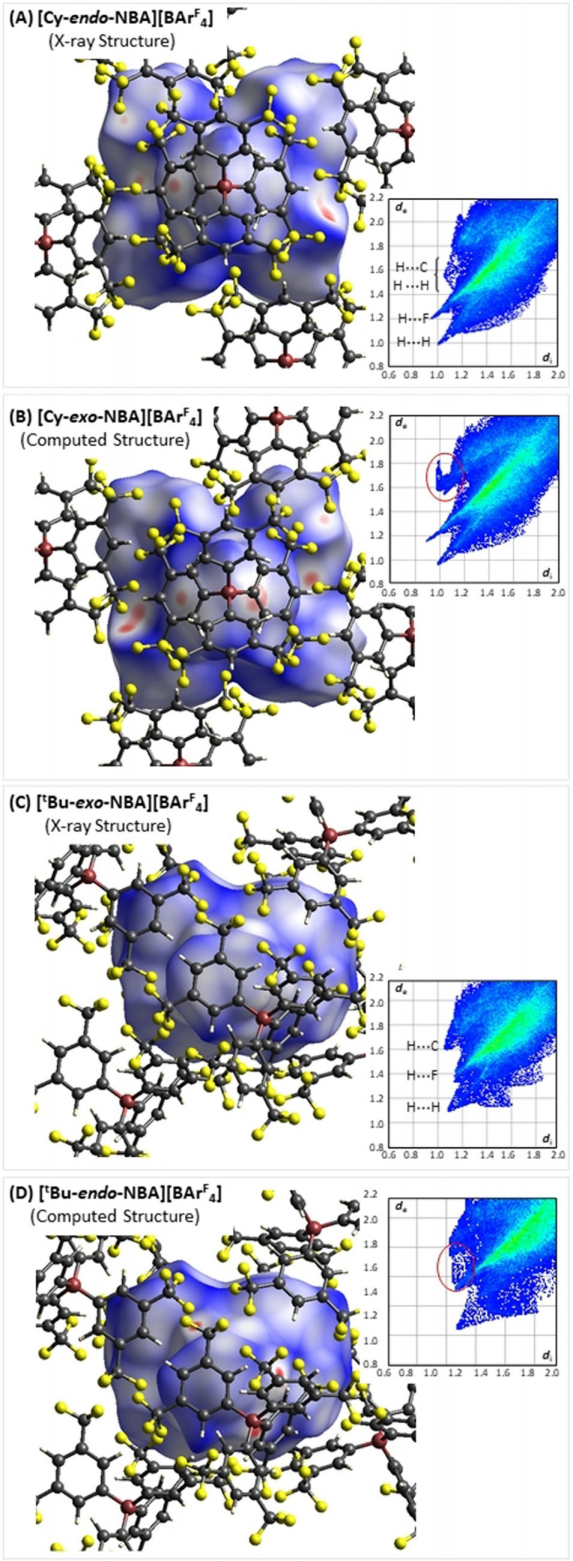
Hirshfeld surfaces mapped over *d*_norm_ and associated fingerprint plots (Å) for **[Cy‐NBA][BAr^F^**_**4**_ **]** (A,B) and **[*t*BuNBA][BAr^F^**_**4**_ **]** (C,D). For **[Cy‐NBA][BAr^F^**_**4**_ **]** the surface is viewed down the Rh⋅⋅⋅B vector of the proximal ion pair (cf. Figure 5), and this view is offset slightly for **[*t*Bu‐NBA][BAr^F^**_**4**_ **]**.

The selectivity of alkane binding was further probed using Hirshfeld surfaces and fingerprint plots generated with Crystal Explorer (Figure 7).[^25^, ^26^, ^27^] The Hirshfeld surface identifies short contacts between atoms on the central probe entity (here the different [(R_2_P(CH_2_)_3_PR_2_)Rh(NBA)]^+^ cations) and the surrounding environment (here the neighboring octahedron of [BAr^F^_4_]^−^ anions): red identifies short contacts (less than the sum of the van der Waals radii); white indicates contacts that are close to the sum of the van der Waals radii; and blue depicts those contacts that are longer than the sum of the van der Waals radii. Note that a short contact may be either stabilizing or destabilizing, depending on the pair of atoms involved. For example, stabilizing nonclassical C−H^δ+^⋅⋅⋅F^δ−^−C hydrogen bonds and destabilizing short H⋅⋅⋅H or H⋅⋅⋅C contacts will all appear as red features. In such instances the fingerprint plot allows identification of the atoms involved in a particular short contact.

The fingerprint plot for **[Cy‐*endo*‐NBA][BAr^F^**_**4**_ **]** (Figure 7 A) is typical of these systems with the feature terminating around (1.0, 1.0) corresponding to H⋅⋅⋅H contacts, that at (0.95, 1.2) attributed to H⋅⋅⋅F contacts, and the broad feature around (1.1, 1.55) a combination of short H⋅⋅⋅C and longer H⋅⋅⋅H contacts (see Supporting Information, Figures S51–S56 for separate atom⋅⋅⋅atom plots). In this case, the red short contacts seen in the Hirshfeld surface correspond to stabilizing C−H^δ+^⋅⋅⋅F^δ−^−C hydrogen bonds. The Hirshfeld surface for **[Cy‐*exo*‐NBA][BAr^F^**_**4**_ **]** (Figure 7 B) exhibits many more red short contacts, and the fingerprint plot suggests some shorter H⋅⋅⋅F contacts are present. However, the major change is the broad new feature (circled in Figure 7 B), which corresponds to destabilizing C−H_NBA_⋅⋅⋅C_aryl_ short contacts. This is reflected in the presence of the broad red features in the Hirshfeld surface, situated below the aryl groups of a [BAr^F^_4_]^−^ anion. These significant differences between the *endo*‐ and *exo*‐bound forms are consistent with the large computed energy difference of 14.3 kcal mol^−1^ between the two (Figure 6). In contrast, the distinctions between the analyses of the observed structure of **[*t*Bu‐*exo*‐NBA][BAr^F^**_**4**_ **]** and the computed **[*t*Bu‐*endo*‐NBA][BAr^F^**_**4**_ **]** structure are more subtle (Figure 7 C,D), as might be expected given the smaller energy difference between the two (3.5 kcal mol^−1^). The latter indicates some shorter H⋅⋅⋅F contacts but also more destabilizing H⋅⋅⋅C_aryl_ contacts, as evidenced by the filling in of the “bay” between the H⋅⋅⋅F and H⋅⋅⋅C “peninsulas”, as highlighted in Figure 7 D for **[*t*Bu‐*endo*‐NBA][BAr^F^**_**4**_ **]**.

### Characterization by solid‐state NMR and phase changes

**Figure 8:**
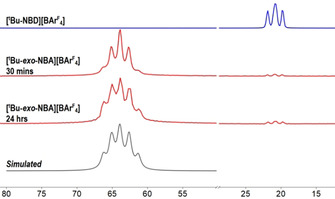
^31^P{^1^H} SSNMR spectra (162 MHz, spinning rate=10 kHz) of the evolution of **[*t*Bu‐*exo*‐NBA][BAr^F^**_**4**_ **]** with time and comparison with **[*t*Bu‐NBD][BAr^F^**_**4**_ **]**.

Turning to the bulk characterization of the new SMOM systems, **[*t*Bu‐NBD][BAr^F^**_**4**_ **]** and **[*t*Bu‐*exo*‐NBA][BAr^F^**_**4**_ **]** were characterized by SSNMR spectroscopy using samples (≈50 mg) of finely crushed crystalline materials. The resulting data are fully consistent with the structures determined by X‐ray diffraction. For **[*t*Bu‐NBD][BAr^F^**_**4**_ **]**, the ^31^P{^1^H} SSNMR spectrum shows two closely spaced signals at approximately *δ*=21 [*J*(RhP) ≈168 Hz], whereas NBD signals are observed between *δ*=79 and 51 in the ^13^C{^1^H} SSNMR spectrum (Figures S4 and S5, Supporting Information). On addition of H_2_ to form **[*t*Bu‐*exo*‐NBA][BAr^F^**_**4**_ **]** (10 min^[28]^) and transfer to the spectrometer, two environments are still observed in the ^31^P{^1^H} SSNMR spectrum, but they are downfield shifted [*δ*≈63.8] and show increased coupling constants [*J*(RhP)≈200 Hz], Figure 8. The ^13^C{^1^H} SSNMR spectrum is silent in the alkene region (110–40 ppm, Figure S12, Supporting Information). These spectroscopic data signal σ‐alkane complex formation.[^12^, ^13^, ^14^]

**[*t*Bu‐*exo*‐NBA][BAr^F^**_**4**_ **]** evolves with time in the solid state at 298 K. After 24 h, new signals have developed at slightly higher and lower chemical shifts. There is no further change after seven days at 298 K. Simulating this data (Figure 8 and Figures S10 and S11, Supporting Information) allows these new signals to be modelled as the outer lines of two doublets [*δ*=62.0, 65.8 ppm; *J*(RhP) ≈210 Hz], in 40 % relative proportion to the initial species. This ratio does not change in the 158 K ^31^P{^1^H} SSNMR spectrum, indicating that a dynamic equilibrium with a relatively low barrier is not operational. We see no evidence for coordination of the [BAr^F^_4_]^−^ counterion^[13]^ or dehydrogenation^[12]^ of NBA to form norbornene (solution trapping experiments only recover NBA). Although the relatively sharp SSNMR spectrum suggests the local order is retained around the cation,^[29]^ inspection of the X‐ray diffraction pattern reveals a phase change has occurred so that it is now strongly modulated, showing multiple satellites around the main Bragg reflections (Figure S14, Supporting Information).^[30]^ We have not been able to model this successfully, and therefore, it is not clear whether it is caused by anion and/or cation reorientation. However, spectroscopic data, trapping experiments, and catalysis (vide infra) suggest that it is a structural rather than a chemical change, and a σ‐alkane complex is retained. Periodic to incommensurately modulated phase changes in single crystals have been reported previously,^[31]^ and we have noted both modulated structures^[18]^ and phase changes^[20]^ in SMOM systems. As **[Cy‐*endo*‐NBA][BAr^F^**_**4**_ **]** is not reported to undergo a phase change,^[14]^ we suggest that the subtle changes in the microenvironment result in a *meta*stable system for **[*t*Bu‐*exo*‐NBA][BAr^F^**_**4**_ **]**. This may be related to the more kinetically and thermodynamically accessible alkane ligand reorganization in the latter (e.g., Figure 6), but as the precise structure of the modulated phase is currently not known we are reluctant to comment further.

### Addition of 1‐butene: Solid/gas butene isomerization

**Scheme 2:**
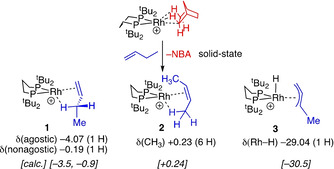
Reaction of **[*t*Bu‐*exo*‐NBA][BAr^F^**_**4**_ **]** with 1‐butene and key NMR data. Selected data given [calculated chemical shifts].

Addition of 1‐butene to either immediately prepared, or aged under Ar, samples of **[*t*Bu‐*exo*‐NBA][BAr^F^**_**4**_ **]** (Scheme 2) resulted in significant loss of diffraction quality, so a reliable structural solution was not possible, although Bragg diffraction was still observed.^[32]^ ^31^P{^1^H} SSNMR spectroscopy showed the complete consumption of the σ‐alkane complex and the formation of three products (Figure S26, Supporting Information).^[33]^ Vacuum transfer of CD_2_Cl_2_ onto the crystalline material and analysis at 183 K using solution ^31^P{^1^H}, ^1^H{^1^P}, ^1^H/^31^P HSQC/TOSCY NMR experiments, guided by calculated chemical shifts from DFT, showed these to be the butene complexes, [Rh(dtbpp)(L)][BAr^F^_4_] [L=1‐butene, **1**;^[34]^ *cis*‐2‐butene, **2** ^[35]^], and the Rh^III^ allyl hydride [RhH(dtbpp)(η^3^‐C_4_H_7_)][BAr^F^_4_], **3**, observed in a 1:1.6:1.4 ratio, respectively. This ratio changes in relative proportion with warming to 208 K, showing that the complexes are in equilibrium. DFT calculations on the molecular cations in CH_2_Cl_2_ solvent reveal that these three species all lie within 0.8 kcal mol^−1^ of one another, consistent with the equilibrium mixture observed (Figure S50, Supporting Information). All three complexes have inequivalent ^31^P environments that show coupling with ^103^Rh. Diagnostic signals in the high‐field region of the ^1^H NMR spectrum are attributed to agostic Rh⋅⋅⋅H−C interactions, and a Rh−H group [*δ*=−29.04 ppm, *J*(RhH)=32 Hz] in the allyl hydride, **3**. Whereas similar butene complexes to **1** and **2** are formed from solid/gas reactions with Rh^I^ σ‐alkane complexes having R_2_P(CH_2_)_2_PR_2_ ligands (R=Cy,^[36]^ *t*Bu^[20]^), tautomeric Rh^III^ allyl hydrides that come from C−H oxidative cleavage have only been identified indirectly by mechanistic and DFT studies as higher‐energy intermediates in alkene double‐bond isomerization processes for these^[36]^ and related MOF systems.^[37]^ Here, we suggest the larger P−Rh−P ligand bite‐angle makes the Rh^III^ oxidation state more accessible,^[38]^ and this key intermediate can now be observed. Warming this mixture to room temperature resulted in decomposition to a variety of unidentified complexes. This, again,^[36]^ demonstrates the utility of the SMOM technique in stabilizing reactive species not accessible using solution methods.

**Figure 9:**
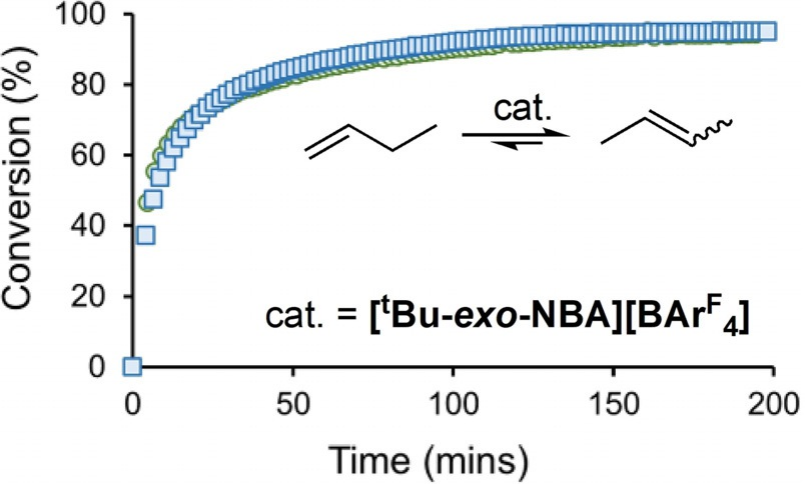
Batch isomerization of 1‐butenes using **[*t*Bu‐*exo*‐NBA][BAr^F^**_**4**_ **]** as a catalyst (298 K, 2 mg catalyst, 1 bar butene, [Rh]_TOTAL_/butenes=1:58]. ○=Immediately prepared catalyst; □=aged under Ar for 24 h.

The observation of **1**, **2**, and **3** suggests that crystalline **[*t*Bu‐*exo*‐NBA][BAr^F^**_**4**_ **]** would be a solid/gas butene isomerization catalyst. This is the case, and using finely crushed material under batch conditions at 298 K, a thermodynamic mixture of 1‐butene (5 %) and 2‐butenes (95 %) is established (see Figure 9). Freshly prepared and modulated materials show the same temporal profile. However, catalysis is rather slow (TOF_90 %_=35 h^−1^) compared with [Rh(dtbpe)(NBA)][BAr^F^_4_] (80 h^−1^)^[20]^ and [Rh(dcpe)(NBA)][BAr^F^_4_] (3000 h^−1^),^[36]^ despite having the same ≈*O*_h_ arrangement of anions, and compared with single‐crystalline Rh‐MOF systems (2000 h^−1^).^[9]^ The precursor complex **[*t*Bu‐NBD][BAr^F^**_**4**_ **]** is inactive, demonstrating the requirement for a relatively weakly bound alkane ligand for catalysis in the solid state.

## Conclusions

We show here that subtle differences in the microenvironment that supports σ‐alkane complex formation in SMOM systems controls the regioselectivity of alkane bonding, whereas changes at the primary active site result in different catalytic activities and observed resting states for butene isomerization. What is initially surprising, but perhaps more obvious with hindsight, is that these microenvironment effects seem to dominate for the cyclohexyl system, switching the intrinsic regioselectivity for *exo*‐NBA binding seen in the isolated cation, whereas for tert‐butyl the microenvironment is such that regioselectivity is unaffected. Whether this increased influence leads to the remarkable relative stabilities observed for a wide range of σ‐alkane complexes of the cyclohexyl system[^11^, ^12^, ^13^, ^14^, ^36^] remains to be determined. These observations not only reinforce the analogy between single‐crystalline SMOM systems and metalloenzymes, but also suggest that the Cy‐functionalized phosphine SMOM systems are the current best candidates for exploring chemical space with regard to new σ‐alkane complexes. The influence of the microenvironment in controlling both *stability* and *reactivity* in σ‐alkane complexes is thus of particular interest, especially with regard to simple but challenging transformations such as acceptorless alkane dehydrogenation.^[12]^

## Conflict of interest

The authors declare no conflict of interest.

## Supporting information

As a service to our authors and readers, this journal provides supporting information supplied by the authors. Such materials are peer reviewed and may be re‐organized for online delivery, but are not copy‐edited or typeset. Technical support issues arising from supporting information (other than missing files) should be addressed to the authors.

Supplementary
