# Supplementary material for: Selectivity of Rh⋅⋅⋅H−C Binding in a σ‐Alkane Complex Controlled by the Secondary Microenvironment in the Solid State
Source: Chemistry. 2021 Jan 12;27(9):3177–83. doi: 10.1002/chem.202004585 (PMC7898853; doi:10.1002/chem.202004585)
Supplement: Supplementary file 1 — Supplementary [file CHEM-27-3177-s001.pdf]

Chemistry–A European Journal

Supporting Information

Selectivity of Rh···H–C Binding in a σ-Alkane Complex Controlled by the Secondary Microenvironment in the Solid State

Samantha K. Furfari,[a] Bengt E. Tegner,[b] Arron L. Burnage,[b] Laurence R. Doyle,[a] Alexander J. Bukvic,[a, c] Stuart A. Macgregor,*[b] and Andrew S. Weller*[a]

Contents

S1 General Experimental Procedures 3
S2 Characterisation 3
S2.1 Solution NMR spectroscopic data 3
S2.2 Solid State NMR spectroscopic data 3
S2.3 X-Ray Diffraction 4
S2.4 Mass Spectrometry and Microanalysis 4
S3 Ligand and Complex Synthesis 4
S3.1 Synthesis of $[Rh(nbd)_2][BAr^F_4]$ 4
S3.2 Synthesis of $P^tBu_2CH_2CH_2CH_2P^tBu_2$ 4
S3.3 Synthesis and Characterisation of $[^tBu\text{-NBD}][BAr^F_4]$ 5
S3.4 Synthesis and Characterisation of [^{t}Bu-*exo*-NBA][BAr^F_4] 8
S3.5 Volatile Trapping Experiments 9
S4 1-Butene Isomerisation 13
S4.1 Batch Conditions 13
S4.2 1-Butene Addition to [^{t}Bu-*exo*-NBA][BAr^F_4] Experiments 15
S4.3 Synthesis of $[^tBu\text{-}C_4H_6][BAr^F_4]$ 21
S5 Anion Microenvironment Comparisons 24
S6 Steric Topography 24
S7 Volume Cavity Calculations 25
S8 Computational Methods 27
S8.1 Solid State Calculations 27
S8.2 Molecular Calculations 28
S8.3 Electronic Structure Analyses 28
S8.4 Solution-phase NMR Calculations on the Isomers of $[Rh(dtbpp)(C_4H_8)]^+$ 28
S9 Computed Reaction Profiles 29
S9.1 NBA rearrangement processes in the solid state 29
S9.2 Different NBA rearrangements in the solid state 29
S10 Electronic Structure Analyses 30
S10.1 [Cy-*endo*-NBA][BAr^F_4] 30
S10.2 [^{t}Bu-*exo*-NBA][BAr^F_4] 33
S11 Isomers of $[Rh(dtbpp)(C_4H_8)]^+$ 37
S12 Crystal Explorer 38
S12.1 [Cy-*endo*/*exo*-NBA][BAr^F_4] 38
S12.2 [^{t}Bu-*exo*/*endo*-NBA][BAr^F_4] 42
S13 Computed Energies for Molecular Species 46

S14 References 47

S1 General Experimental Procedures

All synthetic manipulations were performed using standard Schlenk and glove box techniques under an atmosphere of argon, unless otherwise specified. All glassware was oven dried (140 °C) followed by drying under vacuum prior to use. All solvents required for air sensitive reactions were dried, degassed and stored for use under argon in Rotaflo® or J. Young stopcock ampoules. Degassing solvents and reagents was achieved through three freeze-pump-thaw cycles. Dichloromethane and pentane were collected from an MBraun SPS-800 solvent purification system, degassed and stored over activated 3Å molecular sieves. Tetrahydrofuran used for ligand synthesis was stirred over sodium/benzophenone until a persistent purple colour was obtained then vacuum distilled and degassed. 2,5-Norbornadiene (bicyclo[2.2.1]hepta-2,5-diene, NBD) was stirred for 24 hours over sodium pieces, vacuum distilled and degassed. Acetonitrile-d_3 (CD_3CN), Dichloromethane-d_2 (CD_2Cl_2) and benzene-d_6 (C_6D_6) were purchased from Cambridge Isotope Laboratories. CD_3CN was vacuum distilled from phosphorus pentoxide, degassed and stored over activated 3Å molecular sieves. CD_2Cl_2 was dried overnight with calcium hydride, vacuum distilled, degassed and stored over activated 3Å molecular sieves. C_6D_6 was vacuum distilled from sodium pieces and degassed. High purity hydrogen gas (≥99.9%) was employed in the gas/solid transformations. ^nBuLi was purchased from Sigma-Aldrich and decanted into a J. Young stopcock ampoule and titrated prior to use using the method outlined by Hoye.[1] All other reagents were purchased from either Precious Metals Online, CK Gases or Sigma-Aldrich and used as received.

The metal precursors $[Rh(\mu\text{-}Cl)(nbd)]_2$[2] and $Na[BAr^F_4]$[3] were synthesised according to literature procedures: The complex $[Rh(nbd)_2][BAr^F_4]$ and ligand bis(1,3-di-tert-butylphosphino)propane were synthesised by a modification to published procedures and are included below.[4]

S2 Characterisation

S2.1 Solution NMR spectroscopic data

Solution State NMR data were collected on either a Bruker Avance III HD 400 nanobay spectrometer or Bruker Avance II 500 Cryoprobe spectrometer at the temperatures specified. The ^1H solution spectra are referenced to residual non-deuterated solvent peak and $^{13}C\{^1H\}$ solution spectra referenced to the deuterated solvent peak.[5] $^{31}P\{^1H\}$ solution spectra were externally referenced to 85% H_3PO_4 in D_2O. $^{19}F\{^1H\}$ solution spectra were externally referenced to 1% $CFCl_3$ in $CHCl_3$ and $^{11}B\{^1H\}$ solution spectra were externally referenced to 5% $BF_3{\cdot}OEt_2$ in C_6D_6. Non-deuterated solvents and gas-phase samples were locked to external neat dichloromethane-d_2 samples. All chemical shifts (δ) are reported in parts per million (ppm) and multiplicities are denoted as singlet (s), doublet (d), triplet (t), quartet (q) or multiplet (m) and prefixed broad (br) where applicable. Coupling constants are reported in hertz (Hz). In general, the solution ^{19}F and ^{11}B NMR resonances are not reported since they consist of a single resonance at δ_F = -62.8 and δ_B = -6.6 that correspond to the $[BAr^F_4]^-$ anion.

S2.2 Solid State NMR spectroscopic data

Solid-state NMR samples were prepared in an argon-filled glovebox by pre-loading *ca.* 65 mg of the crushed crystalline sample into a 4.0 mm zirconia solid-state rotor. Solid-state NMR spectra were obtained on a Bruker Avance III HD spectrometer, operating at 100.56 MHz (^{13}C) or 161.89 MHz (^{31}P), with a MAS rate (v) of 10 kHz. For $^{13}C\{^1H\}$ CP/MAS SSNMR a sequence with a variable X-amplitude spin-lock pulse[6] and spinal64 proton decoupling was used. Relaxation time for ^1H and contact time for $^{31}P\{^1H\}$ CP/MAS and $^{13}C\{^1H\}$ CP/MAS NMR experiments were optimised for each compound. All $^{13}C\{^1H\}$ CP/MAS spectra were reference to adamantane where the upfield methane resonance was taken to be δ_C = 29.5 ppm[7] on a scale secondarily referenced to $\delta_C(SiMe_4)$ = 0.0 ppm. Simulated spectra were generated through the Spin Simulation function on MestReNova.

S2.3 X-Ray Diffraction

Single-crystal X-ray diffraction data for complexes **[^{t}Bu-NBD][BAr$^{F}_{4}$]** and **[^{t}Bu-*exo*-NBA][BAr$^{F}_{4}$]** were collected on Oxford Diffraction/Agilent SuperNova diffractometers with Cu-Kα (λ = 1.54184 Å) radiation equipped with nitrogen gas Oxford Cryosystems Cryostream unit**[8]** at the Oxford Chemical Crystallography Service from the University of Oxford. Diffraction images from raw frame data were reduced using CrysAlisPro.**[9]** The structures were solved using SHELXT**[10]** and refined to convergence on F^2 and against all independent reflections by full-matrix least-squares using SHELXL**[11]** (version 2018/3) in combination with the OLEX2**[12]** GUI. All non-hydrogen atoms were refined anisotropically and hydrogen atoms were geometrically placed and allowed to ride on their parent atoms. Disorder of the CF_3 groups on the $[BAr^{F}_{4}]^{-}$ anion was treated by introducing a split-site model and restraining geometries and displacement parameters. Distances and angles were calculated using the full covariance matrix. Selected crystallographic data and refinement data are summarized in the text and full details are given in the supplementary deposited CIF files (CCDC 2035752-2035753). These data can be obtained free of charge from the Cambridge Crystallographic Data Centre via http://www.ccdc.cam.ac.uk/data_request/cif.

S2.4 Mass Spectrometry and Microanalysis

Electrospray ionisation mass spectrometry (ESI-MS) was carried out using a Bruker MicrOTOF instrument directly connected to a modified Innovative Technology glovebox.[13] The spectrometer was calibrated using a mixture of tetraalkyl ammonium bromides $[N(C_nH_{2n+1})_4][Br]$ (n = 2-8, 12, 16, and 18). Samples were diluted to a concentration of at most 10^{-6} mol dm^{-3} before mass spectrometry. Samples were typically acquired with a 4 µL min^{-1} flow rate, a 0.4 bar nebuliser gas pressure, a flow of argon at 333 K with a 4 L min^{-1} flow rate to act as a drying gas, a capillary voltage of 4.5 kV, and an exit voltage of 60 V. Elemental microanalyses were all carried out by Dr Stephen Boyer of London Metropolitan University.

S3 Ligand and Complex Synthesis

S3.1 Synthesis of $[Rh(nbd)_2][BAr^{F}_{4}]$

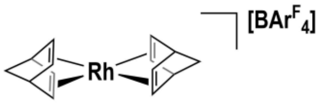

The following procedure improves the overall yield and reduces the side reactions that result in linear polymers during the reaction and workup.

A solution of $[\{Rh(\mu\text{-}Cl)(nbd)\}_2]$ (522 mg, 1.13 mmol) and 2,5-norbornadiene (810 µL, 7.94 mmol) in dichloromethane (10 mL) was added dropwise over 30 mins to a stirring suspension of $NaBAr^{F}_{4}$ (2.01 g, 2.27 mmol) also in dichloromethane (15 mL) at 0 °C. The reaction was then warmed to ambient temperature and stirred for 2 hours. The resulting in a red suspension was filtered and washed with two portions of dichloromethane (2 x 10 mL). Pentane (~50 mL) was then added to the filtrate to induce the precipitation of the product which was isolated by filtration, washed with pentane (2 x 15 mL) and dried under vacuum overnight to give the product as a red powder (1.8 g, 69 %).

S3.2 Synthesis of $P^{t}Bu_2CH_2CH_2CH_2P^{t}Bu_2$

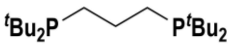

The following modifications to the literature procedure eliminated the need for ^{t}BuLi and avoid the need for a deoxygenated aqueous work up, which improved the overall yield from 41 to 89 %.

^{n}BuLi (2.5 M in hexane, 2.2 mL, 5.4 mmol) was added dropwise over 15 mins to a stirring solution of $^{t}Bu_2PH$ (1 mL, 5.4 mmol) in tetrahydrofuran (10 mL) resulting in a bright yellow solution. The reaction mixture was stirred at room temperature for 2 hours and then cooled to -78 °C and a solution of 1,3-dibromopropane (275 µL, 2.7 mmol) in tetrahydrofuran (~5 mL, degassed prior to use) was added. The reaction mixture was left to stir at -78 °C for 15 mins then allowed to warm up to ambient temperature overnight (18 hrs). The solvent and reaction volatiles were removed *in vacuo* to give a sticky white residue. The product was extracted into pentane (3 x 20 mL) and the solvent removed *in vacuo* to give the title compound as a viscous oil (300 mg, 89 %). ^{1}H and $^{31}P\{^{1}H\}$ spectroscopy matched what has been previously reported.[14]

S3.3 Synthesis and Characterisation of [^{t}Bu-NBD][BAr$^F{}_4$]

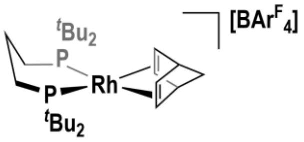

A solution of ^{t}Bu$_2$PCH$_2$CH$_2$CH$_2$P^{t}Bu$_2$ in pentane (0.10 M, 4.2 mL, 0.42 mmol) was added to a stirring solution of [Rh(nbd)$_2$][BAr$^F{}_4$] (480 mg, 0.417 mmol) in dichloromethane (10 mL). The reaction mixture was left to stir at room temperature for 1 hour when pentane (~40 mL) was added to precipitate the product. The product was isolated by cannula filtration and washed with two portions of pentane (~10 mL). The product was then recrystallised from dichloromethane layered with excess pentane at room temperature. Crystalline yield (380 mg, 65%).

^{1}H Solution NMR (400 MHz, CD$_2$Cl$_2$, 298 K): δ 7.73 (8H, s, *o*-BAr$^F{}_4$), 7.57 (4H, s, *p*-BAr$^F{}_4$), 5.25 (4H, m, alkene-CH$_{nbd}$), 3.97 (2H, bridgehead-nbd), 1.90 (2H, m, C*H$_2$*), 1.71 (4H, m, C*H$_2$*), 1.62 (2H, br s, C*H$_2$*), 1.35 (36H, d $J_{P\text{-}H}$ = 13.7 Hz, ^{t}Bu).

^{31}P{^{1}H} Solution NMR (162 MHz, CD$_2$Cl$_2$, 298 K): δ 24.3 (d, $J_{Rh\text{-}P}$ = 151.2 Hz)

^{13}C{^{1}H} solution NMR (125 MHz, CD$_2$Cl$_2$, 298 K): δ 162.2 (q, $^1J_{C\text{-}B}$ = 49.9 Hz, *ipso*-BAr$^F{}_4$), 135.2 (s, *p*-BAr$^F{}_4$), 129.3(qq, $^2J_{F\text{-}C}$ = 32, $^2J_{C\text{-}B}$ = 2 Hz, *o*-BAr$^F{}_4$), 125.1(q, $^1J_{F\text{-}C}$ = 275 Hz, -*C*F$_3$), 117.9 (m, *m*-BAr$^F{}_4$), 70.6 (m, =*C*H$_{nbd}$), 68.9 (m, nbd-*C*H$_2$), 52.8 (m, nbd-*C*H), 39.6 (m, P*C*(CH$_3$)$_3$), 30.7 (br m, PC(*C*H$_3$)$_3$), 22.9 (br m, CH$_2$), 20.6 (m, CH$_2$).

^{31}P{^{1}H} SSNMR (162 MHz, 10 kHz spin rate, 298 K): δ 21.5 (d, $J_{Rh\text{-}P}$ = 176 Hz), 20.4 (d, $J_{Rh\text{-}P}$ = 162 Hz),

^{13}C{^{1}H} SSNMR (101 MHz, 10 kHz spin rate, 298 K): δ 165.4, 163.2, 136.8, 134.0, 132.4, 130.2, 124.2, 117.1(BAr$^F{}_4$), 79.7, 79.2, 65.2, 64.3, 59.6, 51.8, 51.0 (alkene and nbd CH, CH$_2$), 39.2, 38.3, 37.2, 30.6, 28.2, 27.8, 22.4, 19.8 (phosphine).

Elemental Analysis for C$_{58}$H$_{62}$BF$_{24}$P$_2$Rh$_1$ Calc. C 50.06, H 4.49. Found 49.96, 4.38

ESI-MS found (calc.): 527.2419 (527.2437) for [(^{t}Bu$_2$P(CH$_2$)$_3$P^{t}Bu$_2$)Rh(C$_7$H$_8$)]$^+$

Crystal Data for C$_{58}$H$_{62}$BF$_{24}$P$_2$Rh (*M* =1390.73 g/mol): monoclinic, space group P2$_1$/n (no. 14), *a* = 21.7222(2) Å, *b* = 12.84480(10) Å, *c* = 23.3497(2) Å, *β* = 112.0750(10)°, *V* = 6037.37(10) Å^3, *Z* = 4, *T* = 150.01(10) K, μ(CuKα) = 3.797 mm^{-1}, *Dcalc* = 1.530 g/cm^3, 67203 reflections measured (4.74° ≤ 2Θ ≤ 154.39°), 12715 unique (R_{int} = 0.0515, R$_{sigma}$ = 0.0309) which were used in all calculations. The final R_1 was 0.0418 (I > 2σ(I)) and wR_2 was 0.1121 (all data).

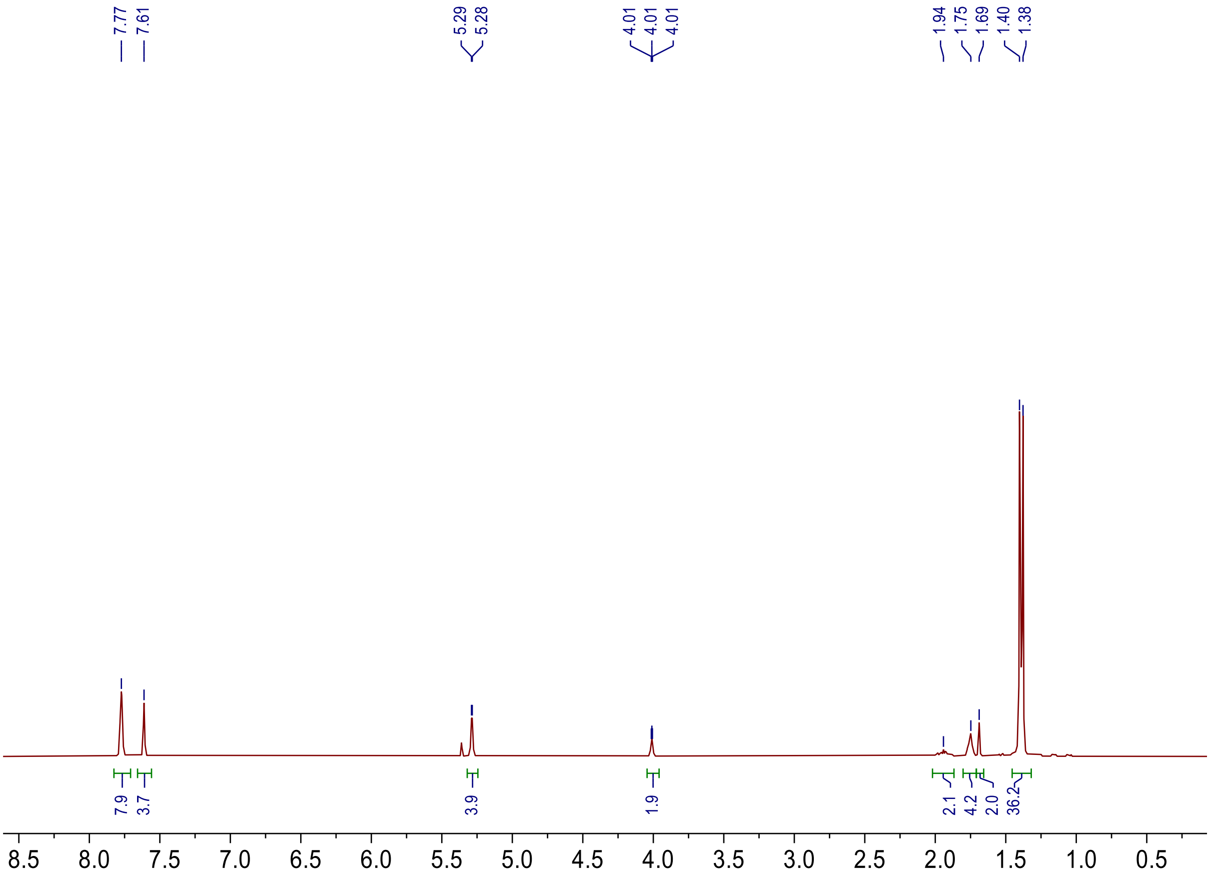

Figure S1: ^{1}H (400 MHz, CD_2Cl_2, 298K) Solution NMR Spectrum of **[^{t}Bu-NBD][BAr$^{F}_{4}$]**

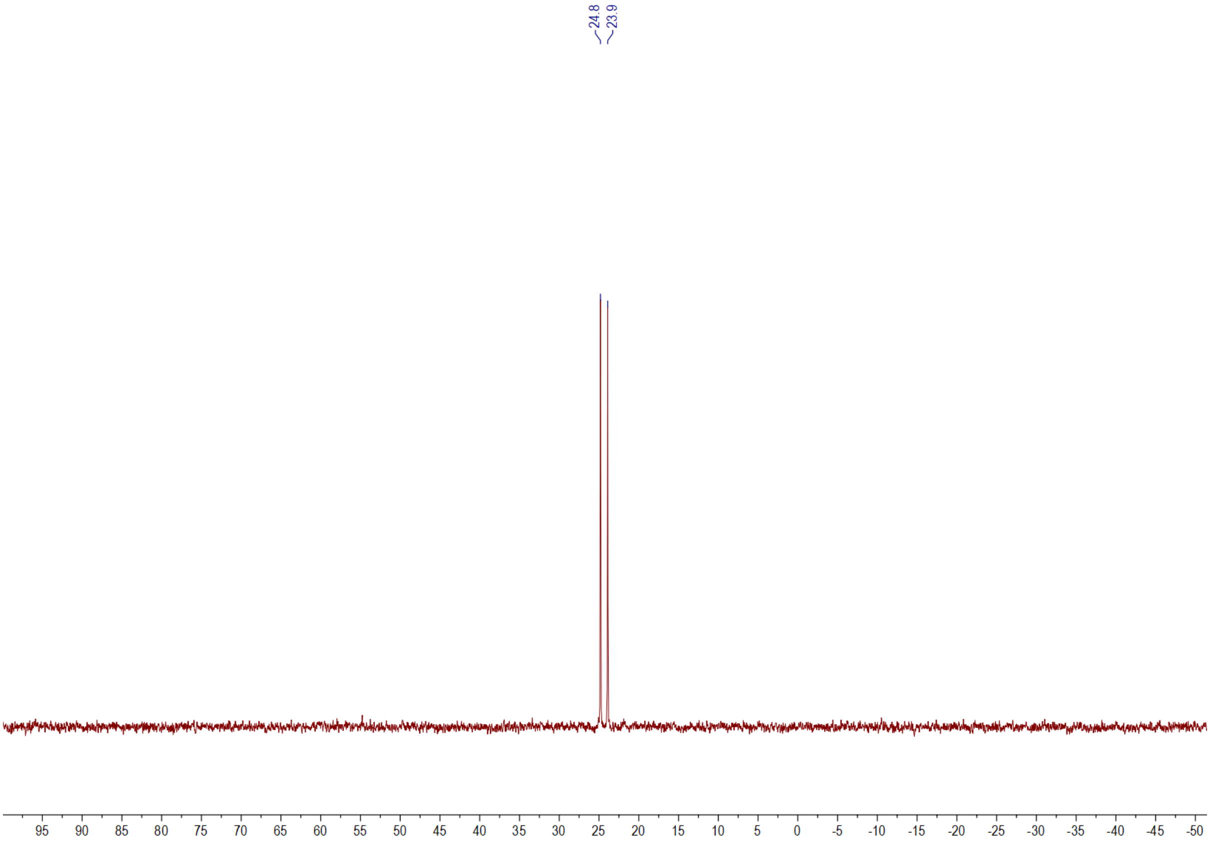

Figure S2: ^{31}P{^{1}H} (162 MHz, CD_2Cl_2, 298K) Solution NMR Spectrum of **[^{t}Bu-NBD][BAr$^{F}_{4}$]**

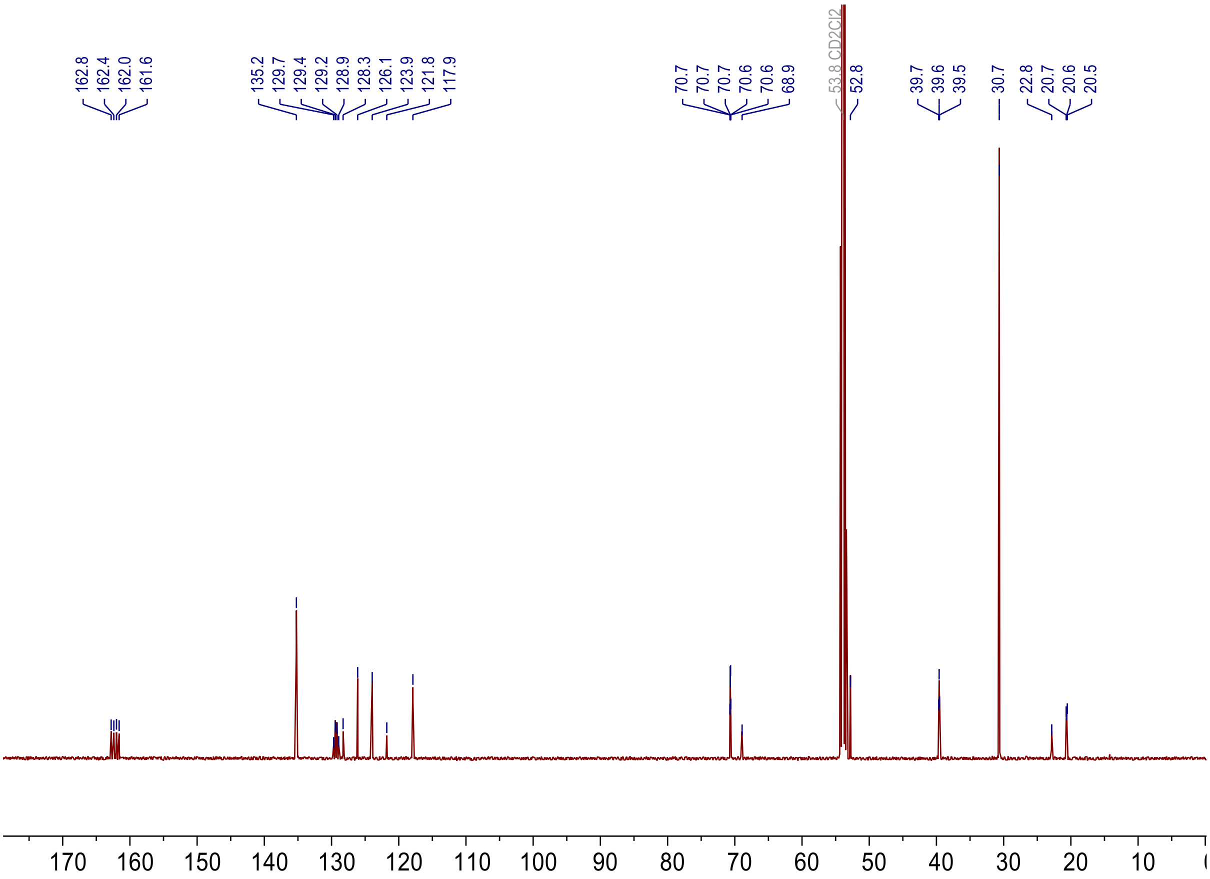

Figure S3: $^{13}C\{^1H\}$ (125 MHz, CD_2Cl_2, 298K) Solution NMR Spectrum of **[^{t}Bu-NBD][BAr^F$_4$]**

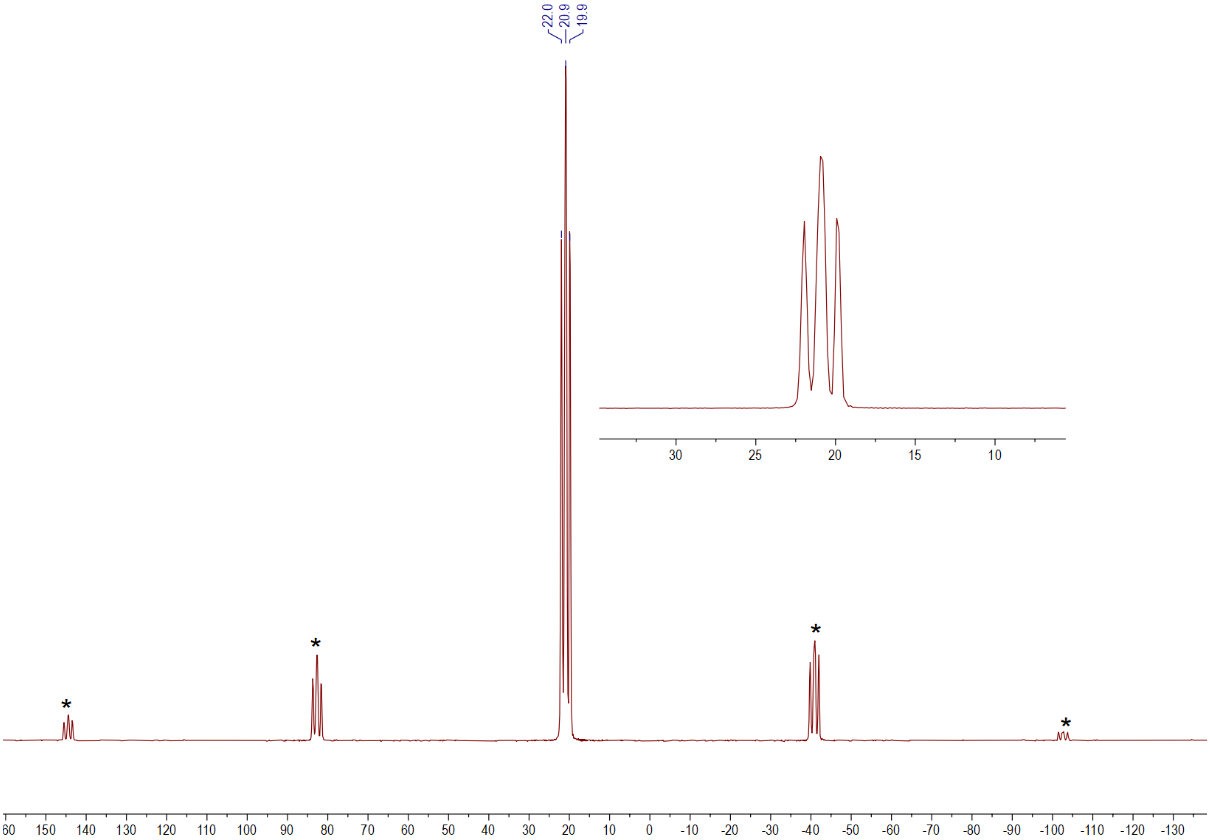

Figure S4: $^{31}P\{^1H\}$ (162 MHz, 10 kHz spin rate, 298 K) SSNMR Spectrum of **[^{t}Bu-NBD][BAr^F$_4$]**. *Denotes spinning side bands

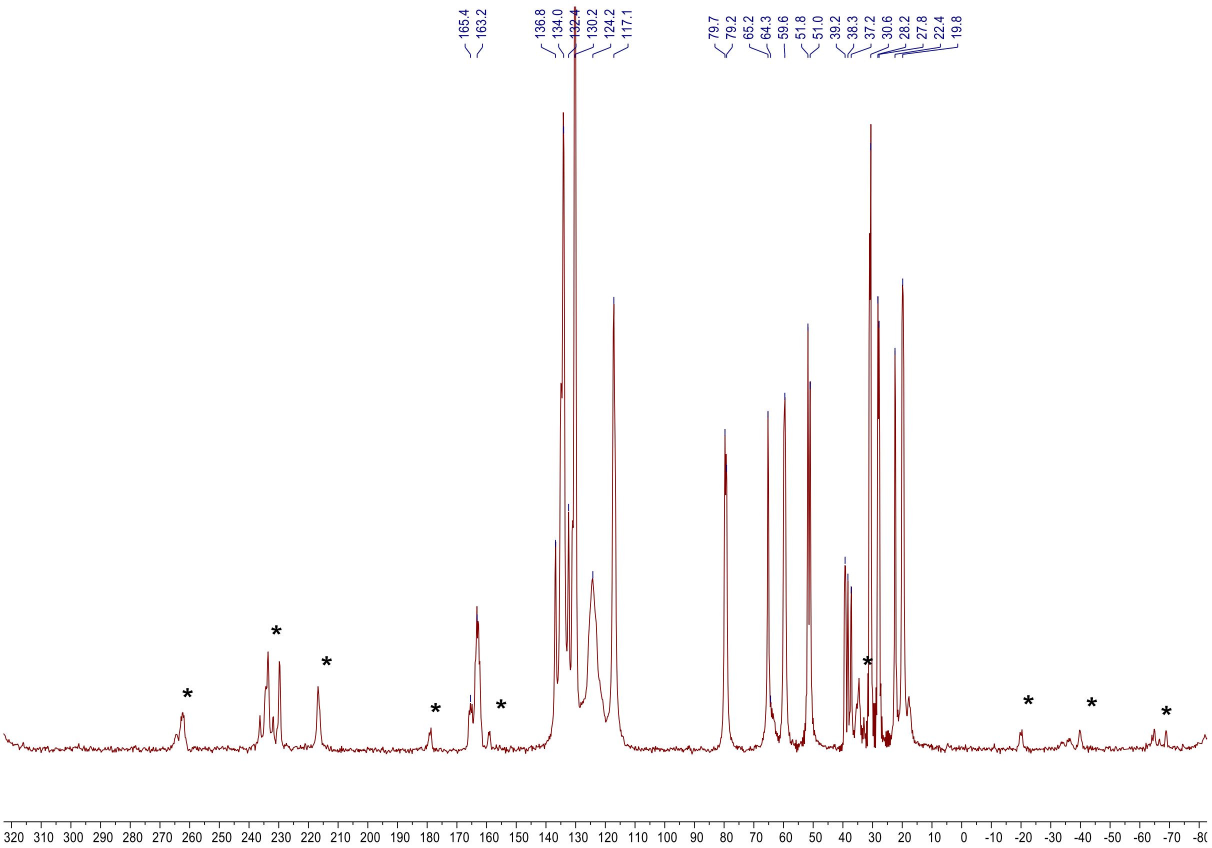

Figure S5: $^{13}C\{^{1}H\}$ (101 MHz, 10 kHz spin rate, 298 K) SSNMR Spectrum of **[^{t}Bu-NBD][BAr$^{F}{}_{4}$]**. *Denotes spinning side bands.

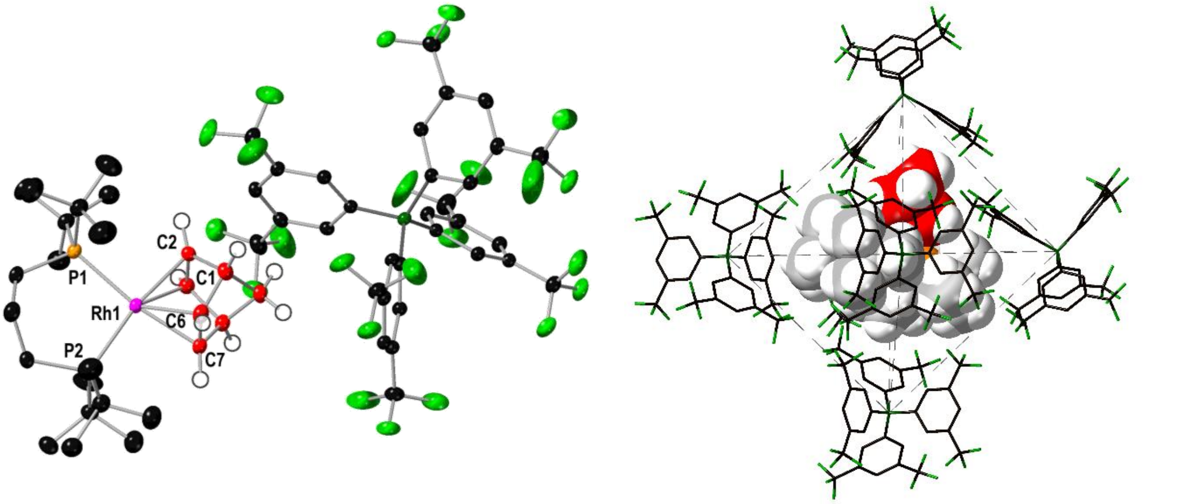

Figure S6: Molecular Structure of **[^{t}Bu-NBD][BAr$^{F}{}_{4}$]**. Hydrogen atoms displayed for NBD ligand only (left). Displacement ellipsoids shown at the 35% probability levels. Selected bond lengths (Å) Rh1-P1, 2.3926(7); Rh1-P2, 2.4006(7); Rh1-C7, 2.188(3); Rh1-C2, 2.190(3); Rh1-C1, 2.172(3); Rh1-C6, 2.171(3); C2-C1, 1.381(4); C7-C6, 1.380(4).

S3.4 Synthesis and Characterisation of [^{t}Bu-*exo*-NBA][BAr$^{F}{}_{4}$]

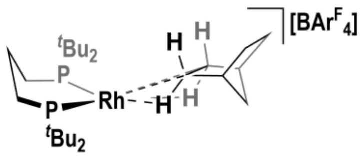

Crystals of **[^{t}Bu-NBD][BAr$^{F}{}_{4}$]** were placed under 1 bar (298 K) of hydrogen for 10 minutes in a J. Young stopcock NMR tube to form [($^{t}Bu_2PCH_2CH_2CH_2P^{t}Bu_2$)Rh($\eta^2\eta^2$-$C_7H_{12}$)][BAr$^{F}{}_{4}$] and selected crystals were immediately cooled to 150 K on the diffractometer. SSNMR samples were prepared *ex situ* by placing 65 mg of crushed crystalline material of complex

[^{t}Bu-NBD][BAr^F$_4$] in a small J. Young ampoule, which was then placed under 1 bar of hydrogen for 10 mins. The atmosphere was then replaced with argon and cycled into a glovebox and the material packed into the rotor; the first spectrum collected was after 30 mins. Aged SSNMR samples were prepared in the same manner except after the hydrogenation time was complete, the hydrogen was removed and replaced with argon for the appropriate amount of time before the sample was packed into the rotor and spectra collected.

^{31}P{^{1}H} SSNMR (162 MHz, 10 kHz spin rate, 298 K): δ 64.4 (doublet, J_{Rh-P} = 205 Hz), 63.2 (doublet, J_{Rh-P} = 195 Hz).

^{13}C{^{1}H} SSNMR (101 MHz, 10 kHz spin rate, 285 K): δ 163.8, 136.3, 134.7, 131.5, 125.3, 118.5, 117.6 (BAr^F$_4$), 39.0, 37.8, 31.1, 30.2, 23.6, 18.3 (nba and phosphine).

^{13}C{^{1}H} SSNMR (101 MHz, 10 kHz spin rate, 158 K): δ 162.2, 135.0, 133.0, 130.00, 123.9, 120.0, 116.4 (BAr^F$_4$), 37.8, 35.6, 28.9, 26.8, 25.4, 24.0, 22.0, 20.5, 16.7 (nba and phosphine).

Elemental Analysis for $C_{58}H_{66}BF_{24}P_2Rh_1$ Calc. C 49.95, H 4.77. Found 49.50, 4.72

Crystal Data for $C_{58}H_{66}BF_{24}P_2Rh$ (M =1394.76 g/mol): monoclinic, space group P2$_1$/n (no. 14), a = 22.2442(11) Å, b = 12.9007(3) Å, c = 23.1254(10) Å, β = 110.850(5)°, V = 6201.6(5) Å^3, Z = 4, T = 150.01(10) K, μ(CuKα) = 3.697 mm^{-1}, $Dcalc$ = 1.494 g/cm^3, 50590 reflections measured (4.734° ≤ 2Θ ≤ 136.58°), 11234 unique (R_{int} = 0.0857, R_{sigma} = 0.0798) which were used in all calculations. The final R_1 was 0.0751 (I > 2σ(I)) and wR_2 was 0.2157 (all data).

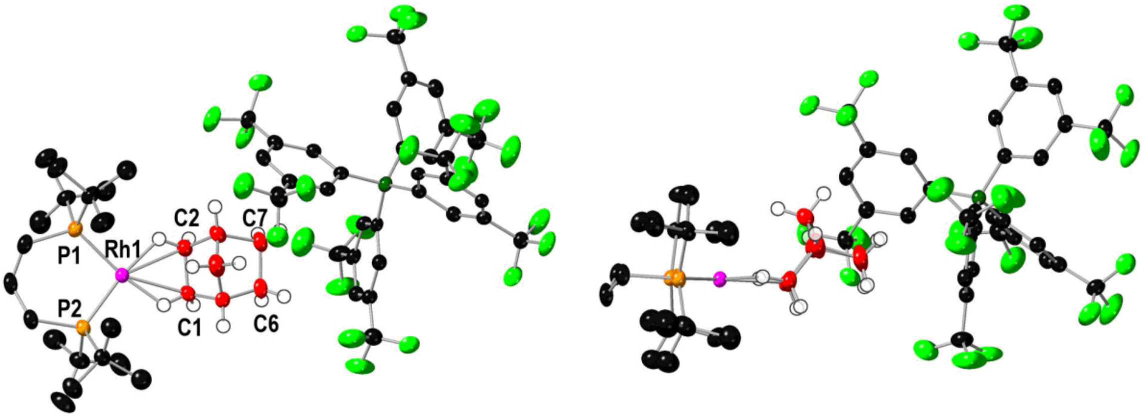

Figure S7: Molecular Structure of **[^{t}Bu-exo-NBA][BAr^F$_4$]**, top view (left) and side view (right). Hydrogen atoms displayed for NBA ligand only. Displacement ellipsoids shown at the 35% probability levels. Selected bond lengths (Å) Rh(1)-P(1) 2.2437(18), Rh(1)-P(2) 2.235(2), Rh(1)-C(2) 2.382(7), Rh(1)-C(1) 2.363(7), C(2)-C(1) 1.534(10), C(7)-C(6) 1.526(12).

S3.5 Volatile Trapping Experiments

Crushed **[^{t}Bu-NBD][BAr^F$_4$]** (~20 mg) was placed in J. Young ampoule, which was then placed under 1 bar of hydrogen for 10 mins. The atmosphere was then replaced with argon and aged for the appropriate time (1 hr or 24 hrs). CD_3CN (0.6 cm^3) was vacuum transferred into the ampoule and the resulting yellow solution warmed to ambient temperature to ensure all the compound had dissolved. The volatiles were then vacuum transferred into a J. Young NMR tube. ^{1}H Solution NMR (500 MHz, CD_3CN, 298 K): δ 2.21 (b, 2H), 1.50 (m, 4H), 1.24-1.18 (m, 6H). Literature values = δ 2.21, 1.52, 1.24, 1.21 (MeOD).[15]

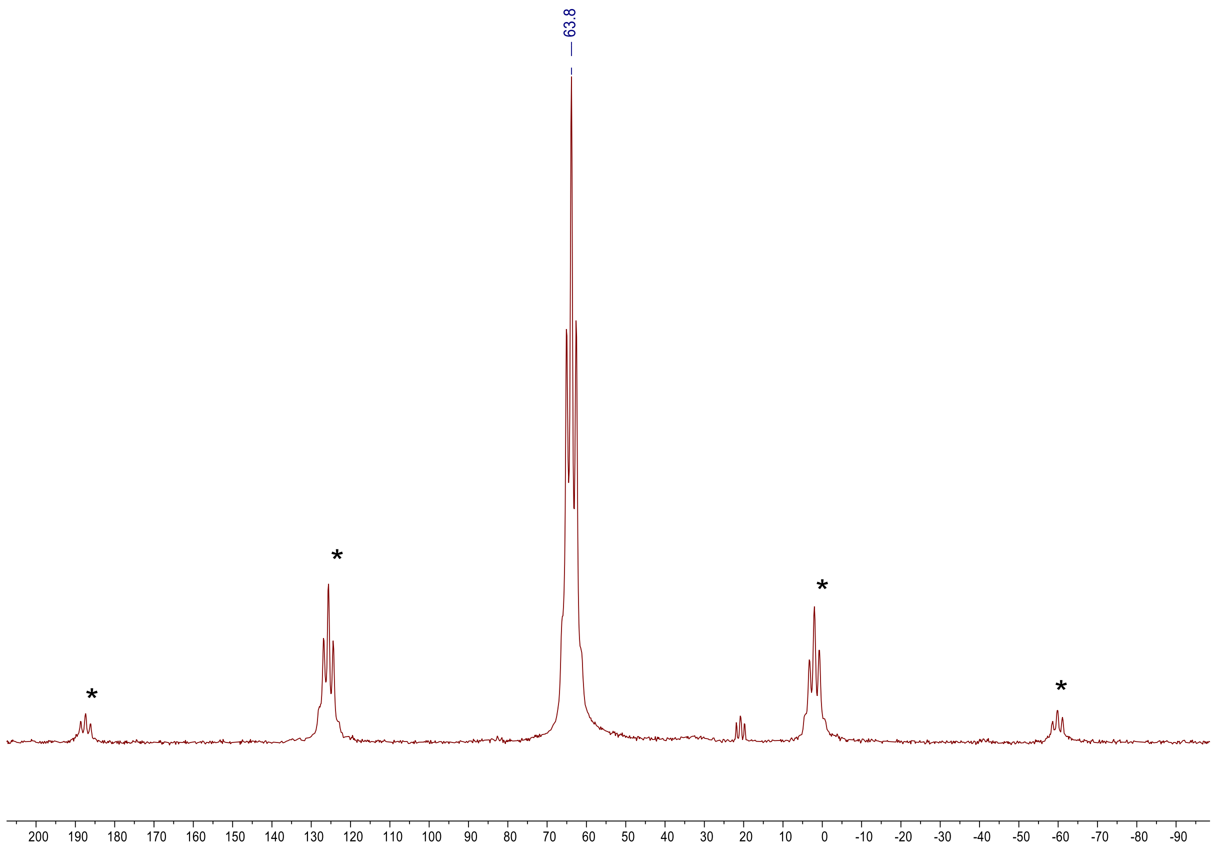

Figure S8: $^{31}P\{^{1}H\}$ (162 MHz, 10 kHz spin rate, 298 K) SSNMR Spectrum of **[^{t}Bu-exo-NBA][BAr$^{F}_{4}$]**. *Denotes spinning side bands. Peak at 20.9 is unreacted **[^{t}Bu-NBD][BAr$^{F}_{4}$]** (< 5%).

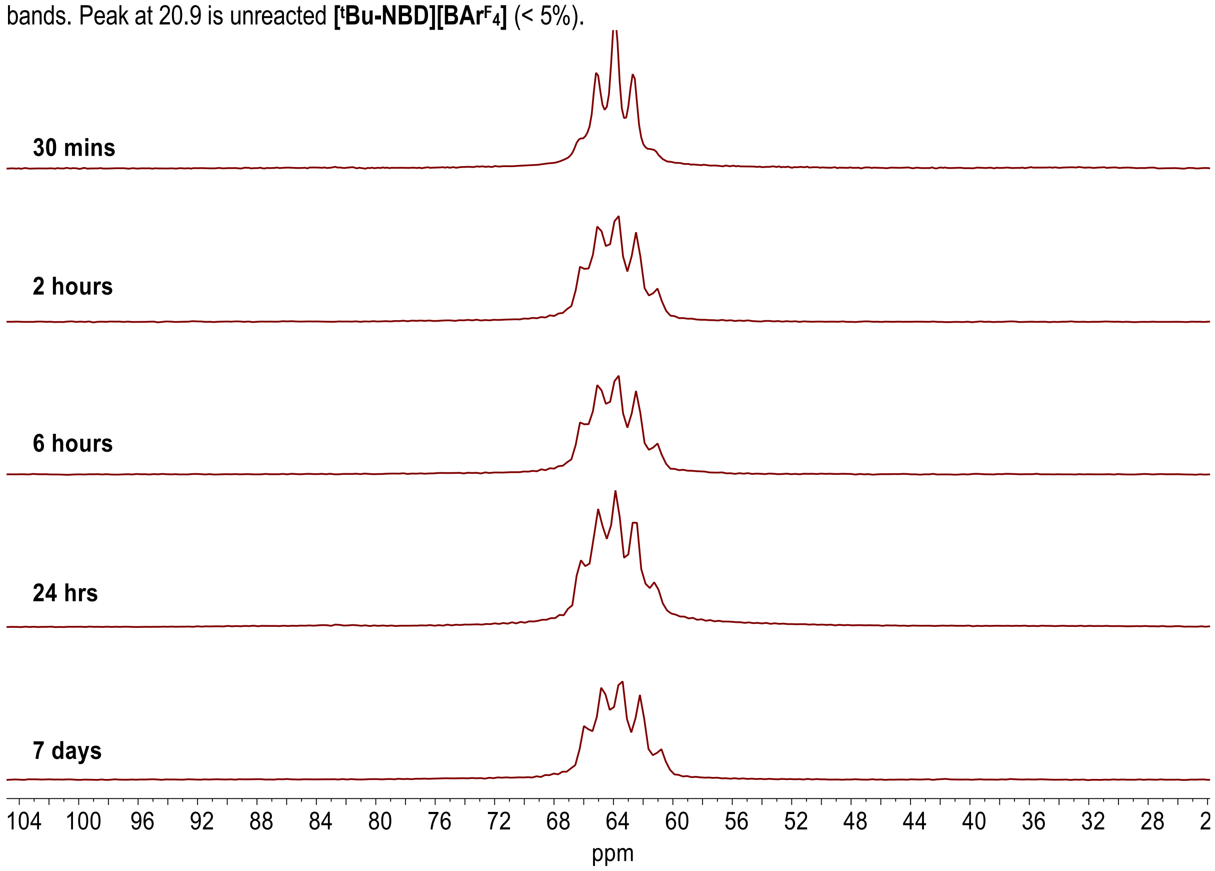

Figure S9: $^{31}P\{^{1}H\}$ (162 MHz, 10 kHz spin rate, 298 K) SSNMR Spectra of **[^{t}Bu-exo-NBA][BAr$^{F}_{4}$]** over time.

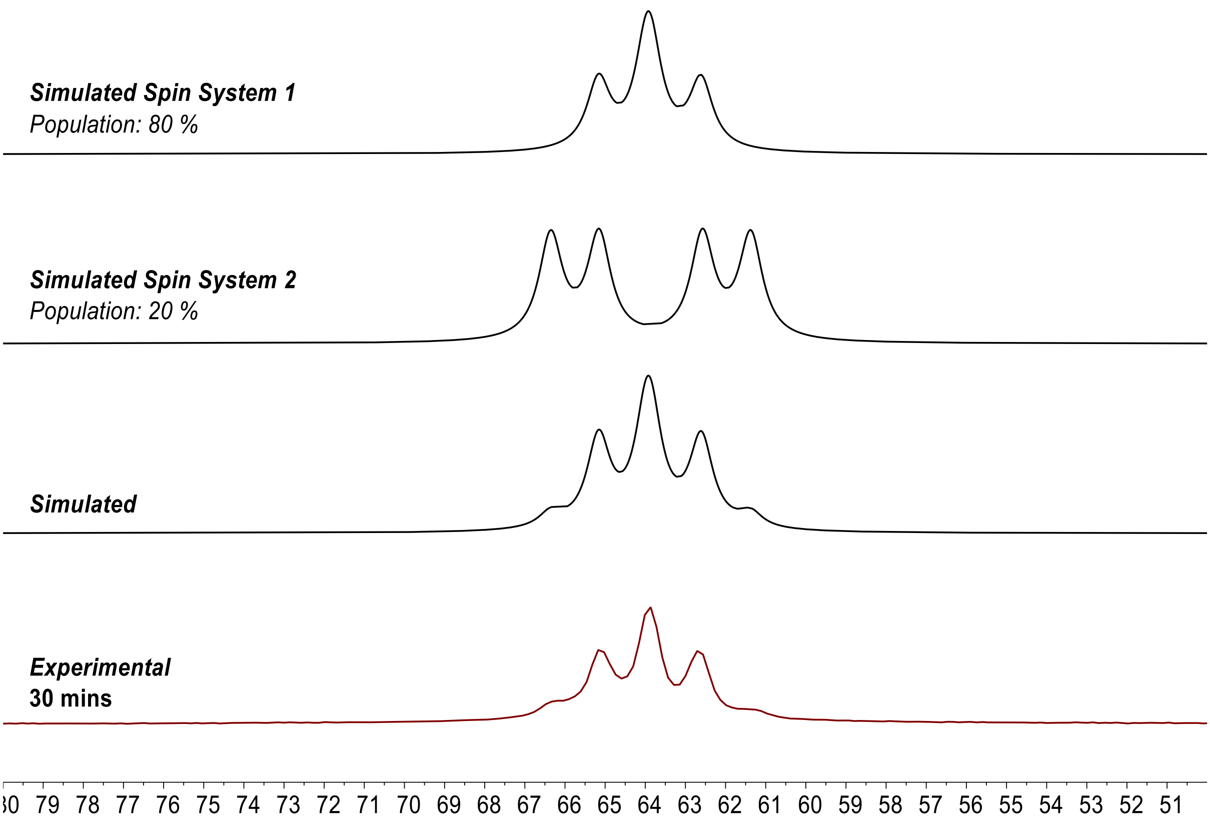

Figure S10: Simulated $^{31}P\{1H\}$ SS NMR Spectrum of **[^{t}Bu-*exo*-NBA][BAr$^{F}_{4}$]** at 30 mins showing the approximated populations of the two different spin systems.

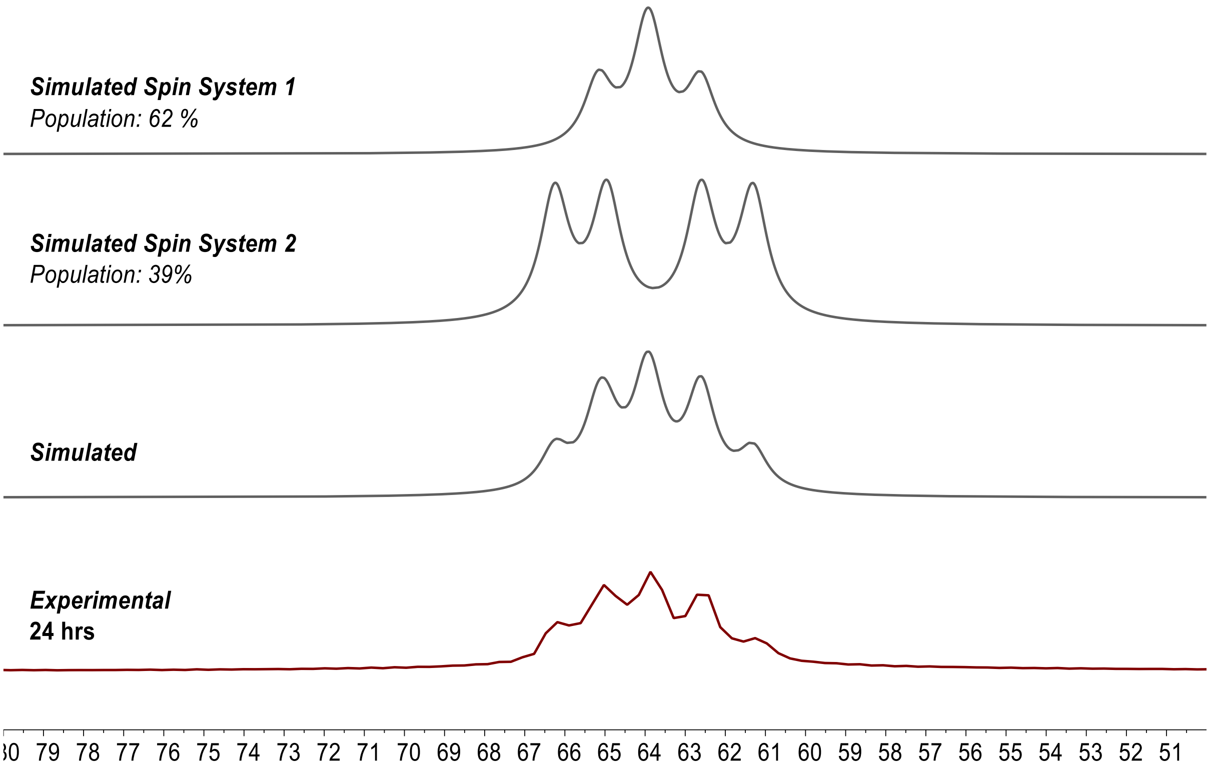

Figure S11: Simulated $^{31}P\{1H\}$ SS NMR Spectrum of **[^{t}Bu-exo-NBA][BAr$^{F}_{4}$]** at 24 hours showing the approximated populations of the two different spin systems.

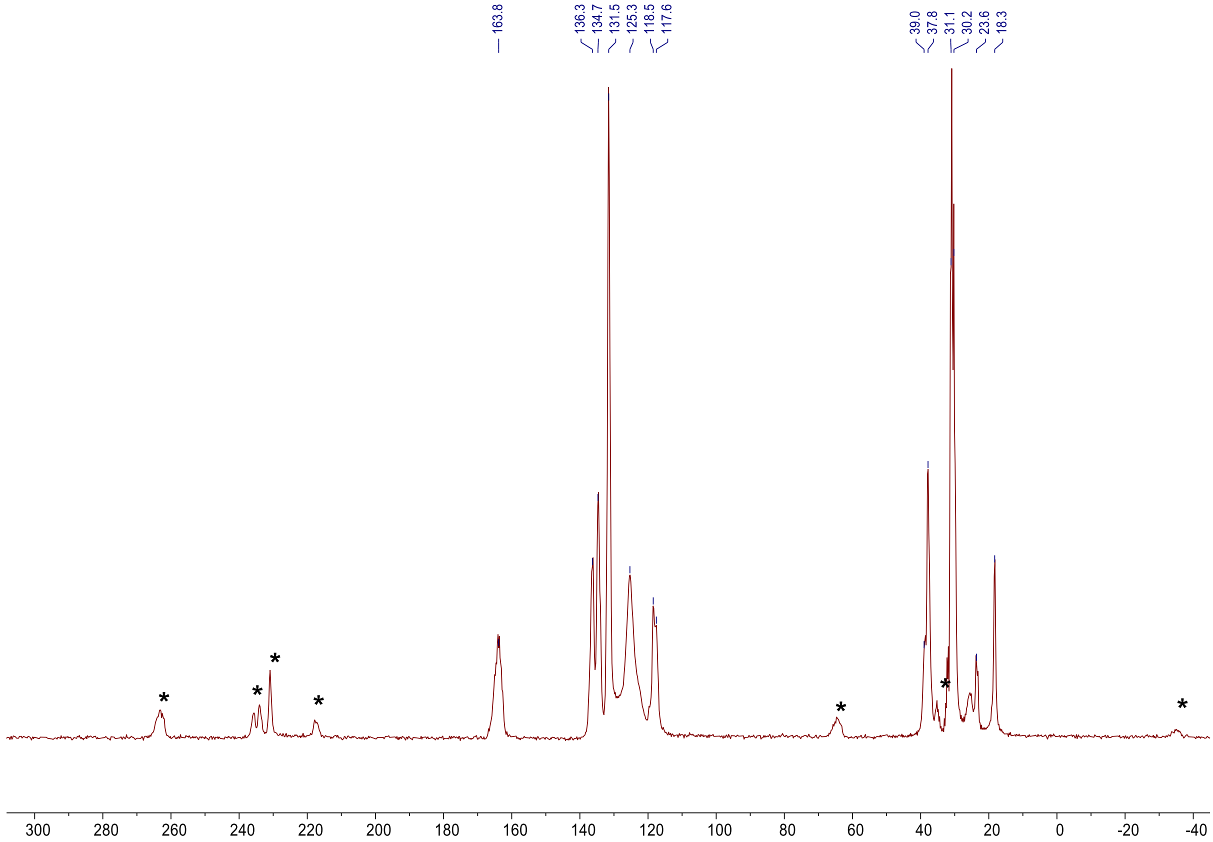

Figure S12: $^{13}C\{^{1}H\}$ (100 MHz, 10 kHz spin rate, 285 K) SSNMR Spectrum of **[^{t}Bu-exo-NBA][BAr$^{F}_{4}$]**. *Denotes spinning side band

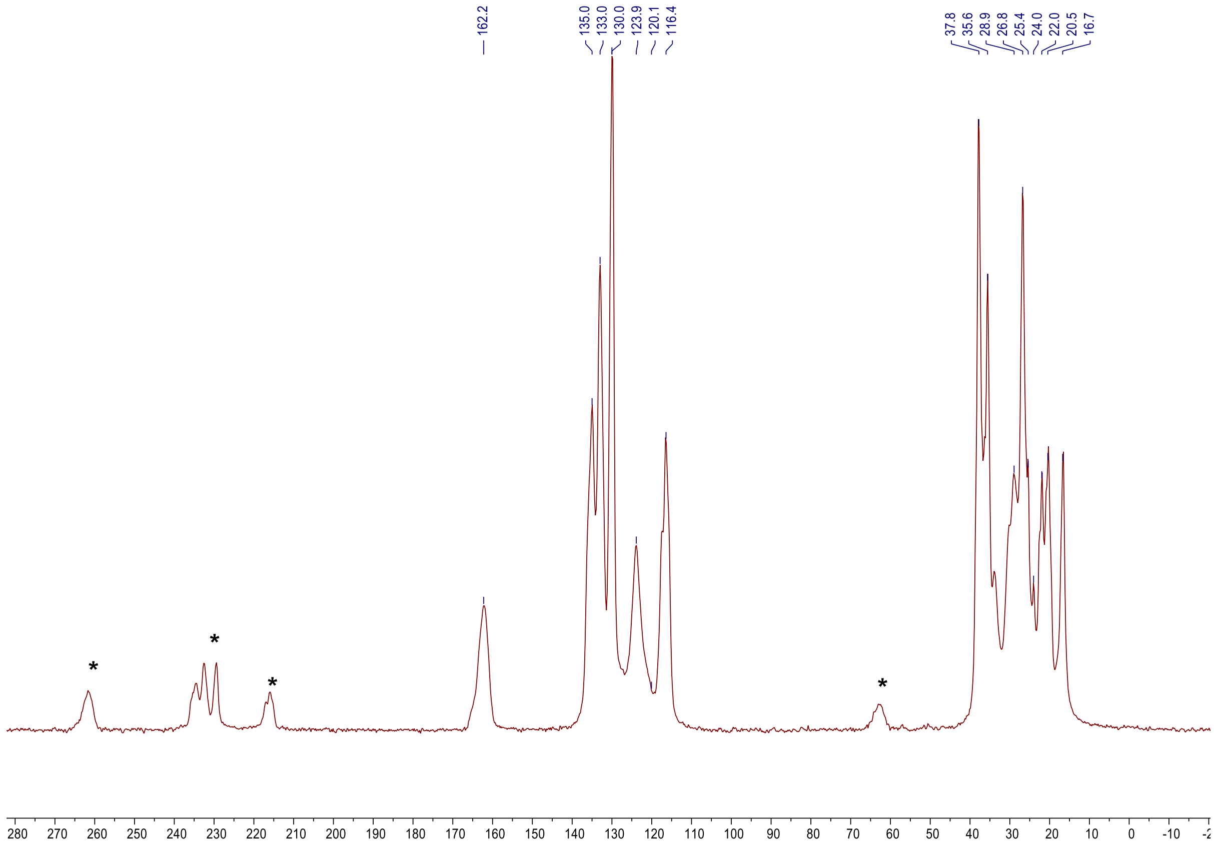

Figure S13: $^{13}C\{^{1}H\}$ (100 MHz, 10 kHz spin rate, 158 K) SSNMR Spectrum of **[^{t}Bu-exo-NBA][BAr$^{F}_{4}$]**. *Denotes spinning side band

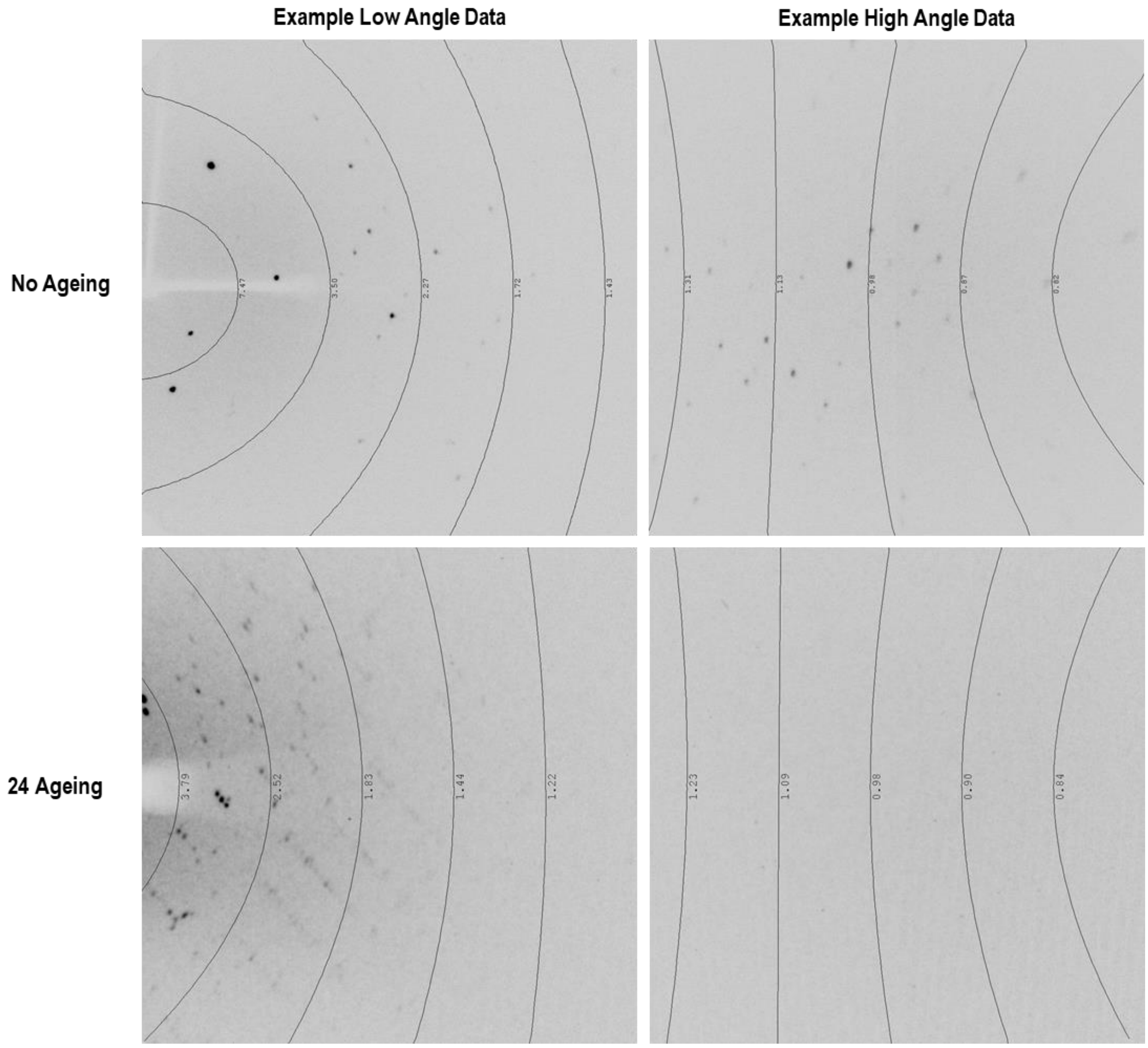

Figure S14: Low angle and high angle data frames of non-aged (top) and aged (bottom, aged under argon) crystals of **[^{t}Bu-exo-NBA][BAr^F$_4$]**. Crystals of **[^{t}Bu-NBD][BAr^F$_4$]** were placed into a J Youngs NMR tube and placed under 1 bar of hydrogen for 10 mins to generate **[^{t}Bu-exo-NBA][BAr^F$_4$]** and non-aged samples were immediately transferred to the diffractometer upon the completion of hydrogenation. Aged samples had the hydrogen atmosphere removed by vacuum after the hydrogenation time and the crystals placed under argon for 24 hrs before transferred to the diffractometer.

S4 1-Butene Isomerisation

S4.1 Batch Conditions

Gas phase ^{1}H NMR spectra were obtained using Bruker Avance III HD NMR spectrometer at room temperature and referenced to reported gas phase data for methane with T_1 = 1.0 s.[9] The spectrometer was pre-locked and pre-shimmed to a separate sample of CD_2Cl_2 before NMR study of the gaseous NMR sample. Samples were prepared by placing a finely crushed sample (2.0 mg, 1.4 μmol) into a J. Young stopcock NMR tube and the sample placed under an atmosphere of hydrogen (1.0 bar) for the appropriate amount of time (10 mins for **[^{t}Bu-NBD][BAr^F$_4$]** and 5 mins for **[Cy-NBD][BAr^F$_4$]**). The hydrogen atmosphere was removed by vacuum and replaced with 1-butene (1.0 bar, 2.0 cm^3, 83 μmol). Spectra was acquired as quickly as possible.

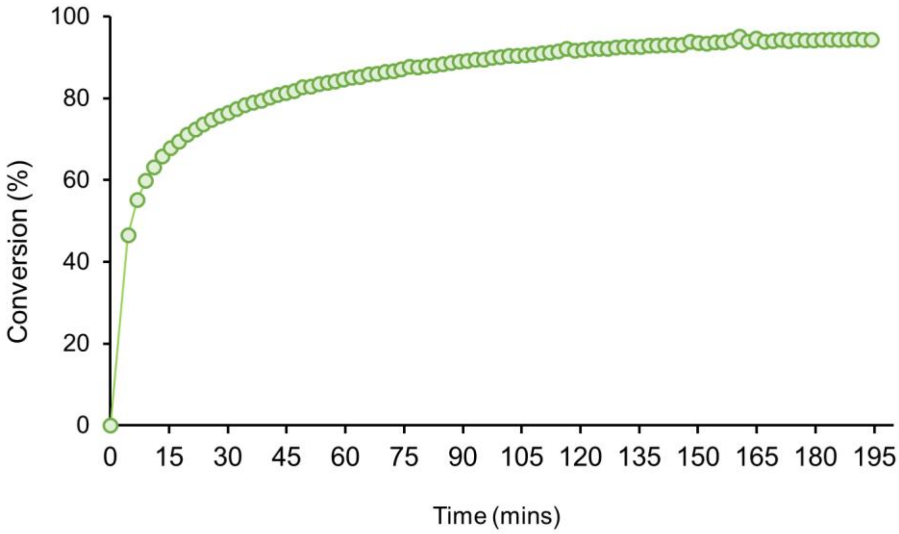

Figure S15: Catalytic performance of 1-butene isomerisation by **[^{t}Bu-exo-NBA][BAr$^F_4$]** in solid/gas catalysis. Data plotted as total 2-butenes conversion as measured by ^{1}H NMR Spectroscopy.

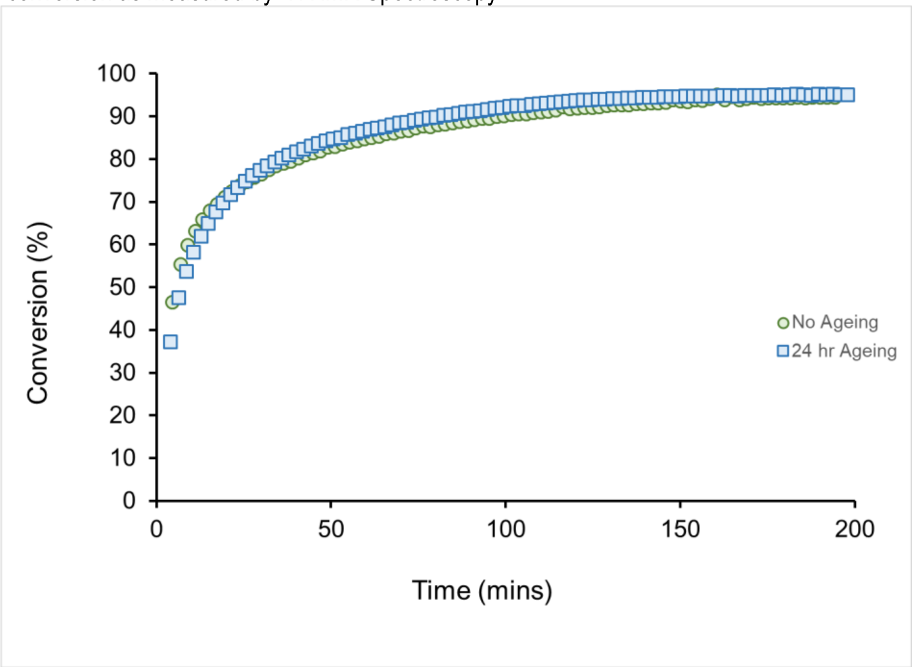

Figure S16: Catalytic performance of 1-butene isomerisation by aged and non-aged samples of **[^{t}Bu-exo-NBA][BAr$^F_4$]** in solid/gas catalysis. Data plotted as total 2-butenes conversion as measured by ^{1}H NMR Spectroscopy. Round green circles are non-aged samples, blue squares are samples of **[^{t}Bu-exo-NBA][BAr$^F_4$]** aged under argon for 24 hrs after hydrogenation before addition of 1-butene.

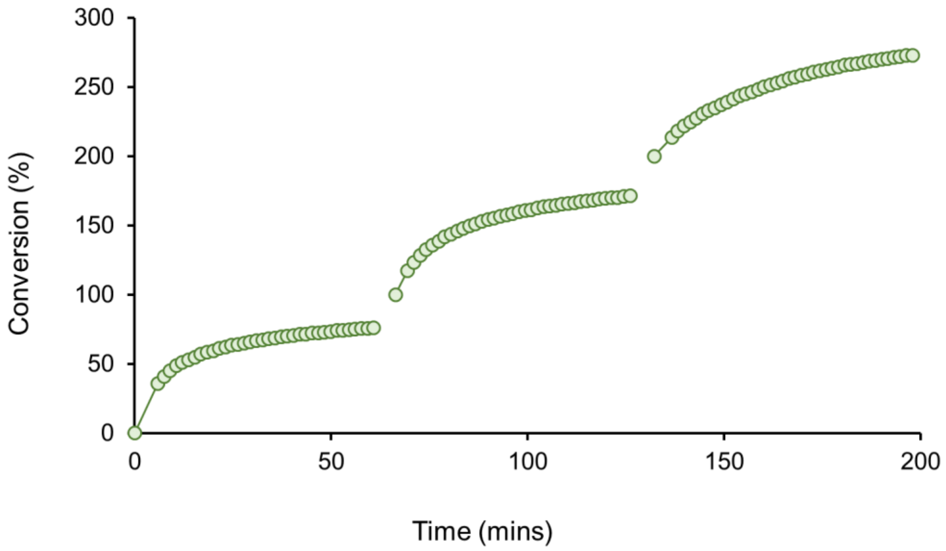

Figure S17: Catalytic performance of 1-butene isomerisation of **[^{t}Bu-exo-NBA][BAr$^{F}_{4}$]** in solid/gas catalysis recharging the 1-butene atmosphere at 60 min intervals. Data plotted as total 2-butenes conversion as measured by ^{1}H NMR spectroscopy.

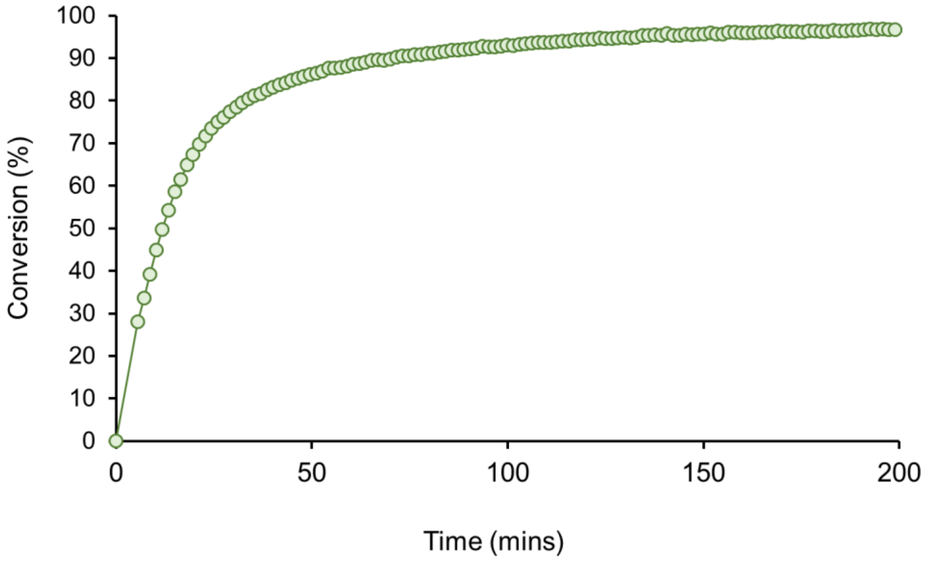

Figure S18: Catalytic performance of 1-butene isomerisation by **[Cy-endo-NBA][BAr$^{F}_{4}$]** in solid/gas catalysis. Data plotted as total 2-butenes conversion as measured by ^{1}H NMR Spectroscopy.

S4.2 1-Butene Addition to [^{t}Bu-*exo*-NBA][BAr$^{F}_{4}$] Experiments

Samples were prepared for solution NMR experiments by placing a finely crushed sample (20 mg) into a J. Young stopcock NMR tube and the sample placed under an atmosphere of hydrogen (1 bar) for 10 mins. The hydrogen atmosphere was removed and replaced with 1-butene for 1 hour or 24 hours. The atmosphere of excess butenes was removed and CD_2Cl_2 was vacuum transferred into the sample and kept at 183 K until it was loaded into an NMR spectrometer pre-cooled to 183 K. Samples were prepared for SSNMR experiments by placing a finely crushed sample (65 mg) into a J. Young stopcock ampoule and the sample placed under an atmosphere of hydrogen (1 bar) for 10 mins. The hydrogen was quickly removed and replaced with 1-butene for 1 hour or 24 hours. The atmosphere of butenes was removed and replaced with argon, the sample was then quickly packed into a rotor in a glove box and spectra acquired soon after.

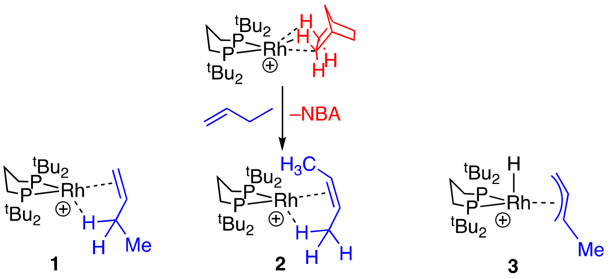

Diagnostic Resonances in ¹H Solution NMR (500 MHz, CD_2Cl_2, 183 K): *δ* 0.23 (s, 6H, **2:** agostic H), –0.14 (br s, 3H, **1:** CH_3), –0.19 (br s, 1H, **1:** C–H), –4.07 (br s, 1H, **1:** agostic H), –29.04 (br s, 1H, **3:** Rh-H).

Diagnostic Resonances in ¹H Solution NMR (500 MHz, CD_2Cl_2, 208 K): *δ* 0.32 (s, 6H, **2:** agostic H), -0.05 (br s, 3H: **1:** CH_3), -0.33 (br s, 1H **1:** C–H), -3.73 (s, 1H, **1:** agostic H), -28.92 (br dt, $^1J_{Rh\text{-}H}$ = 36, $J_{P\text{-}H}$ = 15 Hz, 1H, **3:** Rh-H).

¹H Solution NMR (500 MHz, CD_2Cl_2, 183 K): *δ* 7.70 (s, 24H, $BAr^F{}_4$), 7.50 (s, 12H, $BAr^F{}_4$), 5.71 (m, 1H), 5.33 (m, 1H, alkene H), 5.29 (br s, 2H, alkene H), 5.14 (m, 1H, alkene H), 5.07(m, 1H, alkene H), 4.59 (2H, alkene H), 3.91 (br s, 1H) 3.00 (d, 1H), 2.23 (br m, 5H), 2.04 (br, s, 1H), 1.92 (br m, 4H), 1.61-0.93 (multiple resonances, 128H), *δ* 0.23 (s, 6H, **2:** agostic H), –0.14 (br s, 3H, **1:** CH_3), –0.19 (br s, 1H, **1:** agostic H), –4.07 (br s, 1H, **1:** agostic H), –29.04 (br s, 1H, **3:** Rh-H, $^1J_{Rh\text{-}H}$ = 32 Hz, as observed in $^1H\{^{31}P\}$ spectrum).

$^{31}P\{^1H\}$ Solution NMR (162 MHz, CD_2Cl_2, 183 K): *δ* 67.1 (dd, $J_{Rh\text{-}P}$ = 203, $J_{P\text{-}P}$ = 35 Hz, **1:** *trans* to CH_2 of 1-butene), 64.2 (dd, $J_{Rh\text{-}P}$ = 216, $J_{P\text{-}P}$ 35 Hz, **2:** *trans* to CH_3 of 2-butene), 60.9 (dd, $J_{Rh\text{-}P}$ = 127, $J_{Rh\text{-}P}$ = 13 Hz, **3:** *trans* to allyl), 49.6 (dd, $J_{Rh\text{-}P}$ = 131, $J_{Rh\text{-}P}$ = 13 Hz, **3:** *trans* to allyl), 42.9 (dd, $J_{Rh\text{-}P}$ = 155, $J_{P\text{-}P}$ = 35 Hz, **1:** *trans* to C=C of 1-butene), 41.8 (dd, $J_{Rh\text{-}P}$ = 157, $J_{P\text{-}P}$ = 35 Hz, **2:** *trans* to C=C of 2-butene).

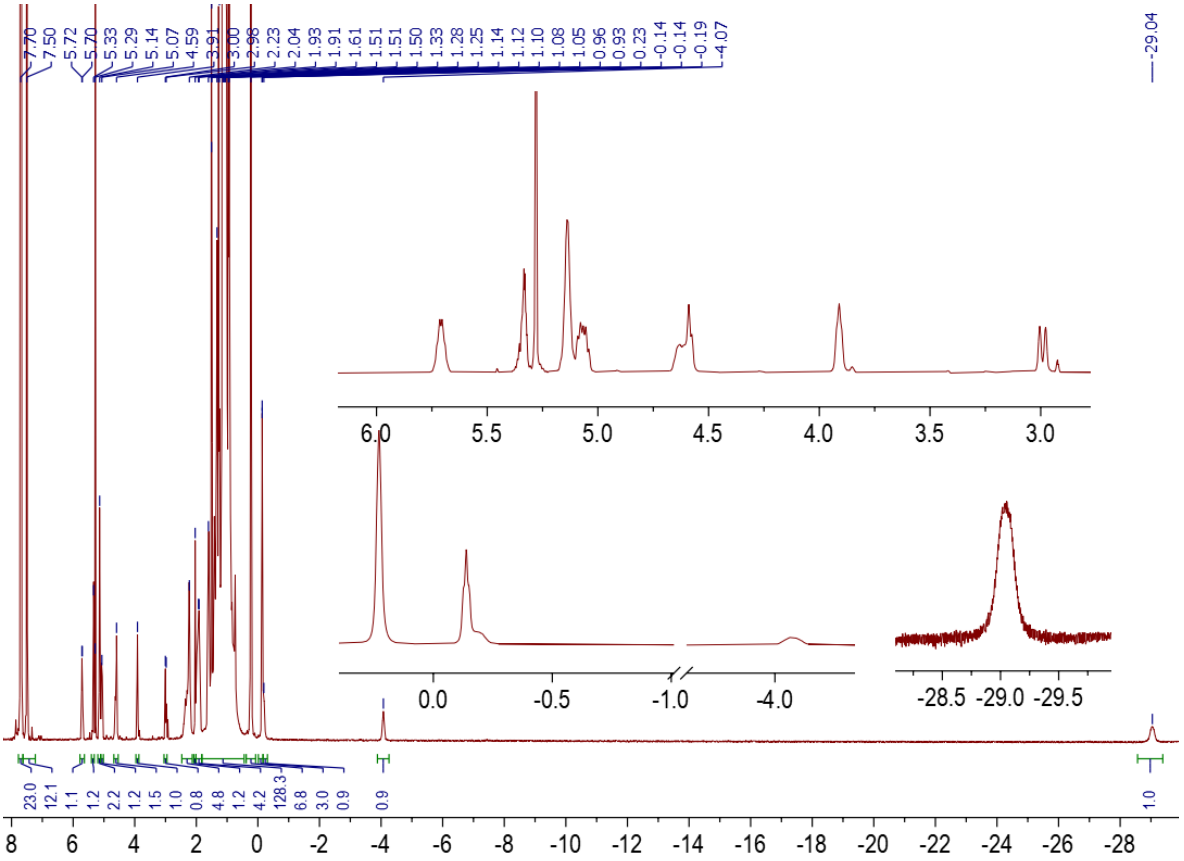

Figure S19: ¹H (500 MHz, CD_2Cl_2, 183K) Solution NMR Spectrum after the addition of 1-butene to **[ᵗBu-exo-NBA][BArᶠ₄]** in the solid-state and dissolved into CD_2Cl_2 at 183 K.

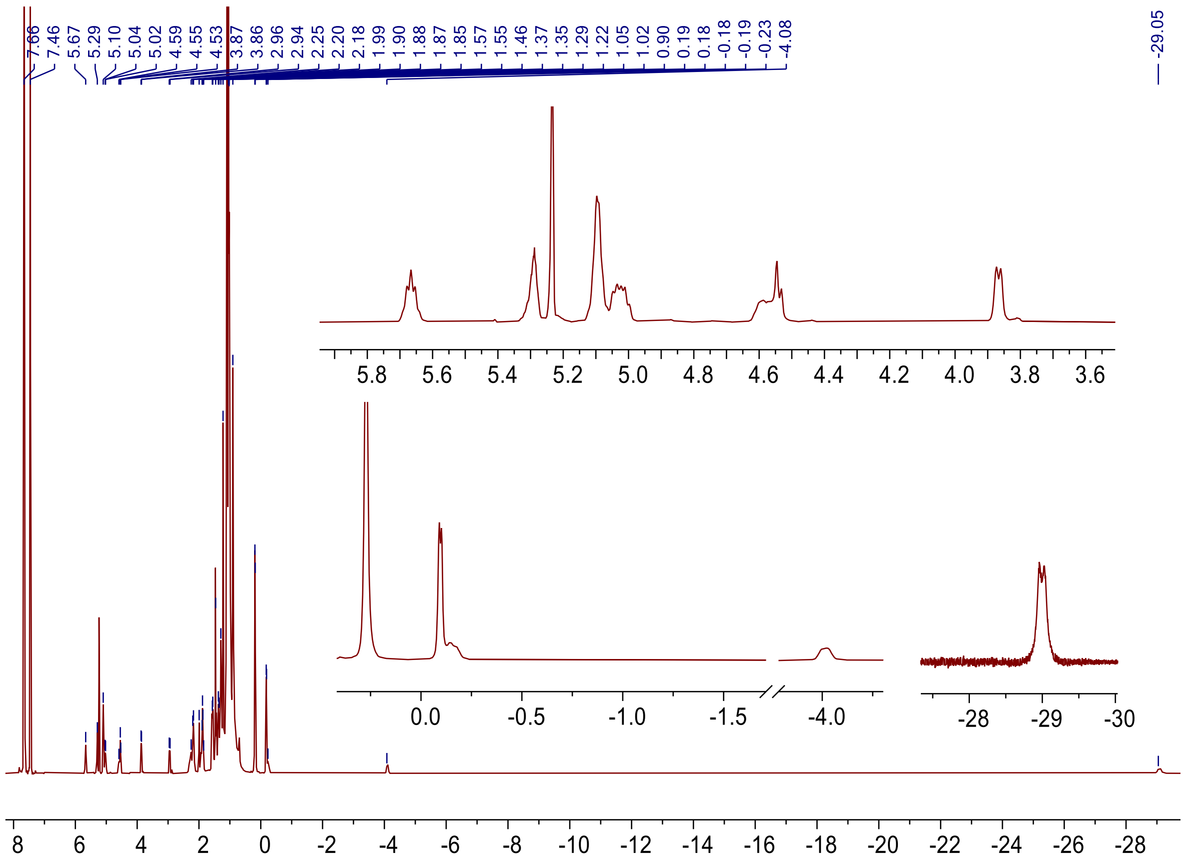

Figure S20: $^{1}H\{^{31}P\}$ (500 MHz, CD_2Cl_2, 183K) Solution NMR Spectrum after the addition of 1-butene to **[^{t}Bu-exo-NBA][BAr$^{F}_4$]** in the solid-state and dissolved into CD_2Cl_2 at 183 K.

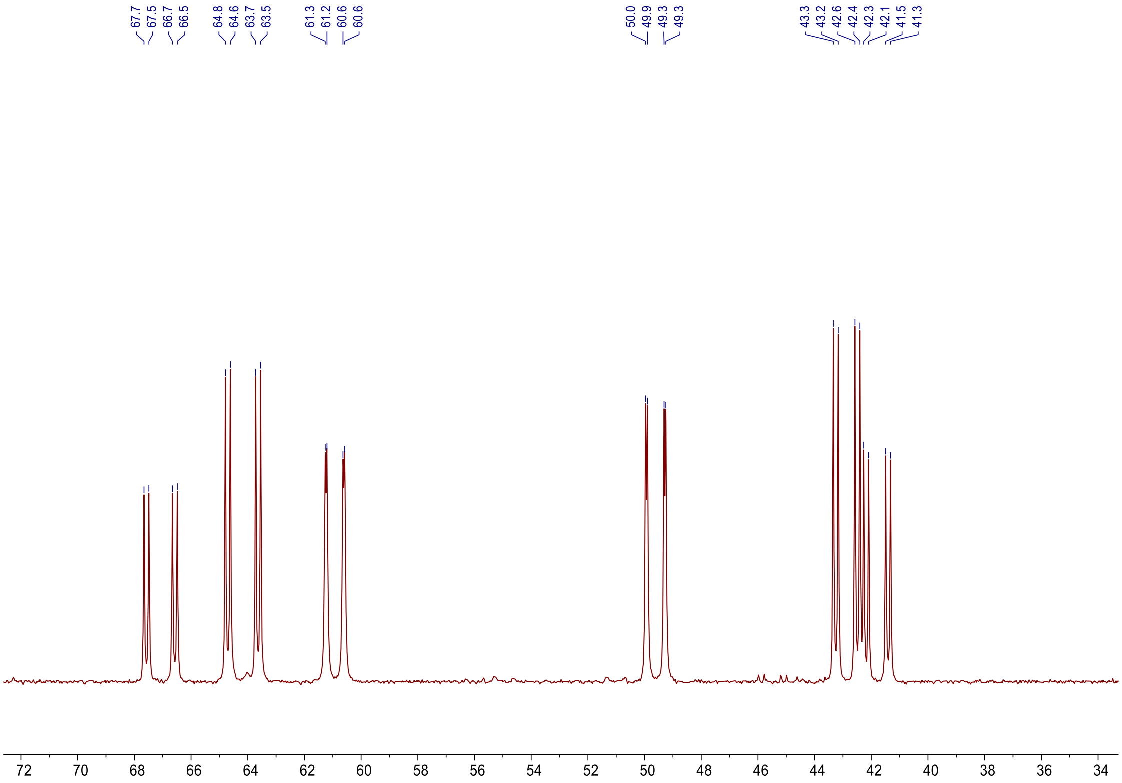

Figure S21: $^{31}P\{^{1}H\}$ (202 MHz, CD_2Cl_2, 183K) Solution NMR Spectrum after the addition of 1-butene (1 hr) to **[^{t}Bu-exo-NBA][BAr$^{F}_4$]** in the solid-state and dissolved into CD_2Cl_2 at 183 K.

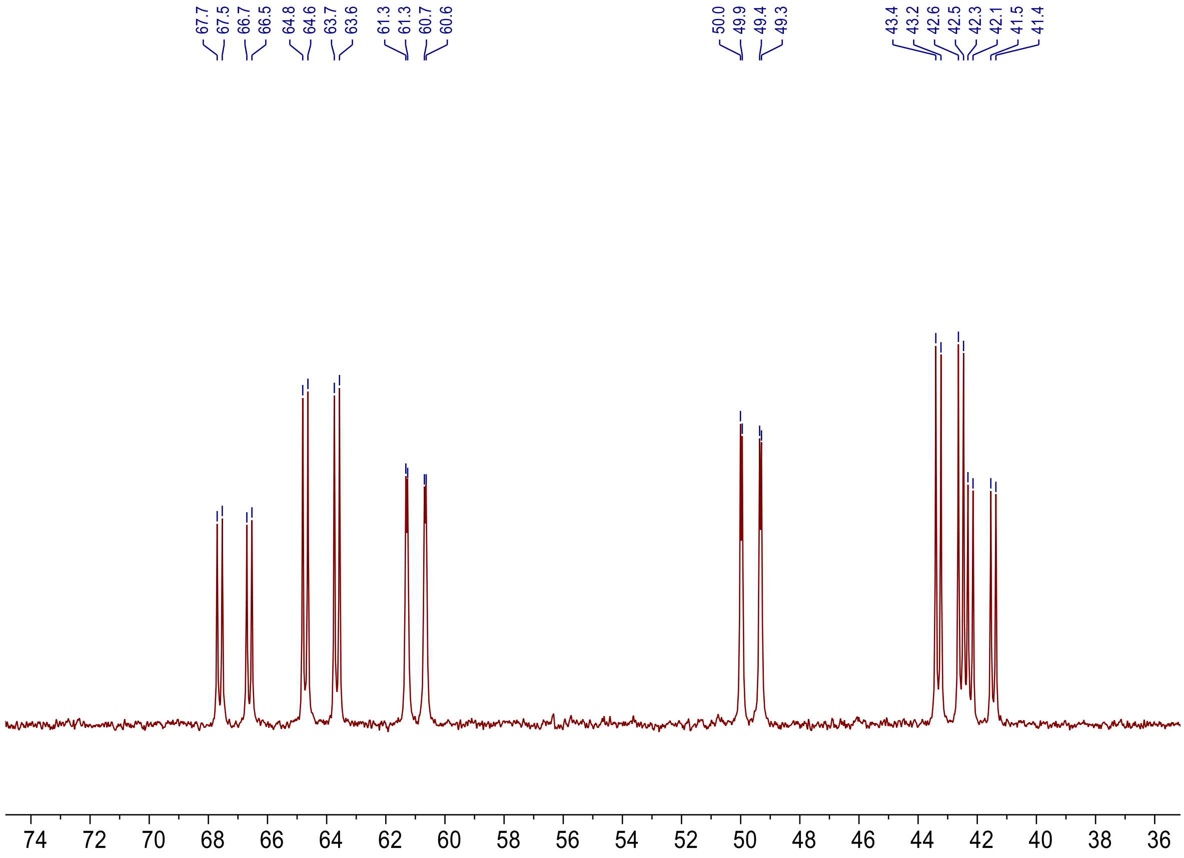

Figure S22: $^{31}P\{^{1}H\}$ (202 MHz, CD_2Cl_2, 183K) Solution NMR Spectrum after the addition and ageing under 1-butene (24 hr) to **[^{t}Bu-exo-NBA][BAr^F$_4$]** in the solid-state and dissolved into CD_2Cl_2 at 183 K.

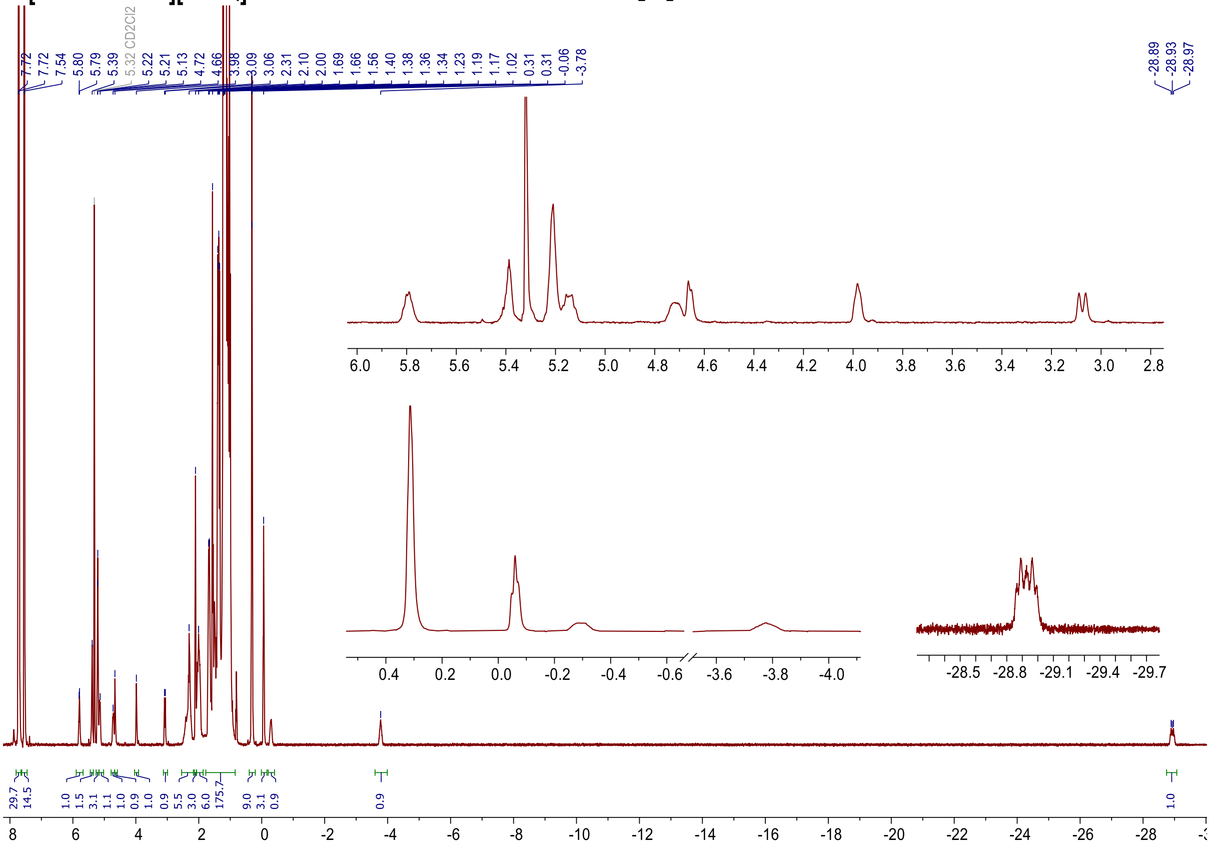

Figure S23: ^{1}H (500 MHz, CD_2Cl_2, 208 K) Solution NMR Spectrum after the addition of 1-butene to **[^{t}Bu-exo-NBA][BAr^F$_4$]** in the solid-state and dissolved into CD_2Cl_2 at 183 K and warmed to 208 K.

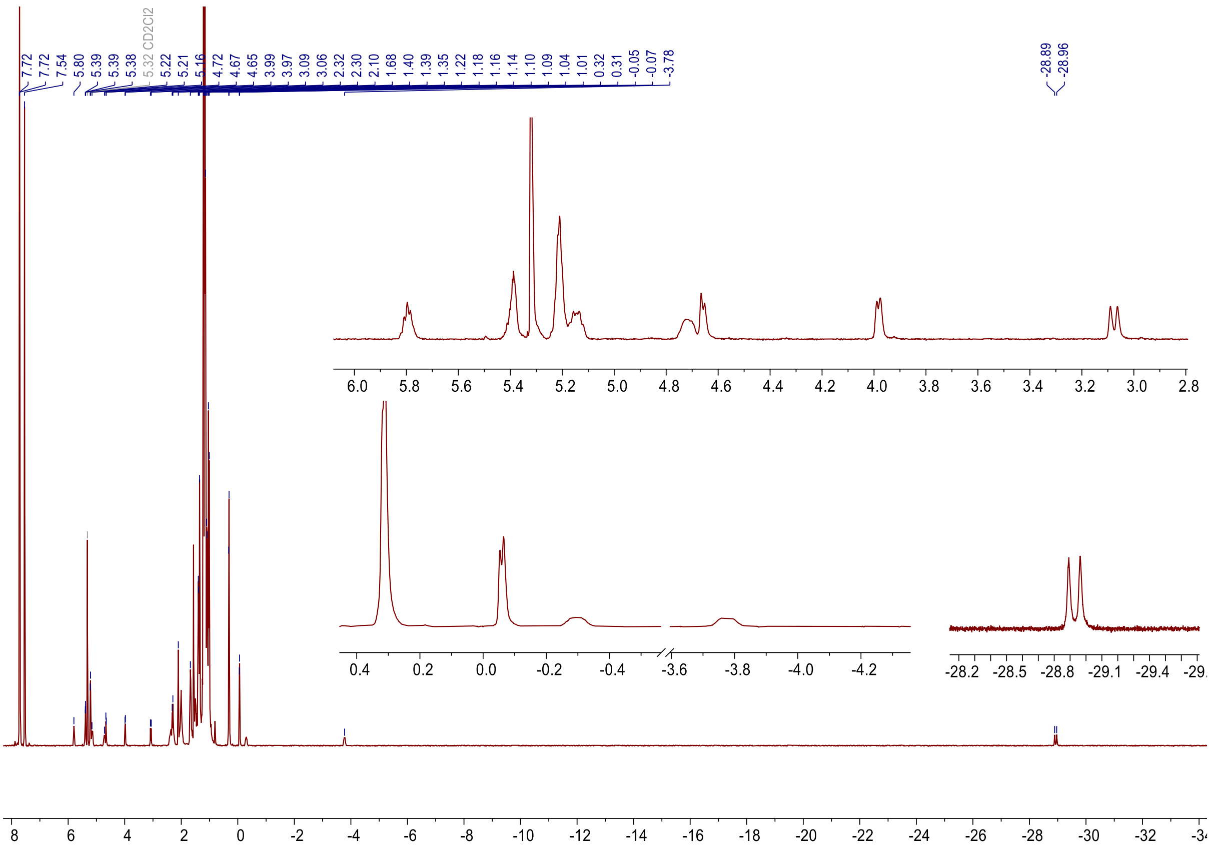

Figure S24: $^{1}H\{^{31}P\}$ (500 MHz, CD_2Cl_2, 183K) Solution NMR Spectrum after the addition of 1-butene to **[^{t}Bu-exo-NBA][BAr$^{F}_{4}$]** in the solid-state and dissolved into CD_2Cl_2 at 183 K and warmed to 208 K.

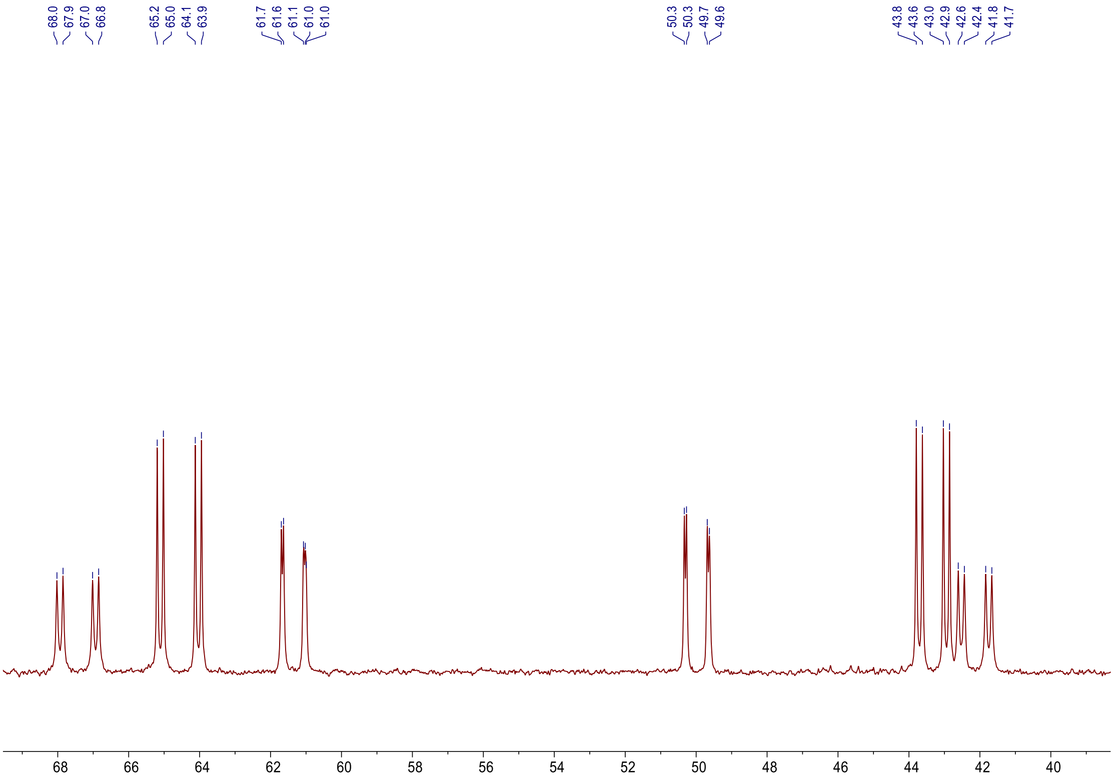

Figure S25: $^{31}P\{^{1}H\}$ (202 MHz, CD_2Cl_2, 208 K) Solution NMR Spectrum after the addition of 1-butene to **[^{t}Bu-exo-NBA][BAr$^{F}_{4}$]** in the solid-state and dissolved into CD_2Cl_2 at 183 K and warmed to 208 K.

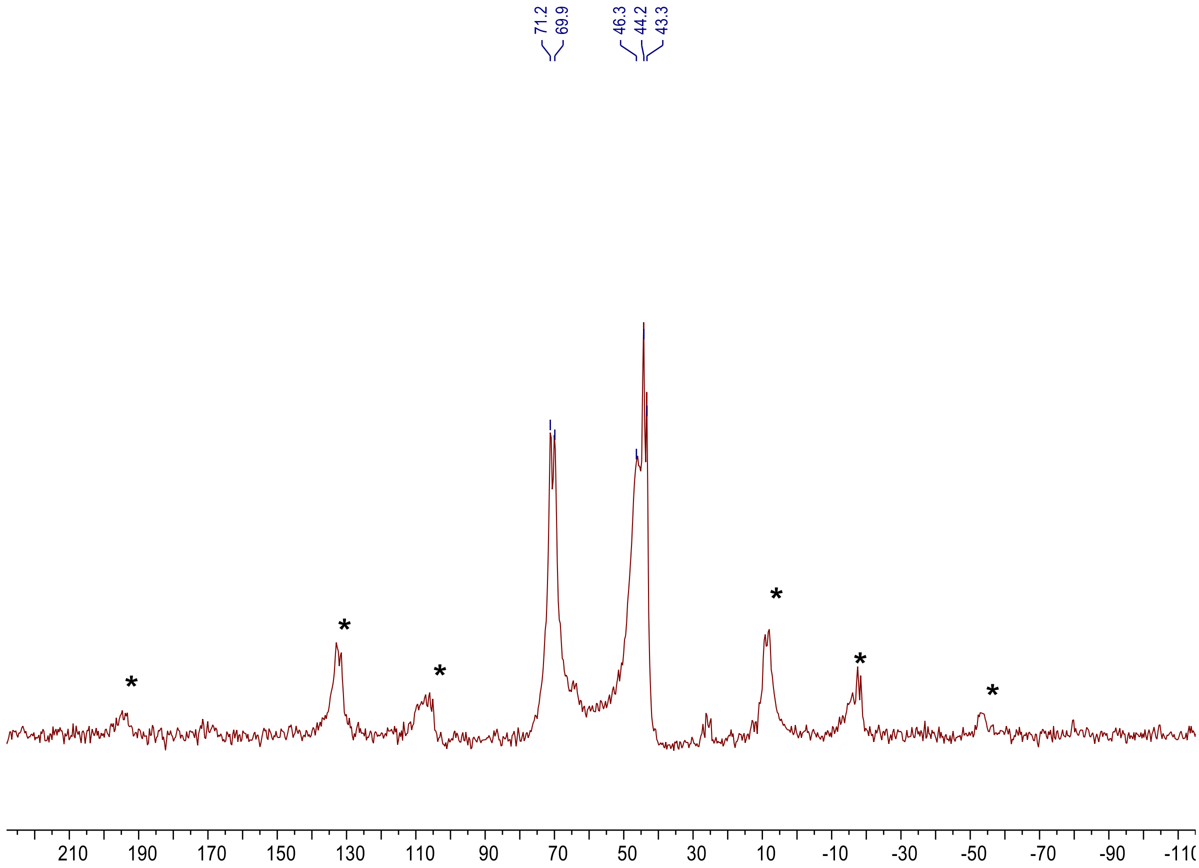

Figure S26: $^{31}P\{^{1}H\}$ (162 MHz, 10 kHz spin rate, 285 K) SSNMR Spectrum after the addition of 1-butene (1 hr) to **[^{t}Bu-exo-NBA][BAr^{F}$_4$]** in the solid-state. *Denotes spinning side bands

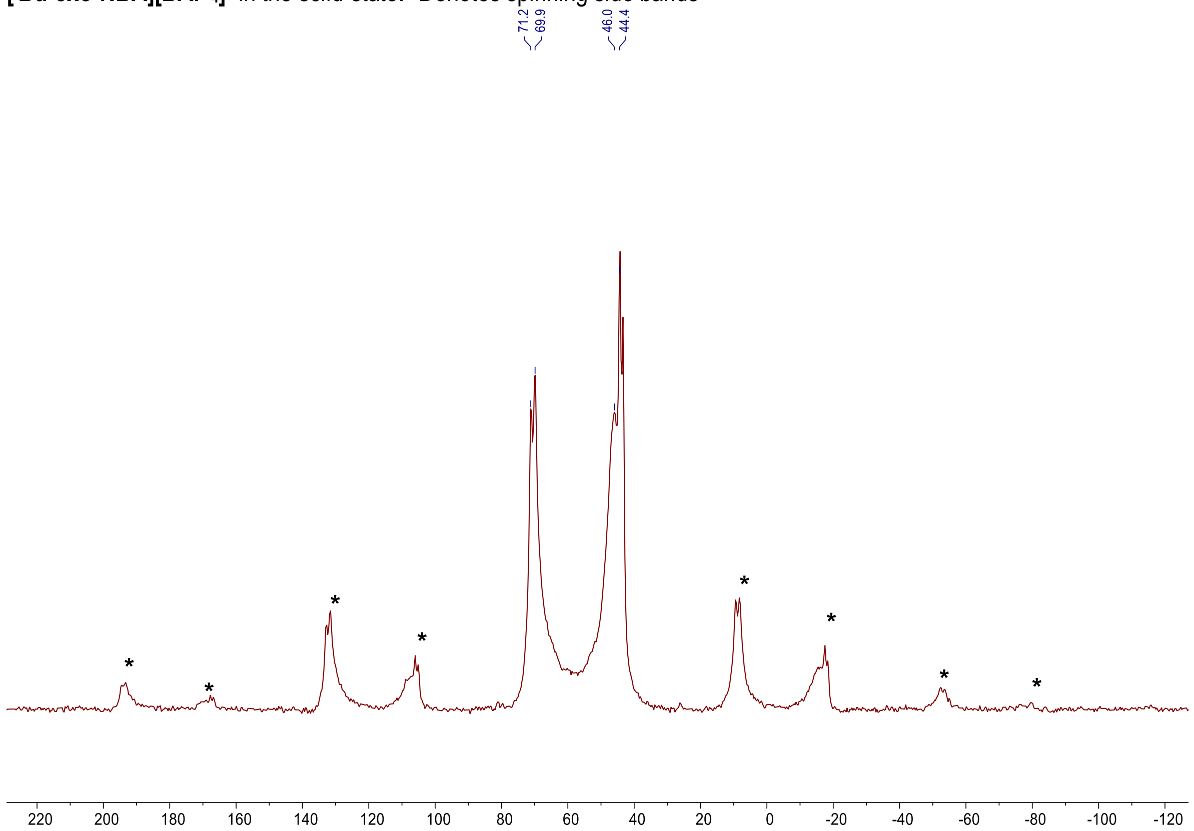

Figure S27: $^{31}P\{^{1}H\}$ (162 MHz, 10 kHz spin rate, 285 K) SSNMR Spectrum after the addition and ageing under 1-butene (24 hr) to **[^{t}Bu-exo-NBA][BAr^{F}$_4$]** in the solid-state. *Denotes spinning side bands

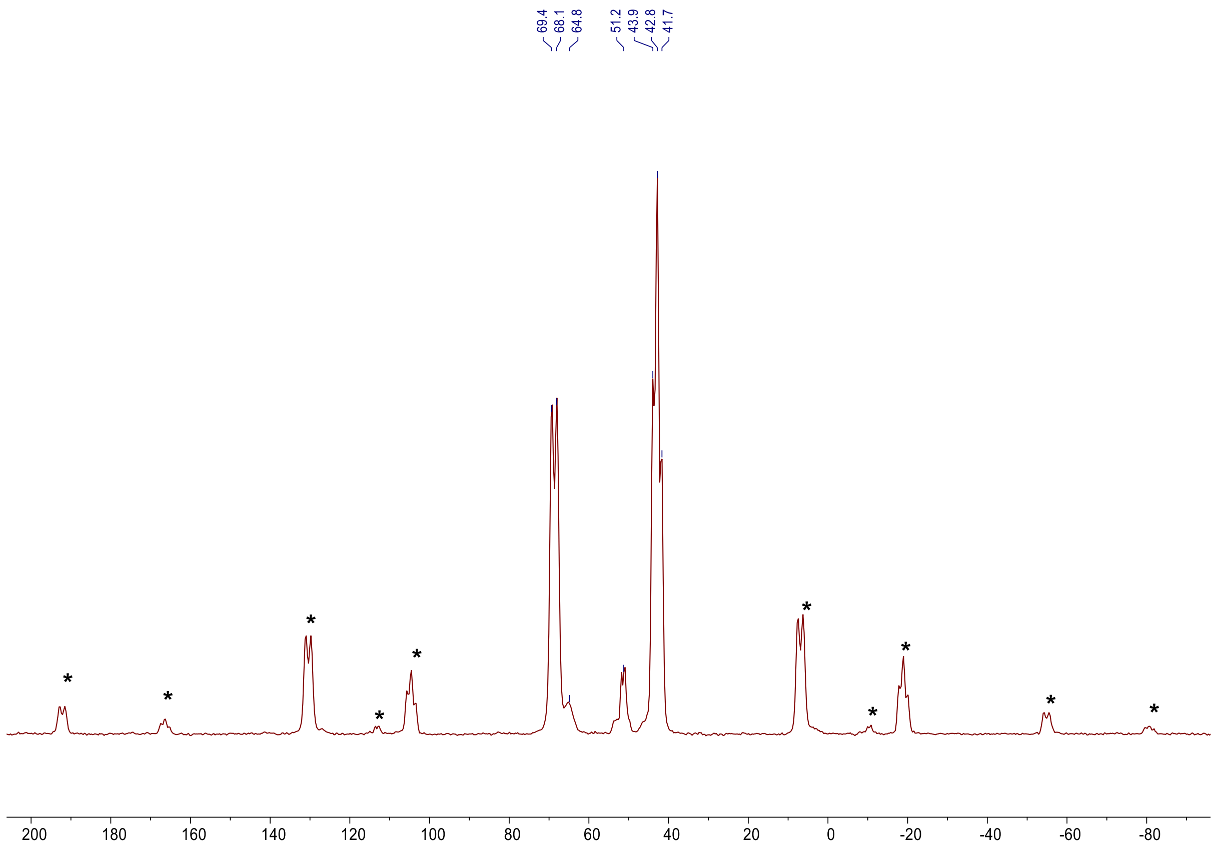

Figure S28: $^{31}P\{^1H\}$ (162 MHz, 10 kHz spin rate, 195 K) SSNMR Spectrum after the addition of 1-butene to **[^{t}Bu-exo-NBA][BAr$^F{}_4$]** in the solid-state. *Denotes spinning side bands

S4.3 Synthesis of [^{t}Bu-C_4H_6][BAr$^F{}_4$]

A finely crushed sample of **[^{t}Bu-NBD][[BAr$^F{}_4$]** (65 mg, 47 µmol) was placed into a small J. Young ampoule and placed under a hydrogen atmosphere (1 bar) for 10 mins. The hydrogen atmosphere was removed and the ampoule was cooled to -80 °C and dichloromethane (5 mL) was added, followed quickly by butadienes (15 % in hexanes, 0.3 mL, 570 µmol). The reaction mixture was stirred for 1.5 hours whilst warming to ambient temperature resulting in a deep red solution. The mixture was layered with pentane resulting in deep red crystals. Crystalline yield (57 mg, 90 %).

^{1}H Solution NMR (500 MHz, CD_2Cl_2, 298 K): δ 7.73 (8H, s, *o*-BAr$^F{}_4$), 7.57 (4H, s, *p*-BAr$^F{}_4$), 5.54 (2H, m, =C*H*), 4.47 (2H, m, =C*H*), 2.35 (1H, m, CH_2), 2.19 (2H, m, CH_2), 2.11 (2H, m, =C*H*), 1.87 (1H, m, CH_2), 1.49, (2H, m, CH_2,), 1.40 (18H, d, PC(CH_3)$_3$), 1.16 (18H, d, PC(CH_3)$_3$).

$^{31}P\{^1H\}$ Solution NMR (162 MHz, CD_2Cl_2, 298 K): δ 36.1 (d, J_{Rh-P} = 165 Hz)

$^{13}C\{^1H\}$ solution NMR (125 MHz, CD_2Cl_2, 298 K): δ 161.7 (q, $^1J_{C-B}$ = 49 Hz, *ipso*-BAr$^F{}_4$), 134.7 (s, *p*-BAr$^F{}_4$), 128.8 (br q, $^2J_{F-C}$ = 32, *o*-BAr$^F{}_4$), 124.6 (q, $^1J_{F-C}$ = 272.0 Hz, -CF_3), 117.5 (br d, *m*-BAr$^F{}_4$), 96.3 (d, J = 6 Hz, CH_2=CH), 55.5 (br, CH_2=), 38.9 (m, P*C*(CH_3)$_3$), 31.4 (s, PC(*C*H_3)$_3$), 29.3 (s, PC(*C*H_3)$_3$), 22.2 (br s, CH_2), 20.5 (t, J_{P-C} = 12 Hz, CH_2).

$^{31}P\{^1H\}$ SSNMR (162 MHz, 10 kHz spin rate, 285 K): δ 40.1 (br s)

$^{13}C\{^1H\}$ SSNMR (101 MHz, 10 kHz spin rate, 285 K): δ 166.0, 163.6, 136.5, 135.0, 131.5, 130.9, 125.3, 117.9 (BAr$^F{}_4$), 96.7, 56.1 (C_4H_6), 39.4, 37.1, 35.5, 31.7, 29.6, 23.0 (phosphine).

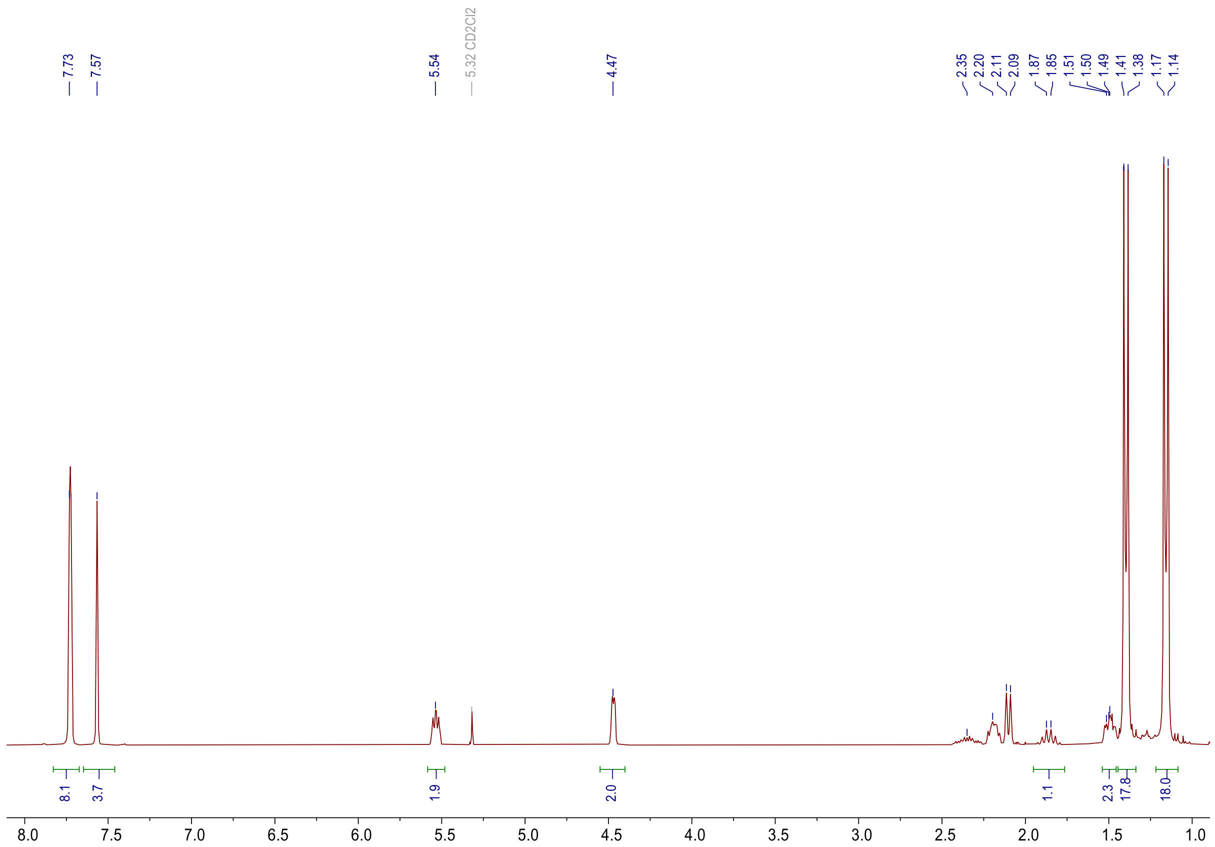

Figure S29: ^{1}H (500 MHz, CD_2Cl_2, 298 K) Solution NMR Spectrum of **[^{t}Bu-C_4H_6][BAr^F_4]**

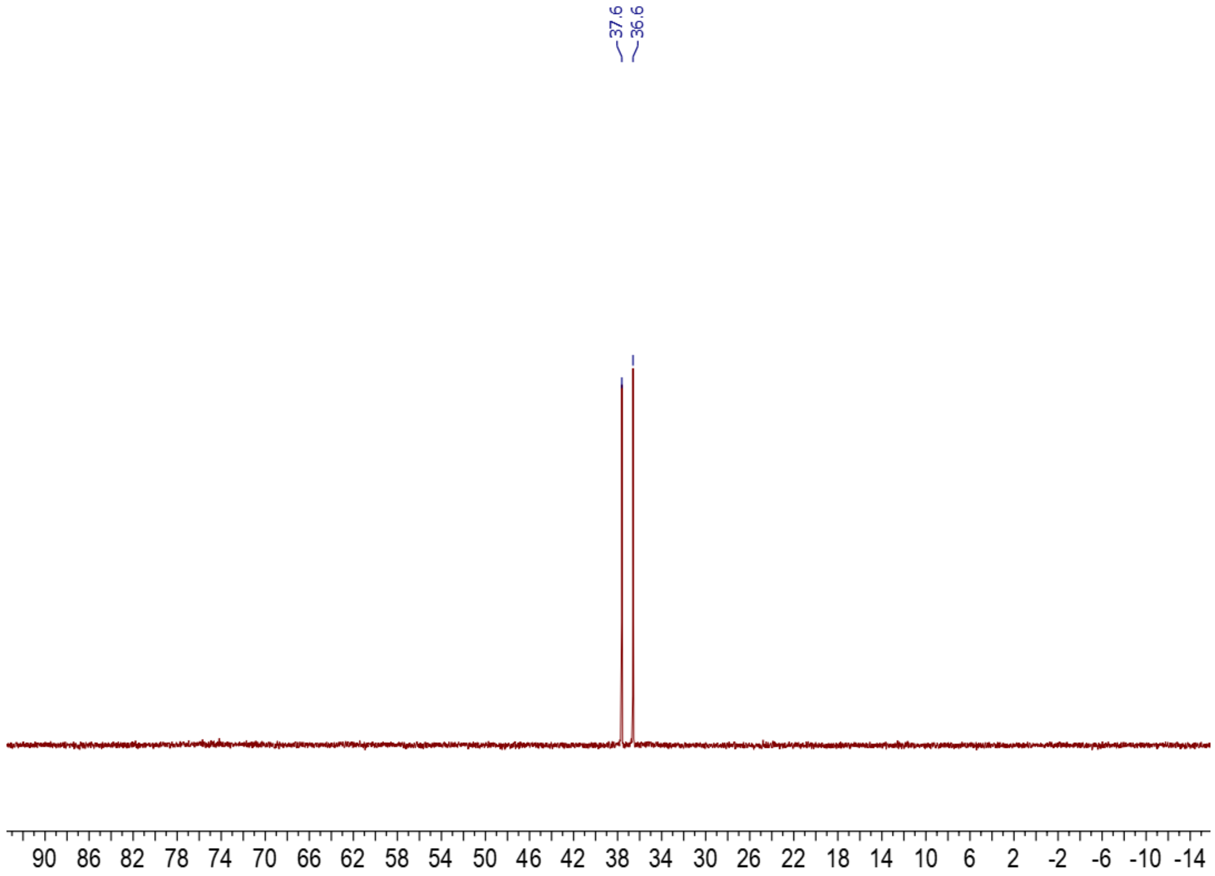

Figure S30: $^{31}P\{^{1}H\}$ (162 MHz, CD_2Cl_2, 298 K) Solution NMR Spectrum of **[^{t}Bu-C_4H_6][BAr^F_4]**

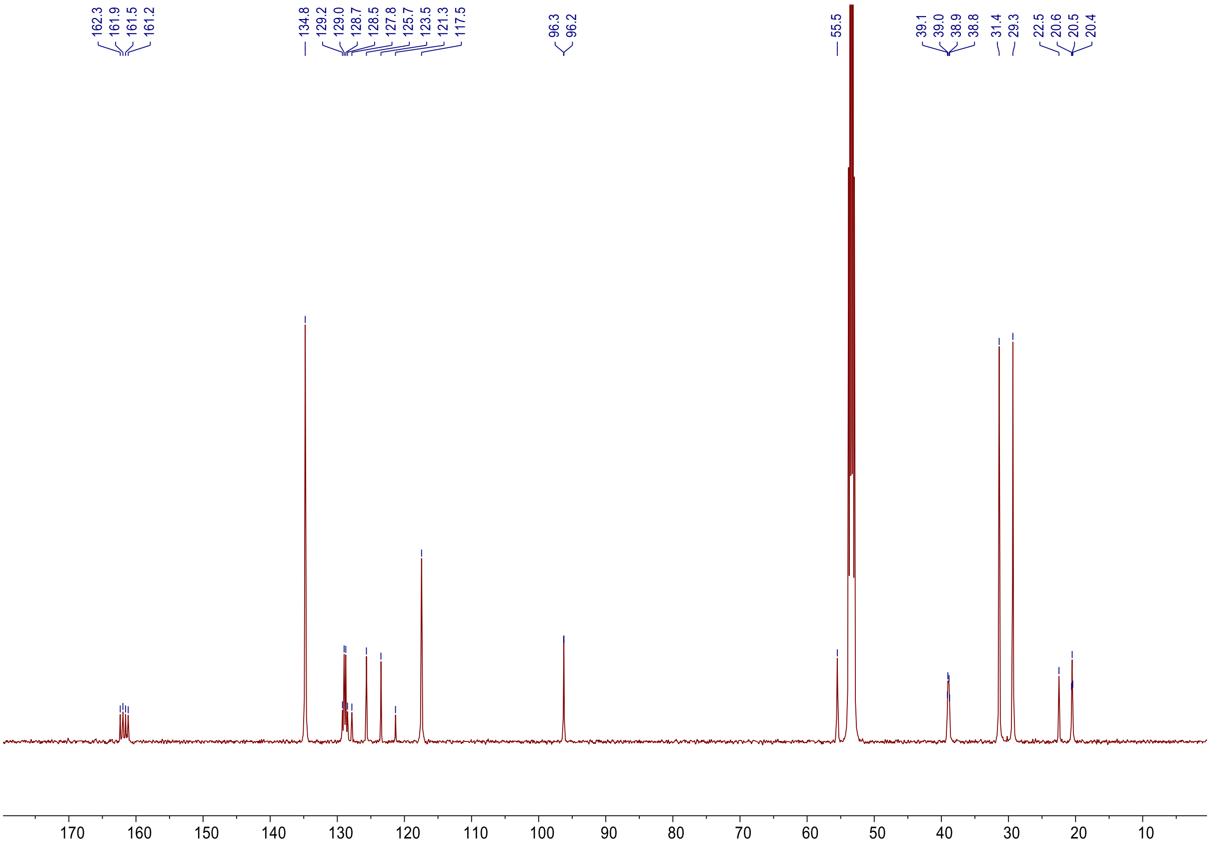

Figure S31: $^{13}C\{^{1}H\}$ (125 MHz, CD_2Cl_2, 298 K) Solution NMR Spectrum of **[^{t}Bu-C_4H_6][BAr$^{F}_{4}$]**

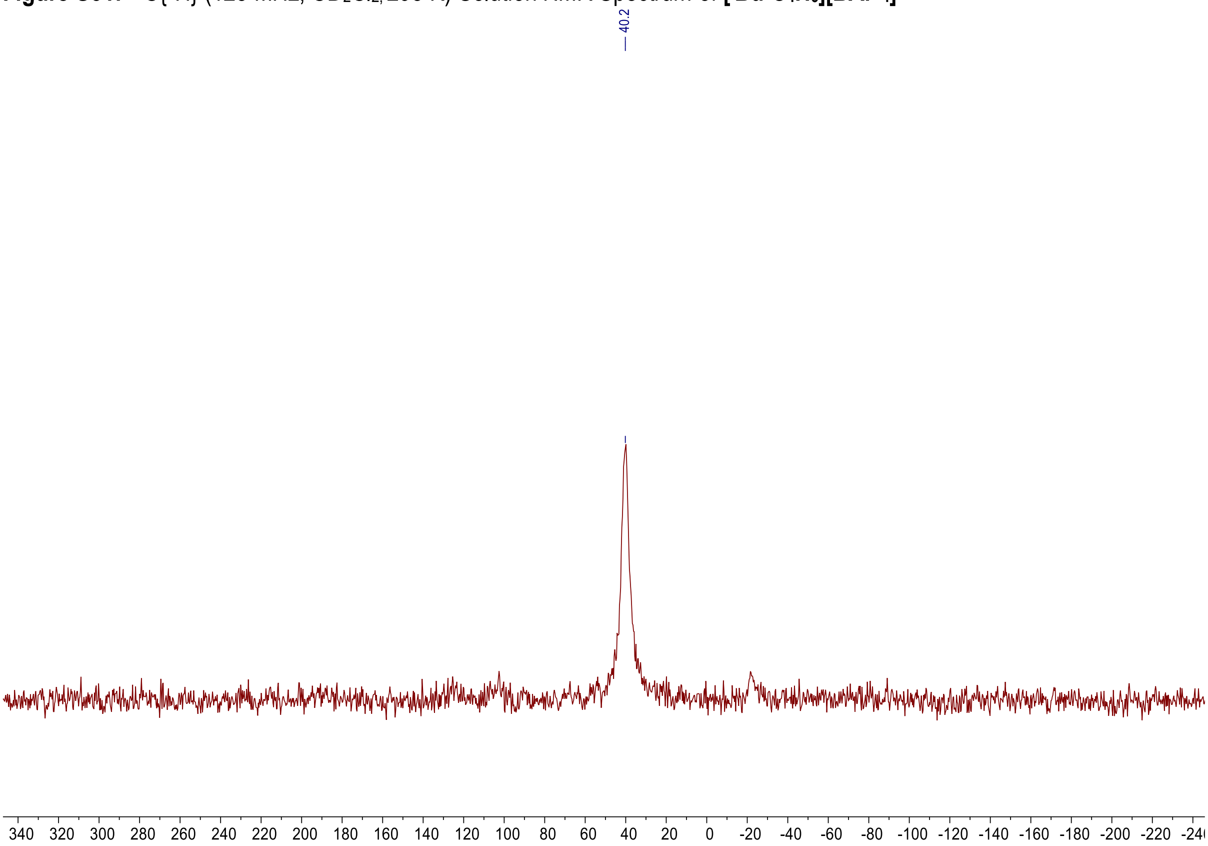

Figure S32: $^{31}P\{^{1}H\}$ (162 MHz, 10 kHz spin rate, 285 K) SSNMR Spectrum of **[^{t}Bu-C_4H_6][BAr$^{F}_{4}$].**

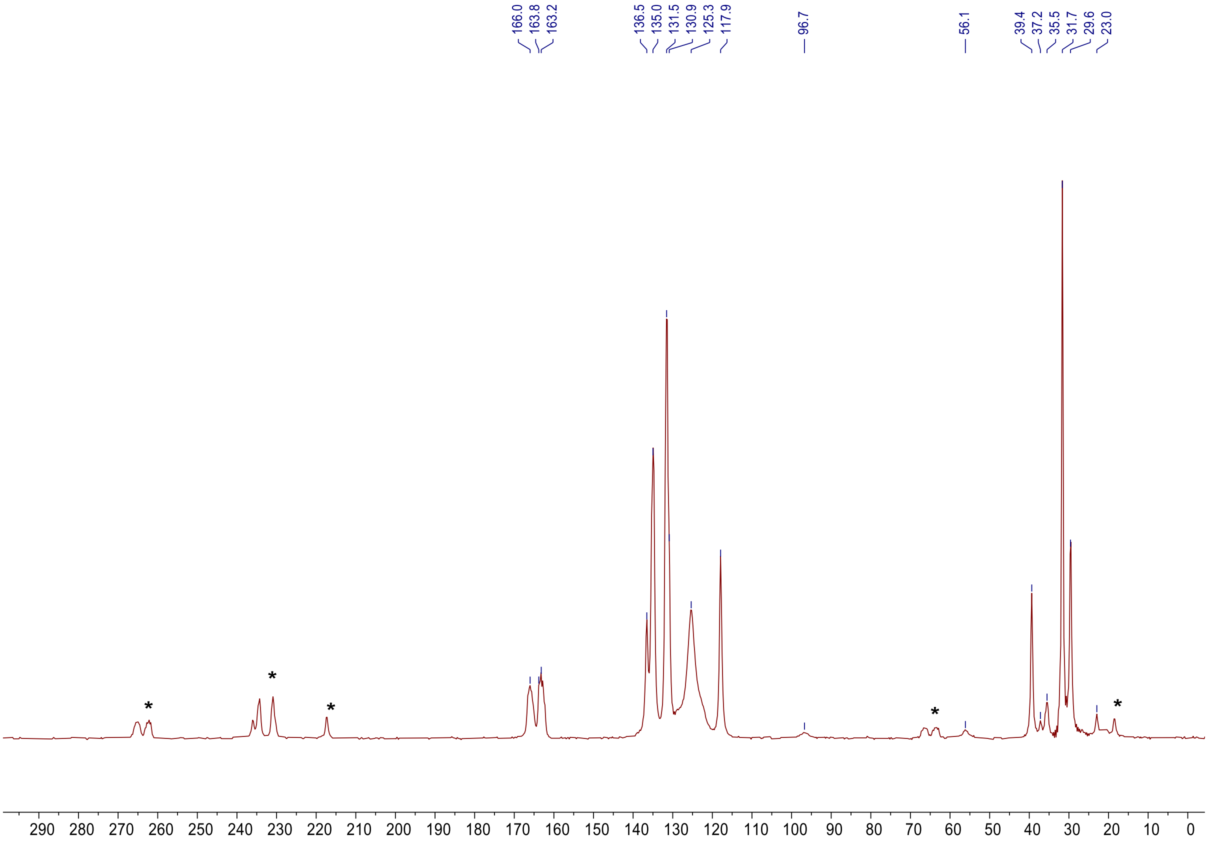

Figure S33: $^{13}C\{^1H\}$ (100 MHz, 10 kHz spin rate, 285 K) SSNMR Spectrum of **[^tBu-C_4H_6][BAr$^F{}_4$]**. *Denotes spinning side band

S5 Anion Microenvironment Comparisons

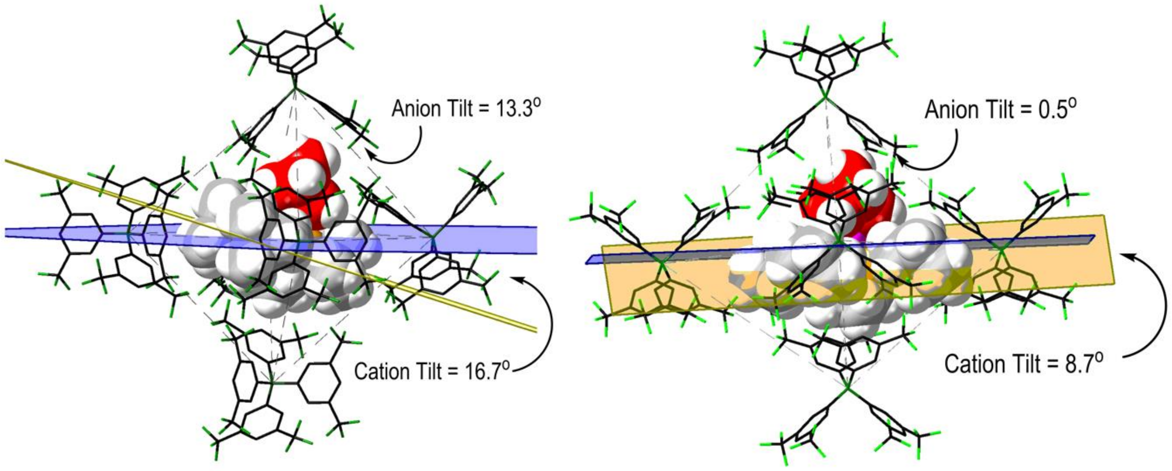

Figure S34: Comparison of anion cage and metal cation orientations in **[^tBu-NBD][BAr$^F{}_4$]** (left) and **[Cy-NBD][BAr$^F{}_4$]** (right). Cation shown at van der Waals radii. Cation tilt is the angle between the planes of the equatorial boron atoms in the $[BAr^F{}_4]^-$ anions and the phosphine ligand coordinated to the rhodium cation. The anion tilt is the degree of which the para carbon of the aryl ring closest to the NBD/NBA fragment, deviates from the angle between the boron atoms atom of the $[BAr^F{}_4]^-$ anion

S6 Steric Topography

%V_{BUR} measurements of the phosphine ligands were calculated using SambVca 2[16] in the octahedral microenvironment with the following settings: mesh spacing 0.1 Å, atomic radii 1.17xBondi radii, hydrogen atoms included in calculations. %V_{BUR} values were calculated for a range sphere of 3.5 Å, steric maps generated by the SambVca program.[16]

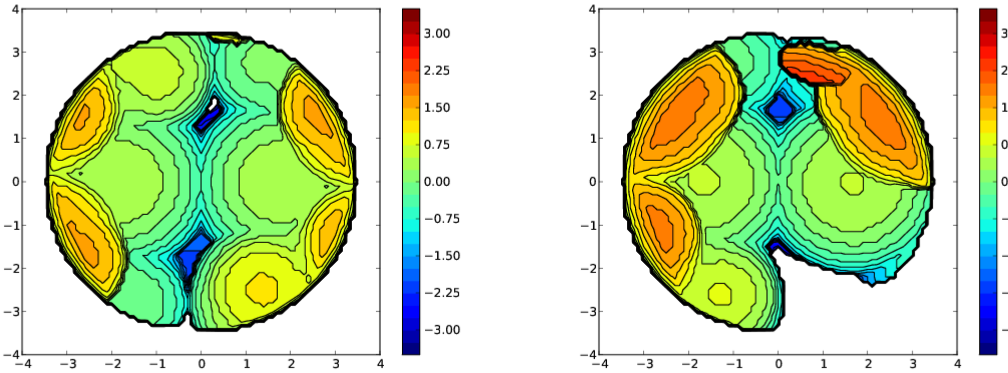

Figure S35: Steric Topography Diagram of **[^{t}Bu-NBD][BAr^F$_4$]** (left) and**[^{t}Bu-exo-NBA][BAr^F$_4$]** (right). %V_{BUR} calculated as 58.1 % for **[^{t}Bu-NBD][BAr^F$_4$]** and as 57.9 % for **[^{t}Bu-exo-NBA][BAr^F$_4$].**

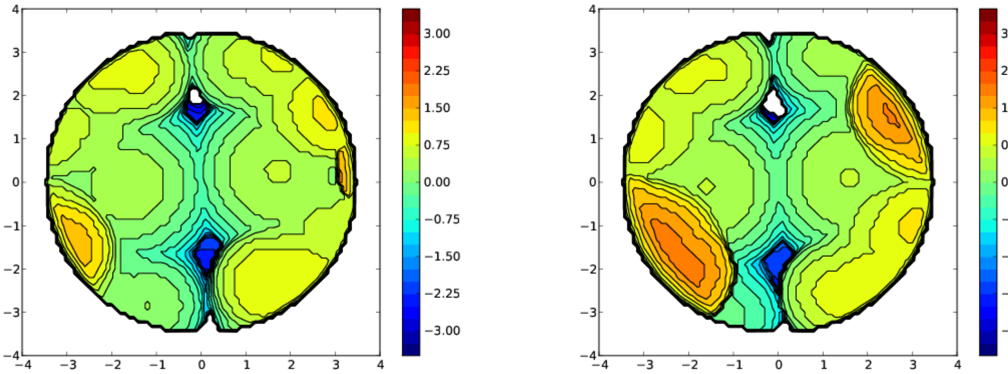

Figure S36: Steric Topography Diagram of **[Cy-NBD][BAr^F$_4$]** (left) and **[Cy-*endo*-NBA][BAr^F$_4$]** (right). %V_{BUR} calculated as 56.9 % for **[Cy-NBD][BAr^F$_4$]** and as 60.4 % for **[Cy-*endo*-NBA][BAr^F$_4$].**

S7 Volume Cavity Calculations

Cavity volumes were calculated after removal of NBD or NBA fragments as solvent-accessible voids using the Olex2 software program (probe radius = 1.41 Å; grid = 0.2 Å), Van der Waal radii taken from Alvarez[17] and the corresponding graphics were produced using the CrystalMaker software program.

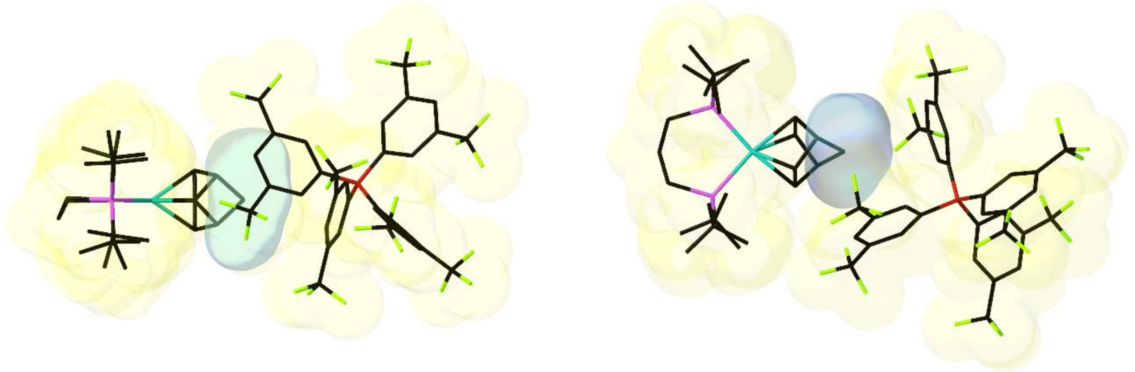

Figure S37: Cavity volumes for **[^{t}Bu-NBD][BAr^F$_4$]** shown in blue; Van der Waal surface volumes shown in yellow (NBD omitted). Cavity volumes calculated as 62 Å^3 for **[^{t}Bu-NBD][BAr^F$_4$].**

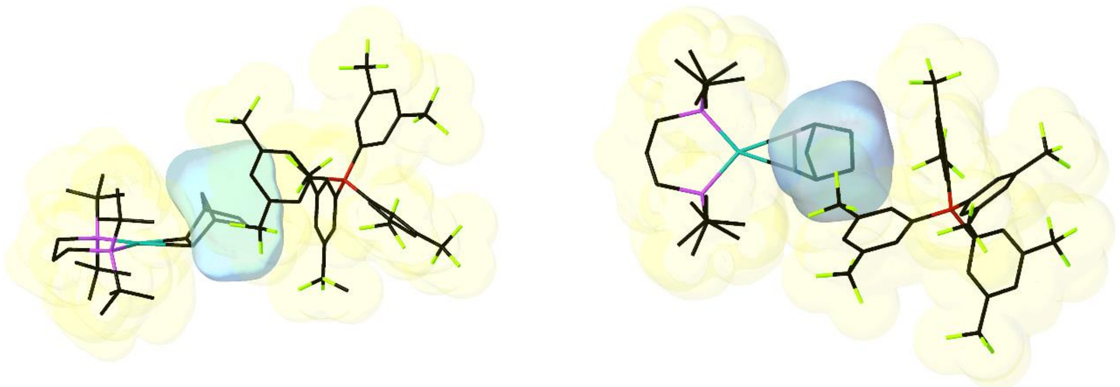

Figure S38: Cavity volumes for **[^{t}Bu-exo-NBA][BAr$^{F}_{4}$]** shown in blue; Van der Waal surface volumes shown in yellow (NBA omitted). Cavity volumes calculated as 108 Å^{3} for **[^{t}Bu-exo-NBD][BAr$^{F}_{4}$].**

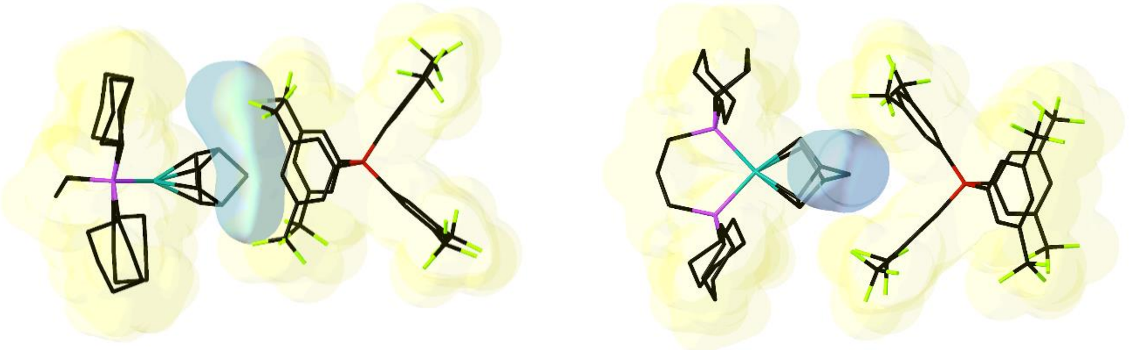

Figure S39: Cavity volumes for **[Cy-NBD][BAr$^{F}_{4}$]** shown in blue; Van der Waal surface volumes shown in yellow (NBD omitted). Cavity volumes calculated as 65 Å^{3} for **[Cy-NBD][BAr$^{F}_{4}$].**

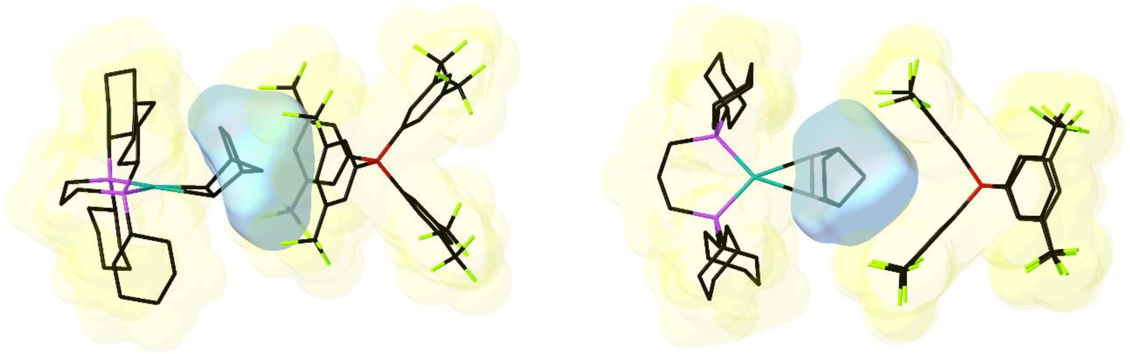

Figure S40: Cavity volumes for **[Cy-*endo*-NBA][BAr$^{F}_{4}$]** shown in blue; Van der Waal surface volumes shown in yellow (NBA omitted). Cavity volumes calculated as 119 Å^{3} for **[Cy-*endo*-NBD][BAr$^{F}_{4}$].**

S8 Computational Methods

S8.1 Solid State Calculations

All static Kohn-Sham DFT calculations were performed on periodic models of the studied rhodium complexes, employing the Gaussian Plane Wave (GPW) formalism as implemented in the QUICKSTEP[18] module within the CP2K program suite (Version 5.0).[19] Molecularly optimized basis sets of double-ζ quality plus polarization in their short-range variant (DZVP-MOLOPT-SR-GTH)[20] were used on all atomic species. The interaction between the core electrons and the valence shell (Rh: 17, B: 3, C: 4, P: 5, Cl: 7, F: 7, H: 1 electrons) was described by Goedecker-Teter-Hutter (GTH) pseudo potentials.[21-23] The generalized gradient approximation (GGA) to the exchange-correlation functional according to Perdew-Burke-Ernzerhof (PBE)[24] was used in combination with Grimme's D3-correction for dispersion interactions.[25] The auxiliary plane wave basis set was truncated at a cutoff of 500 Ry. The maximum force convergence criterion was set to 10^{-4} Eh·Bohr^{-1}, whilst default values were used for the remaining criteria. The convergence criterion for the self-consistent field (SCF) accuracy was set to 10^{-7} Eh and 10^{-8} Eh for geometry optimizations and vibrational analysis, respectively.

The Brillouin zone was sampled using the Γ-point. Initial coordinates for **[Cy-*endo*-NBA][BAr$^F_4$]** and **[^{t}Bu-*exo*-NBA][BAr$^F_4$]** were obtained from the experimental crystallographic data, with the hydrogen positions normalised where possible with Mercury.[45] Periodic boundary conditions (PBC) were applied throughout in combination with fixed unit cell parameters obtained from experiment. All geometries were first partially relaxed, keeping the heavy atoms (non-H, F) fixed, then fully relaxed without imposing any constraints, whilst keeping unit cell parameters constant in both cases.

For reactivity studies different reaction pathways were initially explored using an isolated rhodium molecular cation model with the Gaussian suite of programs (see details below). Transition states located in this way provided the basis for transition state searches in the solid state, with pre-optimisations in the solid state run by fixing the key reacting atoms at one of the Rh-centres. A partial vibrational analysis was then used to identify the corresponding imaginary mode. This pre-optimized TS structure was then refined using the dimer method[26] with the tighter convergence criteria detailed above. For challenging fluxional processes the climbing image nudged elastic band (CI-NEB) method,[27] using 8 images, was used to obtain candidate transition states that were then optimised using the dimer method as above. All optimized stationary points were characterized by analysis of their numerical second derivatives with a displacement of 0.01 Bohr. Minima and transition states have no or exactly one imaginary eigenvalue, respectively. All transition states were further analysed using an "IRC-like" approach, whereby transition state geometries were displaced along the negative mode in both directions and then fully optimising the two resulting structures. Further details on this protocol have been reported elsewhere.[28-30]

For the crystal structures of both **[Cy-NBA][BAr$^F_4$]** and **[^{t}Bu-NBA][BAr$^F_4$]** Z = 4 and so in principle four different Rh centres could be chosen as the reacting site. Test calculations showed the energy of the *exo-endo* rearrangement was independent of the Rh centre employed (see Table S1).

Gibbs free energies for structures computed in the solid state were calculated using the TAMkin software toolkit.[31]

CIF-files of the fully optimised systems, generated using VESTA,[46] were analysed using the Crystal Explorer package,[47] using a central cation and the six nearest neighbour anions.

The cation with its six neighbouring anions where generated the following method:

- Open the CIF-file in CrystalExplorer.
- Select the cation of interest, Select Display -> Invert Selection and then Remove -> Selected Atoms.
- Select Actions - > Generate Atoms Within Radius.
- Enter an appropriate radius, (around 11 Å from the central cation in our case).
- Select the surrounding anions and the central cation and then select Remove -> "Incomplete Fragments".

- Select all remaining atoms and then Actions-> “Complete Fragments”.
- Select the central cation then Actions -> “Generate Surface” to generate the Hirshfeld Surface.
- Modify the charges and multiplicities of all the fragments as required.
- Choose the “Fast (HF/3-21G)” method when calculating the interaction energies.

All computed structures are available as a separate file of Cartesian coordinates.

S8.2 Molecular Calculations

Molecular calculations employed the GAUSSIAN 09 (revision D.01) program package[32] and employed the BP86 GGA functional.[33-34] Stuttgart-Dresden (SDD)[35] relativistic effective core potentials (ECP) in combination with the associated basis sets were utilized to describe Rh and P, with polarization functions added for P (ζ = 0.387).[36] The 6-31G(d,p) basis sets[37-38] were used on remaining atoms. Energies were then recomputed with the def2-TZVP basis set[48-49] on all atoms, with an ECP on Rh. These included a D3 dispersion correction for the *exo*- and *endo*-bound isomers of **[Cy-NBA]$^+$** and **[^{t}Bu-NBA]$^+$** while for the isomers of [Rh(dtbpp)(C_4H_8)]$^+$ a PCM[50] correction for dichloromethane solvent was also included. The resultant SCF energies were combined with the thermodynamic corrections from the original ‘gas-phase’ optimisations to provide the free energies quoted in the text. Each isomer of [Rh(dtbpp)(C_4H_8)]$^+$ was subjected to a rigorous conformational searching using our published protocol.[51]

S8.3 Electronic Structure Analyses

Electronic structure analyses were performed on the geometries of the Rh cations extracted from the CP2K-optimised structures with the heavy atoms fixed at the experimental positions. An electron density file suitable for further analysis was generated from a single-point calculation.

The topology of the electron density was analysed by means of QTAIM (Quantum Theory of Atoms in Molecules),[39] as implemented in the AIMALL package.[40] Inner shell electrons on Rh and P modelled by ECPs were represented by core density functions (extended wavefunction format).

NBO calculations were performed using the NBO 6.0 program,[41] using the same geometries as for the QTAIM calculations above.

NCI calculations were performed using the NCIPLOT program,[42-43] using the nearest neighbour ion-pair molecular structures extracted from CP2K optimised geometries. The promolecular electron density was employed and the results displayed with VMD.[52]

Orbital plots were created with Chemcraft[44] with an outer contour value of 0.0625.

S8.4 Solution-phase NMR Calculations on the Isomers of [Rh(dtbpp)(C_4H_8)]$^+$

NMR calculations were performed within the GIAO framework using ADF 2019.3[53-54] with the hybrid PBE0 functional[55] and Slater-type basis sets of triple-ζ (TZP) quality.[56] Relativistic effects were treated by the 2-component zeroth-order regular approximation (ZORA).[57-58] Computed chemical shifts are quoted relative to TMS (δ^{iso}_{calc} = 31.16 ppm).

S9 Computed Reaction Profiles

S9.1 NBA rearrangement processes in the solid state

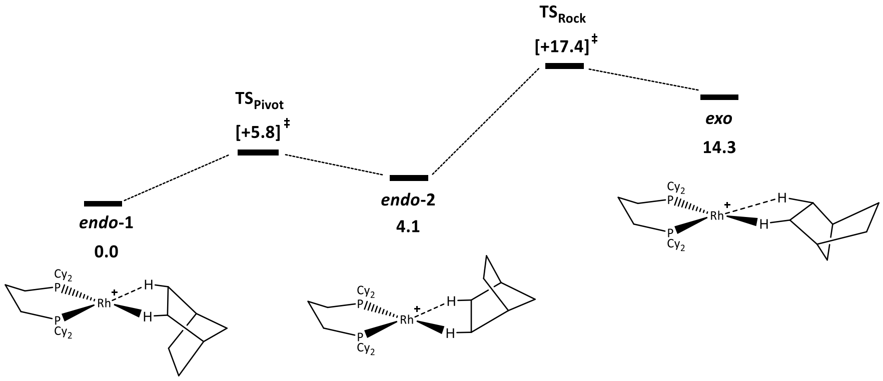

Figure S41: Computed free energy reaction profile (kcal/mol) for the pivot and rock rearrangements of **[Cy-*endo*-NBA][BAr^F$_4$]** system. Only the reacting Rh complex is shown with [BAr^F$_4$]$^-$ anions and spectator Rh cations omitted for clarity.

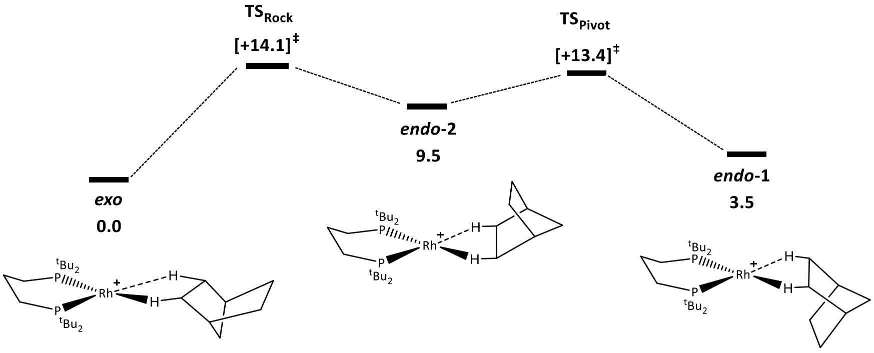

Figure S42: Computed free energy reaction profile (kcal/mol) for the rock and pivot rearrangements of the **[^{t}Bu-*exo*-NBA][BAr^F$_4$]** system. Only the reacting Rh complex is shown with [BAr^F$_4$]$^-$ anions and spectator Rh cations omitted for clarity.

S9.2 Different NBA rearrangements in the solid state

Table S1: SCF Energy differences for the four different NBA rearrangements in the solid state, compared with the fully optimised experimental structures

System	ΔE (kcal / mol)
Cy-all-endo-NBA	0.00
Cy-one-exo-NBA Alt A	14.02
Cy-one-exo-NBA Alt B	13.85
Cy-one-exo-NBA Alt C	13.93
Cy-one-exo-NBA Alt D	14.60
**System**	**ΔE (kcal / mol)**
^{t}Bu-all-exo-NBA	0.00
^{t}Bu-one-endo-NBA Alt A	2.40
^{t}Bu-one-endo-NBA Alt B	2.28
^{t}Bu-one-endo-NBA Alt C	2.40
^{t}Bu-one-endo-NBA Alt D	2.42

S10 Electronic Structure Analyses

S10.1 [Cy-*endo*-NBA][BAr$^F_4$]

Table S2: Key bond distances (Å) for fully optimised and heavy atoms fixed geometries, compared with experimental data.

Distance (Å)	Fully Optimised	Heavy Atom Fixed[a]	Experiment
Rh – P1	2.217	2.207	2.2065(4)
Rh – P2	2.213	2.205	2.2049(4)
Rh – H11	1.886	1.889	1.89(3)
Rh – H21	1.891	1.879	1.89(3)
Rh – H31	2.705	2.820	2.881(4)
Rh – C1	2.416	2.408	2.408(2)
Rh – C2	2.423	2.402	2.4022(19)
C1 – H11	1.151	1.149	1.08(3)
C1 – H12	1.096	1.096	0.99(3)
C2 – H21	1.149	1.151	1.05(3)
C2 – H22	1.096	1.097	0.96(3)
C3 – H31	1.102	1.102	0.99(3)
C3 – H32	1.100	1.099	0.99(3)

[a] data are based on the experimental structure with only the positions of H and F atoms being optimised.

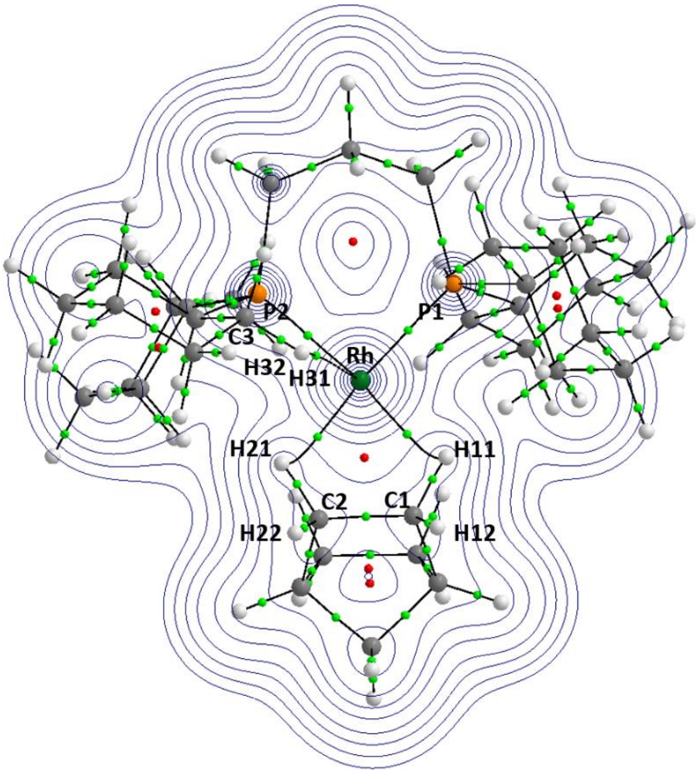

Figure S43: QTAIM molecular graph for heavy atom fixed **[Cy-*endo*-NBA]$^+$** with density contours plotted in the Rh-H11-H21 plane. Bond critical points (BCPs) are indicated in green and ring critical points (RCPs) are indicated in red.

Table S3: Key BCP metrics.

Bond	$\rho\ (r)$ /e bohr^{-3}	$\nabla^2\ \rho\ (r)$ /e bohr^{-5}	H (r)
Rh – P1	0.119	0.076	-0.059
Rh – P2	0.119	0.074	-0.059
Rh – H11	0.059	0.246	-0.007
Rh – H21	0.060	0.249	-0.008
Rh – H31	0.013	0.036	0.001
C1 – H11	0.236	-0.632	-0.207
C1 – H12	0.277	-0.950	-0.280
C2 – H21	0.234	-0.624	-0.206
C2 – H22	0.277	-0.945	-0.279
C3 – H31	0.272	-0.902	-0.270
C3 – H32	0.272	-0.896	-0.271

Table S4: Key donor-acceptor interactions for **[Cy-*endo*-NBA][BAr^F$_4$]** from the NBO second order perturbation analysis.

Donor	Acceptor	E(2) (kcal / mol)
Rh LP	σ* C1 – H11	3.75
σ Rh – P1	σ* C1 – H11	3.77
σ Rh – P2	σ* C1 – H11	1.13
σ C1 – H11	σ* Rh – P2	19.52
Rh LP	σ* C2 – H21	4.13
σ Rh – P1	σ* C2 – H21	0.95
σ Rh – P2	σ* C2 – H21	3.58
σ C2 – H21	σ* Rh – P1	19.78

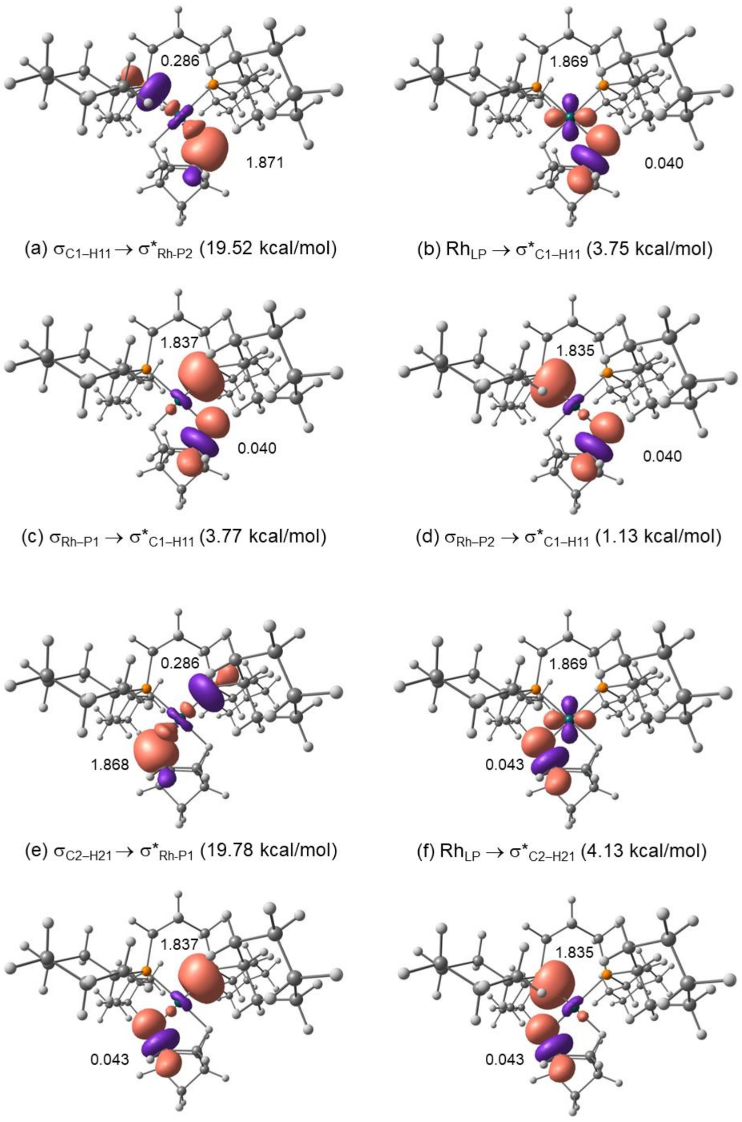

(a) $\sigma_{C1–H11} \rightarrow \sigma^*_{Rh\text{-}P2}$ (19.52 kcal/mol)

(b) $Rh_{LP} \rightarrow \sigma^*_{C1–H11}$ (3.75 kcal/mol)

(c) $\sigma_{Rh–P1} \rightarrow \sigma^*_{C1–H11}$ (3.77 kcal/mol)

(d) $\sigma_{Rh–P2} \rightarrow \sigma^*_{C1–H11}$ (1.13 kcal/mol)

(e) $\sigma_{C2–H21} \rightarrow \sigma^*_{Rh\text{-}P1}$ (19.78 kcal/mol)

(f) $Rh_{LP} \rightarrow \sigma^*_{C2–H21}$ (4.13 kcal/mol)

(g) $\sigma_{Rh–P1} \rightarrow \sigma^*_{C2–H21}$ (0.95 kcal/mol)

(h) $\sigma_{Rh–P2} \rightarrow \sigma^*_{C2–H21}$ (3.58 kcal/mol)

Figure S44: NBO donor-acceptor pairs for **[Cy-*endo*-NBA][BAr^F$_4$]** with NBO occupancies as indicated.

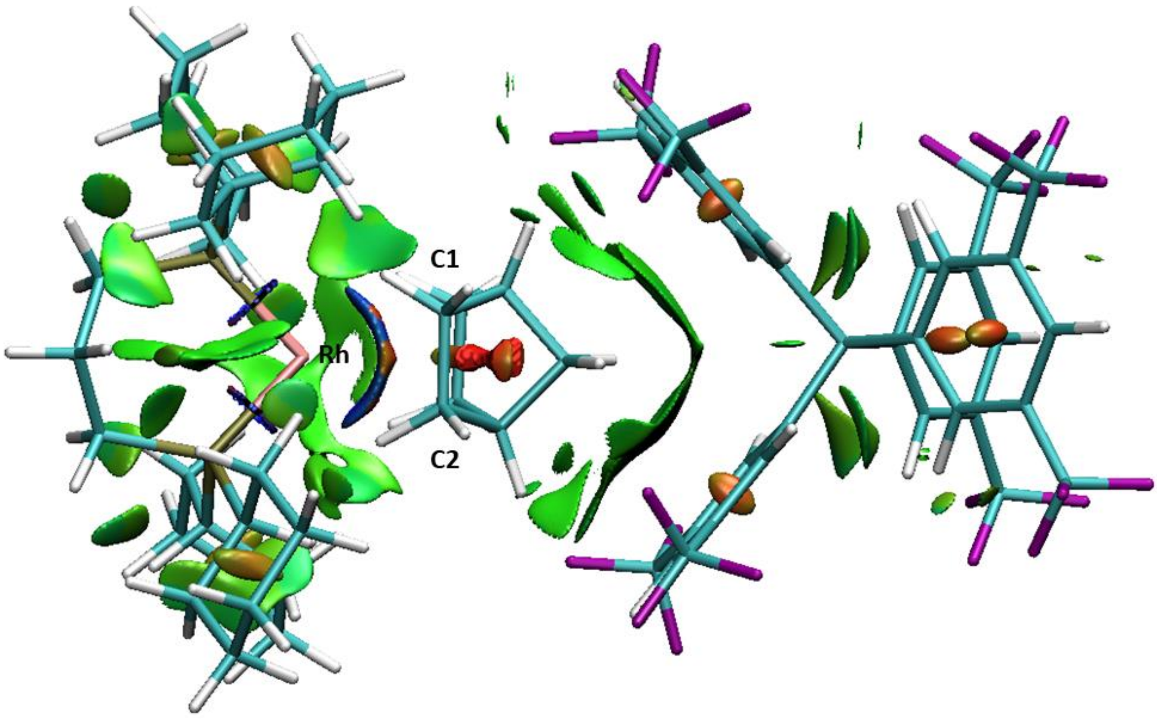

Figure S45: NCI plot of the **[Cy-*endo*-NBA][BAr$^F_4$]** ion-pair. Isosurfaces generated for s = 0.3 au and -0.07 < [illegible] < 0.07 au.

S10.2 [^{t}Bu-*exo*-NBA][BAr$^F_4$]

Table S5: Key bond distances (Å) from fully optimised and heavy atoms fixed geometries of **[^{t}Bu-*exo*-NBA][BAr$^F_4$]**, with experimental data for comparison.

Distance (Å)	Fully Optimised	Heavy Atom Fixed[a]	Experiment
Rh – P1	2.261	2.244	2.2437(18)
Rh – P2	2.254	2.235	2.235(2)
Rh – H11	1.862	1.841	1.772
Rh – H21	1.834	1.821	1.734
Rh – C1	2.420	2.381	2.382(7)
Rh – C2	2.402	2.364	2.363(7)
C1 – H11	1.159	1.162	0.969
C1 – H12	1.095	1.096	0.970
C2 – H21	1.162	1.166	0.971
C2 – H22	1.095	1.096	0.968

[a] structure is based on the experimental structure with only the positions of H and F atoms being optimised.

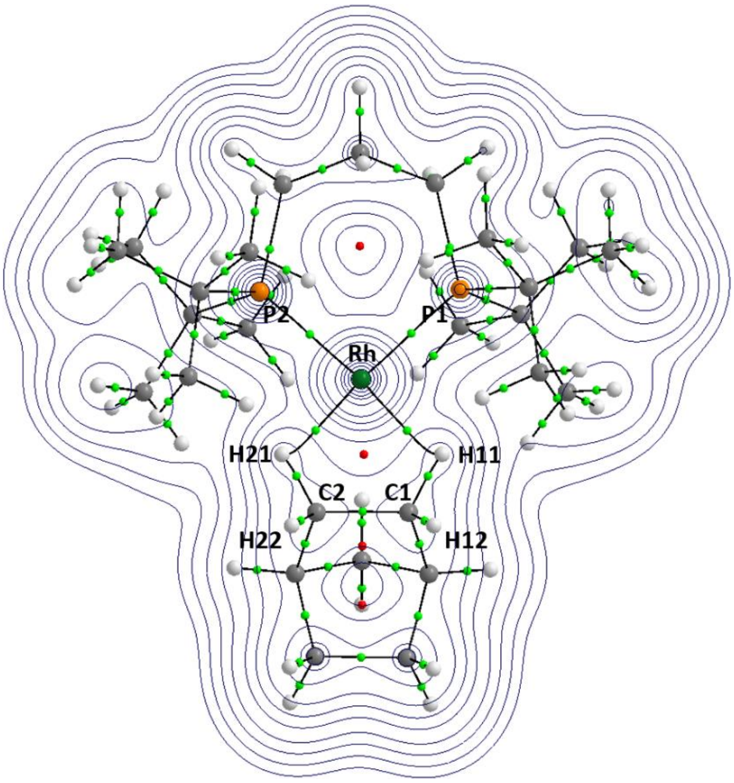

Figure S46: QTAIM molecular graph for heavy atom fixed **[^{t}Bu-*exo*-NBA]$^{+}$** with density contours plotted in the Rh-H11-H21 plane. Bond critical points (BCPs) are indicated in green and ring critical points (RCPs) are indicated in red.

Table S6: Key BCP metrics.

Bond	$\rho\ (r)$ /e bohr^{-3}	$\nabla^2\ \rho\ (r)$ /e bohr^{-5}	H (r)
Rh – P1	0.111	0.098	-0.050
Rh – P2	0.113	0.094	-0.052
Rh – H11	0.066	0.259	-0.011
Rh – H21	0.069	0.266	-0.013
C1 – H11	0.228	-0.579	-0.194
C1 – H12	0.277	-0.953	-0.280
C2 – H21	0.226	-0.564	-0.191
C2– H22	0.278	-0.955	-0.281

Table S7: Key donor-acceptor interactions from the NBO second order perturbation analysis.

Donor	Acceptor	E(2) (kcal / mol)
Rh LP	σ* C1 – H11	4.41
σ Rh – P1	σ* C1 – H11	4.53
σ Rh – P2	σ* C1 – H11	1.00
σ C1 – H11	σ* Rh – P2	22.14
Rh LP	σ* C2 – H21	5.10
σ Rh – P1	σ* C2 – H21	0.96
σ Rh – P2	σ* C2 – H21	4.82
σ C2 – H21	σ* Rh – P1	23.57

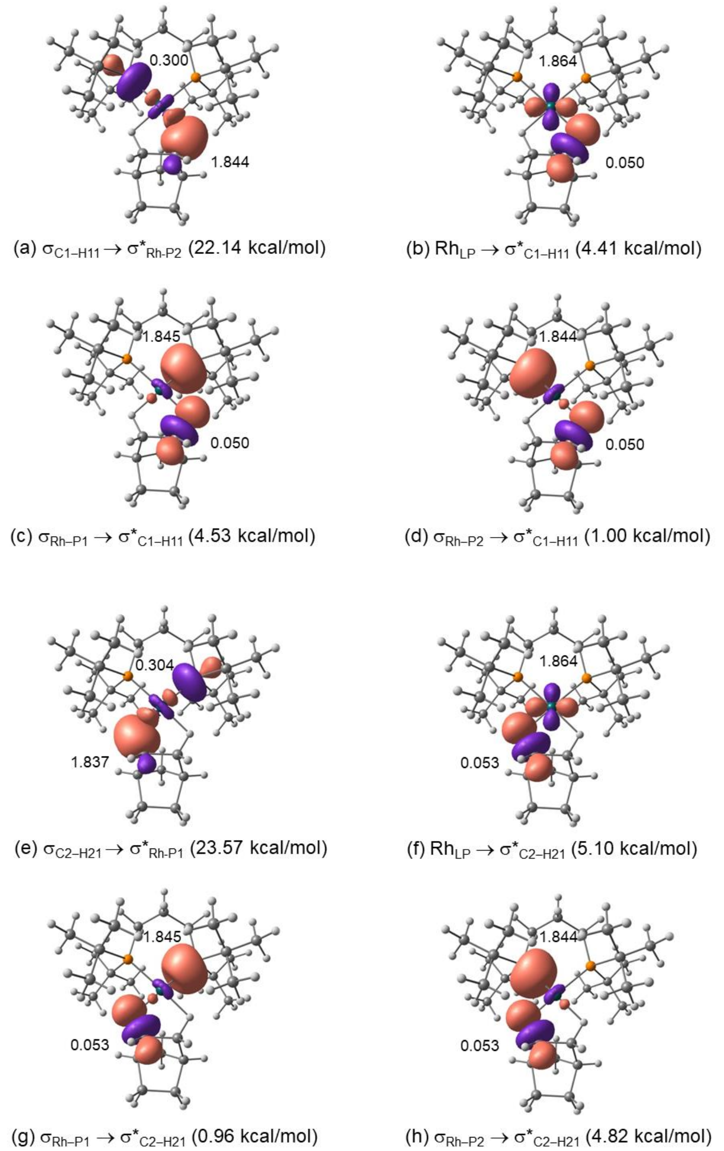

(a) $\sigma_{C1–H11} \rightarrow \sigma^*_{Rh-P2}$ (22.14 kcal/mol)

(b) $Rh_{LP} \rightarrow \sigma^*_{C1–H11}$ (4.41 kcal/mol)

(c) $\sigma_{Rh–P1} \rightarrow \sigma^*_{C1–H11}$ (4.53 kcal/mol)

(d) $\sigma_{Rh–P2} \rightarrow \sigma^*_{C1–H11}$ (1.00 kcal/mol)

(e) $\sigma_{C2–H21} \rightarrow \sigma^*_{Rh-P1}$ (23.57 kcal/mol)

(f) $Rh_{LP} \rightarrow \sigma^*_{C2–H21}$ (5.10 kcal/mol)

(g) $\sigma_{Rh–P1} \rightarrow \sigma^*_{C2–H21}$ (0.96 kcal/mol)

(h) $\sigma_{Rh–P2} \rightarrow \sigma^*_{C2–H21}$ (4.82 kcal/mol)

Figure S47. NBO donor-acceptor pairs for **[^{t}Bu-*exo*-NBA][BAr$^F{}_4$]** with NBO occupancies as indicated.

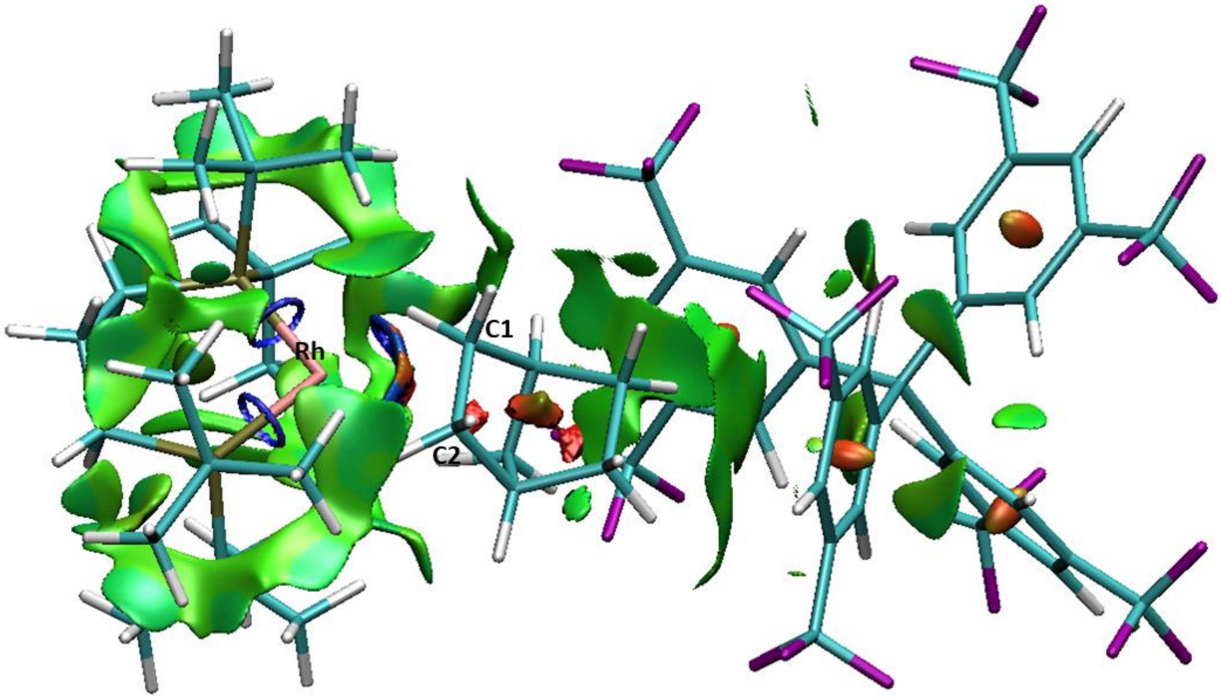

Figure S48: NCI plot of the **[^{t}Bu-*exo*-NBA][BAr$^{F}_{4}$]** ion-pair. Isosurfaces generated for s = 0.3 au and -0.07 < < 0.07 au.

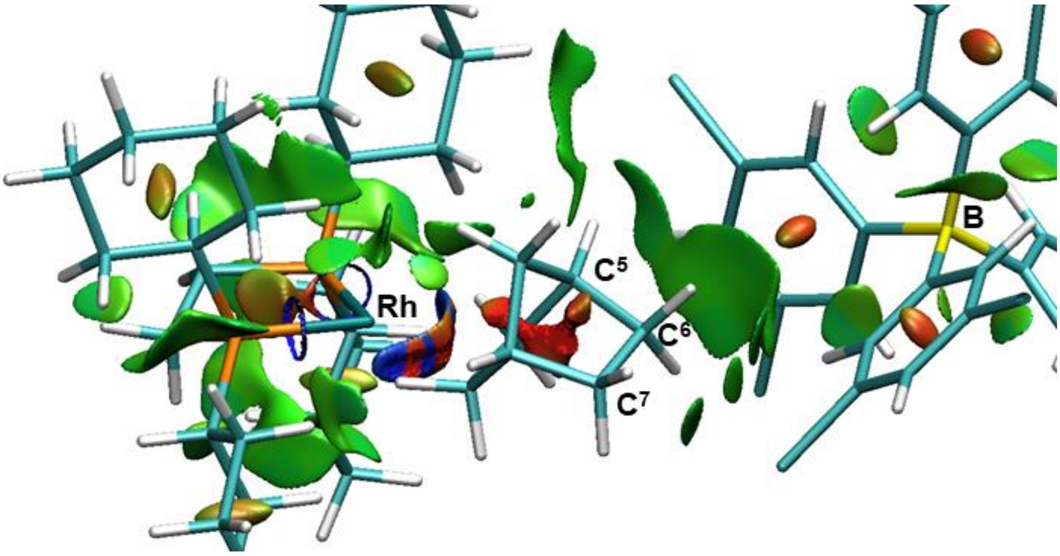

Figure S49: NCI plot of the **[Cy-*exo*-NBA][BAr$^{Cl}_{4}$]** ion-pair. Isosurfaces generated for s = 0.3 au and -0.07 < < 0.07 au.

S11 Isomers of [Rh(dtbpp)(C_4H_8)]$^+$

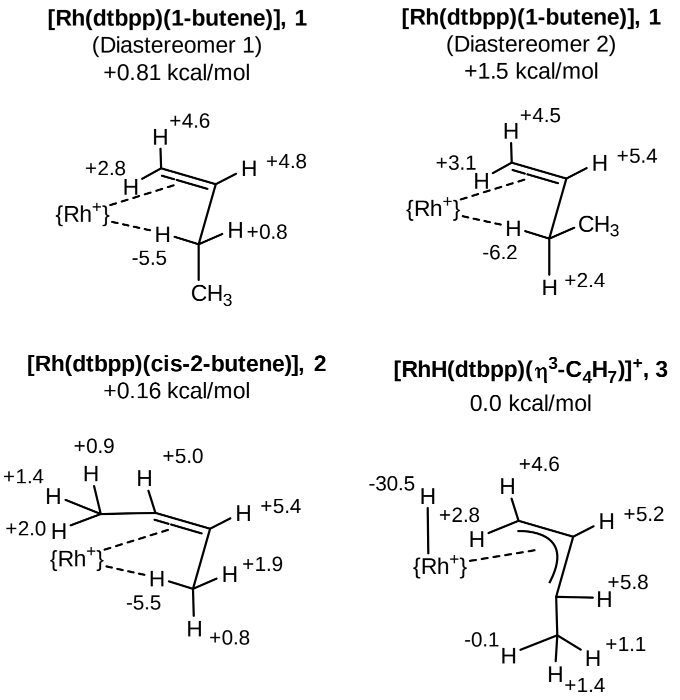

Figure S50: Relative free energies (kcal/mol) of the isomers of **[Rh(dtbpp)(C_4H_8)]$^+$** with computed ^{1}H chemical shifts for the {C_4H_8} hydrogens indicated.

S12 Crystal Explorer

S12.1 [Cy-*endo*/*exo*-NBA][BAr$^F_4$]

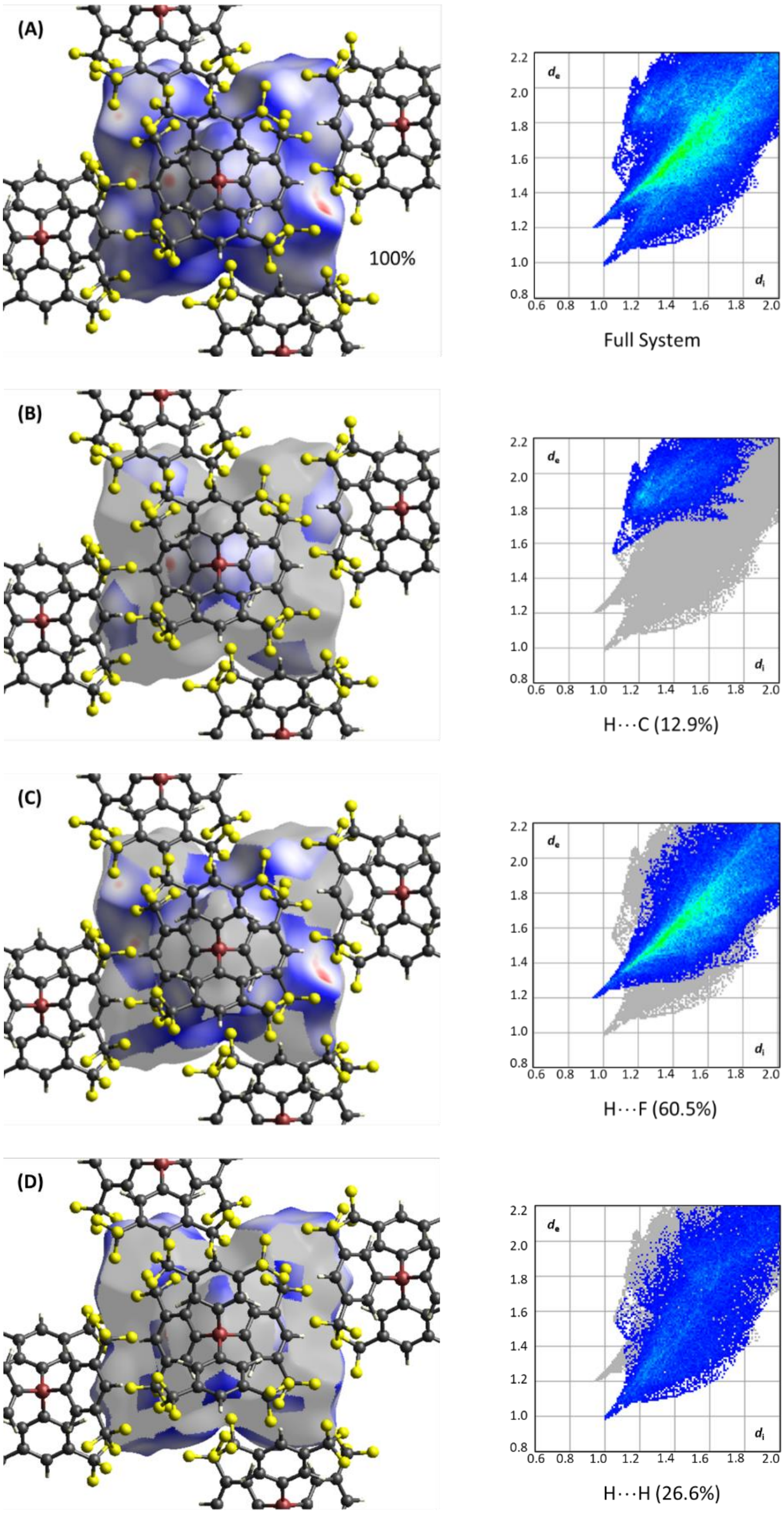

Figure S51: Hirshfeld surfaces mapped over d_{norm} and fingerprint plots (Å) for the **[Cy-*endo*-NBA][BAr$^F_4$]** cation with the surrounding anions. (A) Full system (B) H···C distances (C) H···F distances (D) H···H distances.

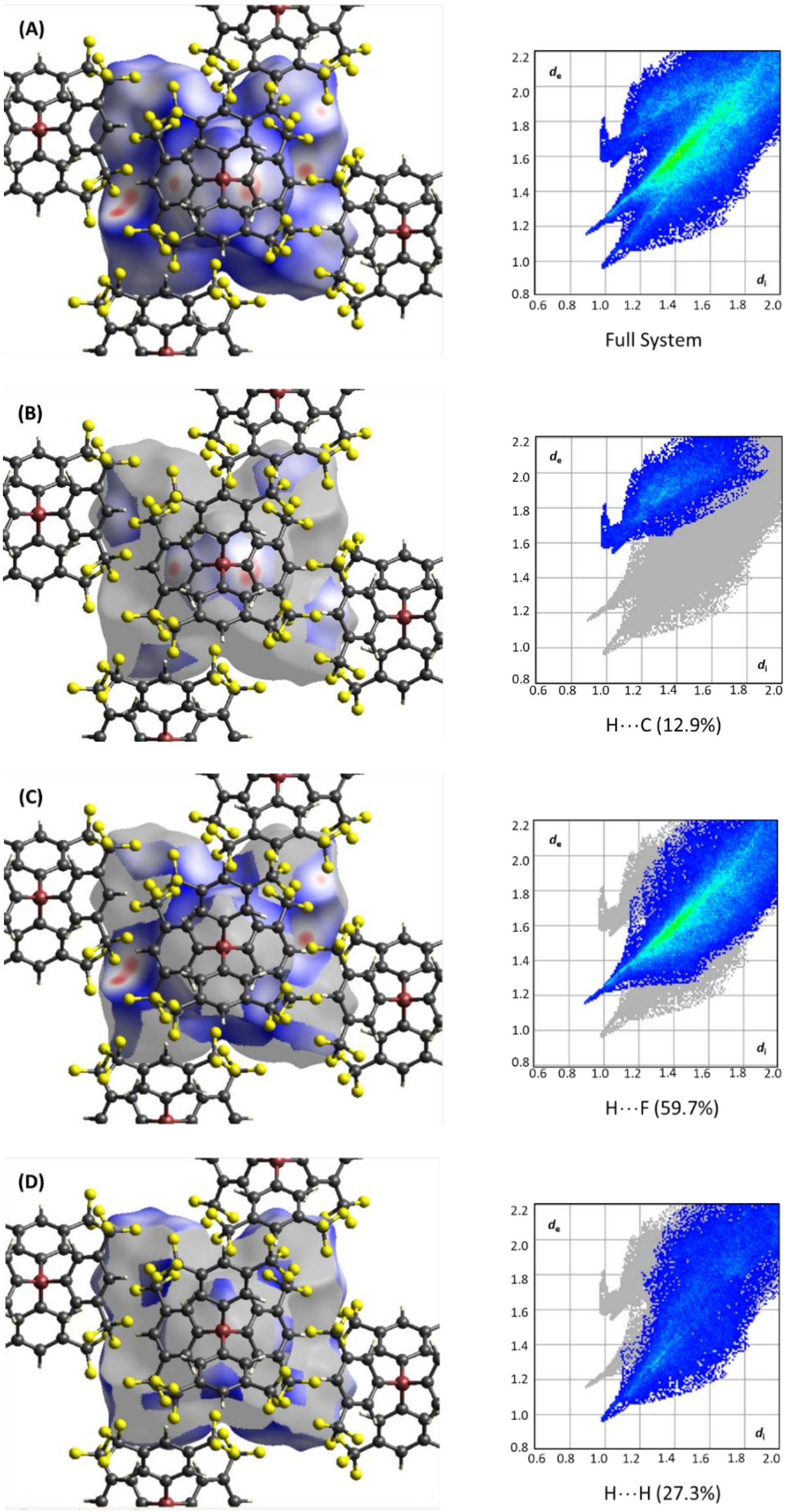

Figure S52: Hirshfeld surfaces mapped over d_{norm} and fingerprint plots (Å) for the **[Cy-*exo*-NBA][BAr$^F{}_4$]** cation with the surrounding anions. (A) Full system (B) H···C distances (C) H···F distances (D) H···H distances.

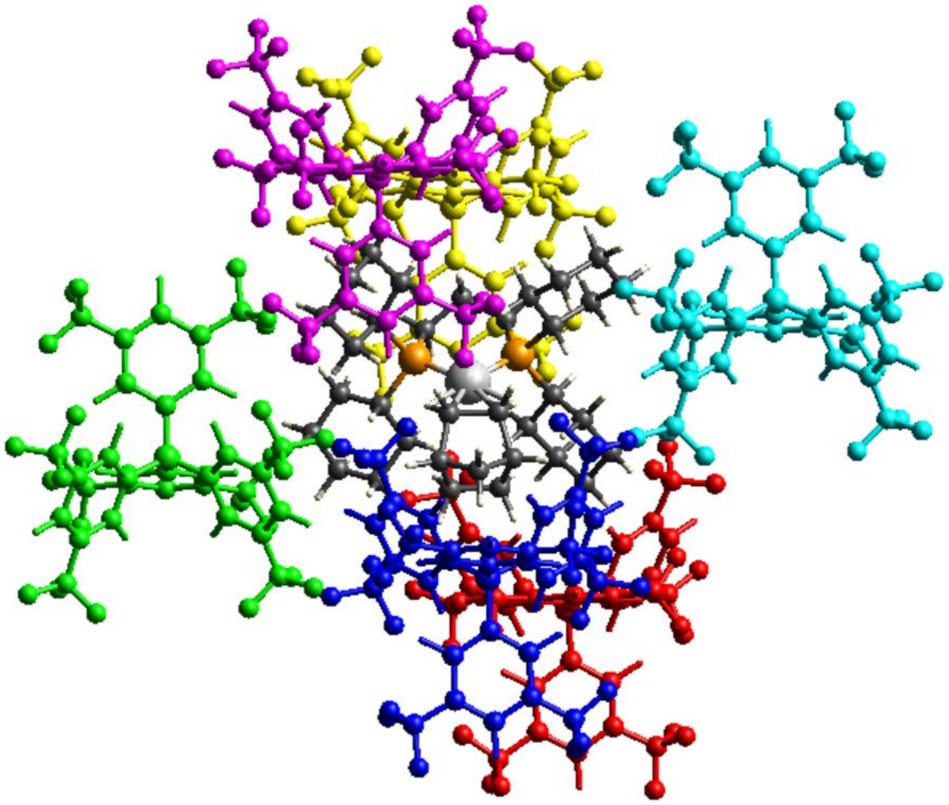

Figure S53: **[Cy-*endo*-NBA][BAr$^F_4$]** cation with the surrounding anions indicated by colour in the table below.

Table S8: Interaction energies for the **[Cy-*endo*-NBA][BAr$^F_4$]** cation with the surrounding anions.

**Anion**	**Distance (Å)**	**E_ele (kJ/mol)**	**E_pol (kJ/mol)**	**E_dis (kJ/mol)**	**E_rep (kJ/mol)**	**E_tot (kJ/mol)**
Bottom (red)	9.72	-136.60	-14.40	-63.70	26.70	-184.30
Back (yellow)	8.35	-158.90	-24.00	-88.20	33.20	-230.00
Left (green)	9.85	-145.10	-15.70	-78.40	33.30	-201.80
Right (cyan)	10.00	-143.20	-15.10	-71.10	42.00	-185.70
Front (blue)	9.46	-144.40	-16.20	-68.50	31.30	-194.00
Top (magenta)	9.39	-145.60	-17.20	-70.50	28.40	-200.10
Average	9.46	-145.63	-17.10	-73.40	32.48	-199.32

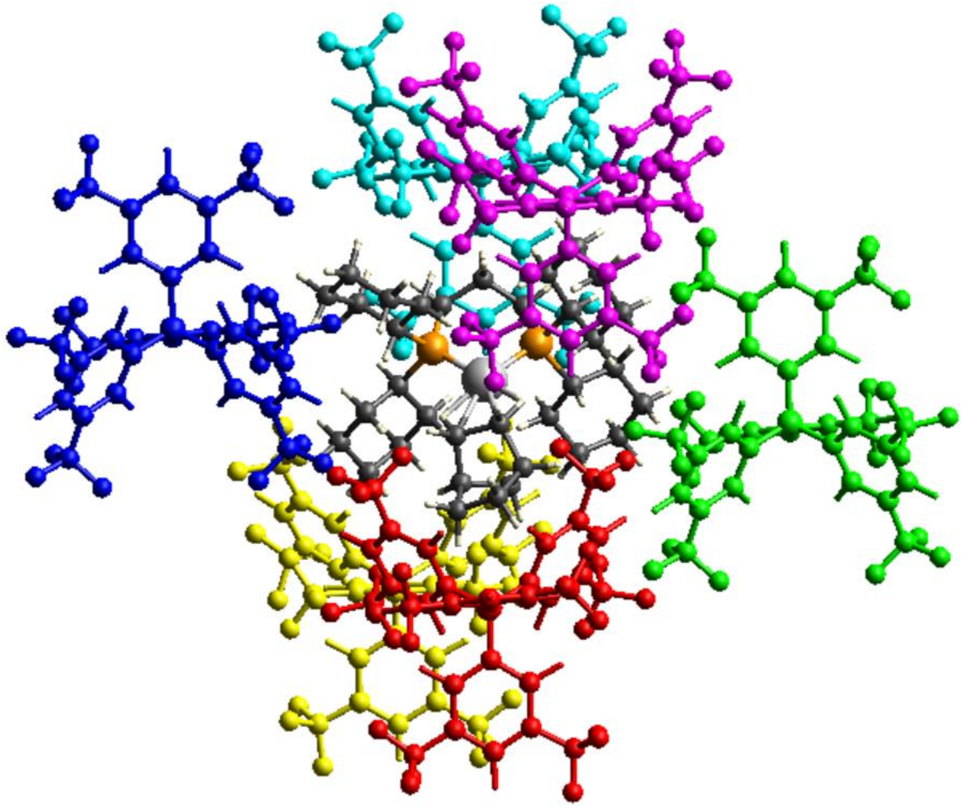

Figure S54: [Cy-*exo*-NBA][BAr^F$_4$] cation with the surrounding anions indicated by colour in the table below.

Table S9: Interaction energies for the **[Cy-*exo*-NBA][BAr^F$_4$]** cation with the surrounding anions.

**Anion**	**Distance (Å)**	**E_ele (kJ/mol)**	**E_pol (kJ/mol)**	**E_dis (kJ/mol)**	**E_rep (kJ/mol)**	**E_tot (kJ/mol)**
Front (red)	9.61	-148.10	-15.00	-68.90	49.20	-182.80
Bottom (yellow)	9.64	-139.20	-15.30	-66.30	30.00	-187.20
Right (green)	9.84	-146.10	-15.80	-79.60	38.40	-199.70
Back (cyan)	8.20	-164.30	-25.60	-100.00	47.80	-235.50
Left (blue)	10.00	-145.70	-15.50	-71.40	47.50	-184.40
Top (magenta)	9.49	-143.50	-16.30	-66.50	27.20	-194.70
Average	9.46	-147.82	-17.25	-75.45	40.02	-197.38

S12.2 [^{t}Bu-*exo*/*endo*-NBA][BAr$^{F}{}_{4}$]

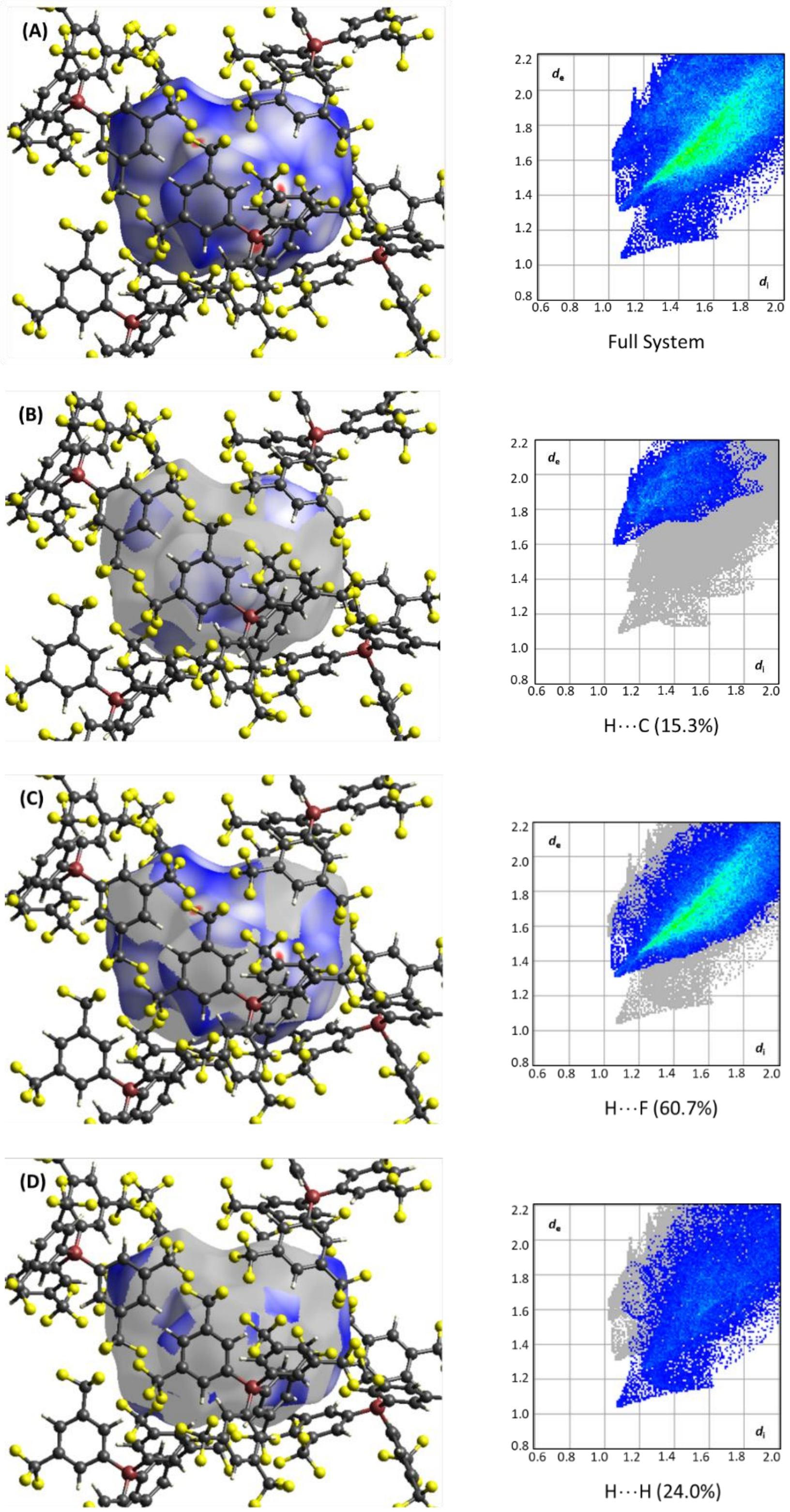

Figure S55: Hirshfeld surfaces mapped over d_{norm} and fingerprint plots (Å) for the **[^{t}Bu-*endo*-NBA][BAr$^{F}{}_{4}$]** cation with the surrounding anions. (A) Full system (B) H···C distances (C) H···F distances (D) H···H distances.

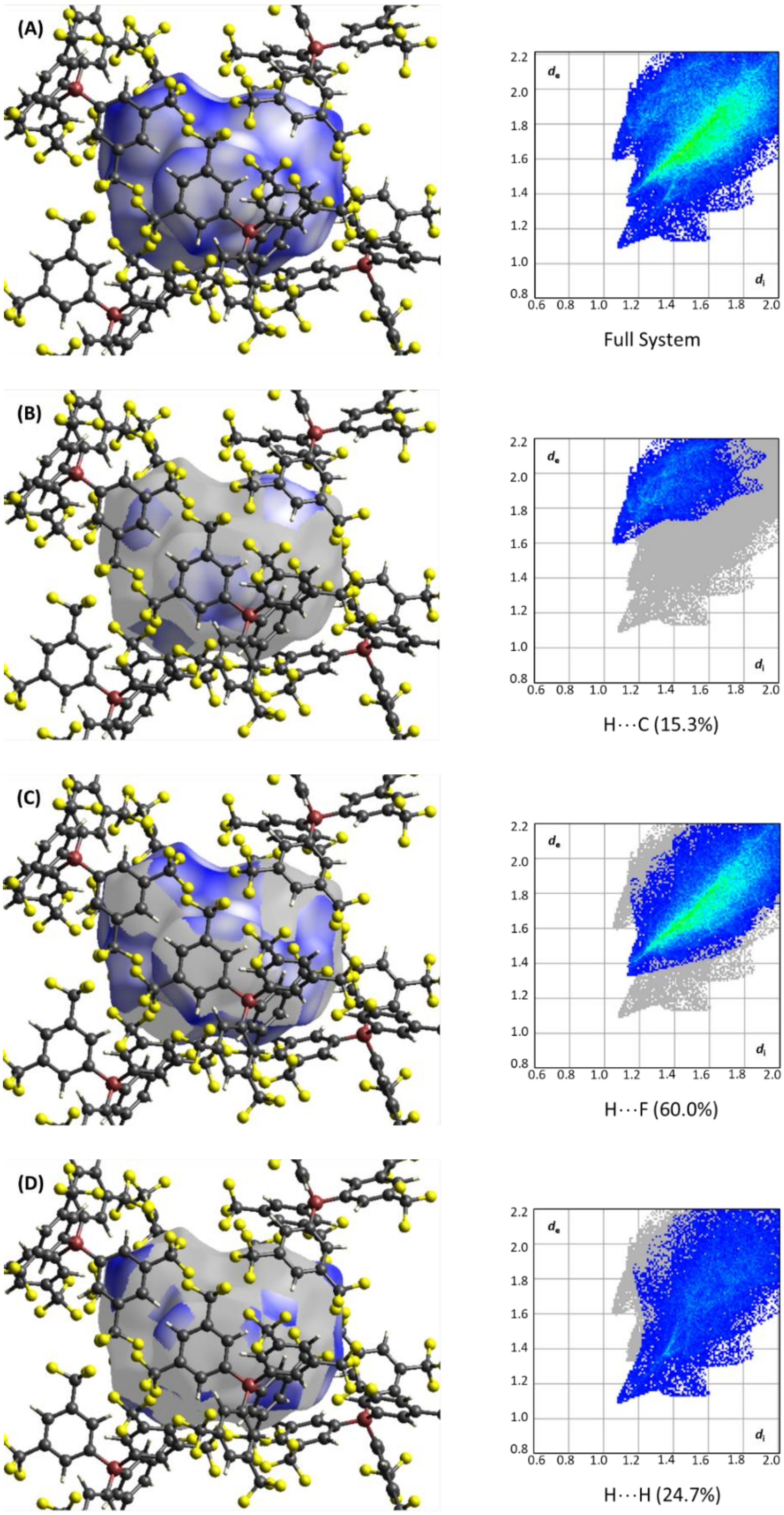

Figure S56: Hirshfeld surfaces mapped over d_{norm} and fingerprint plots (Å) for the **[^{t}Bu-*exo*-NBA][BAr^F$_4$]** cation with the surrounding anions. (A) Full system (B) H···C distances (C) H···F distances (D) H···H distances.

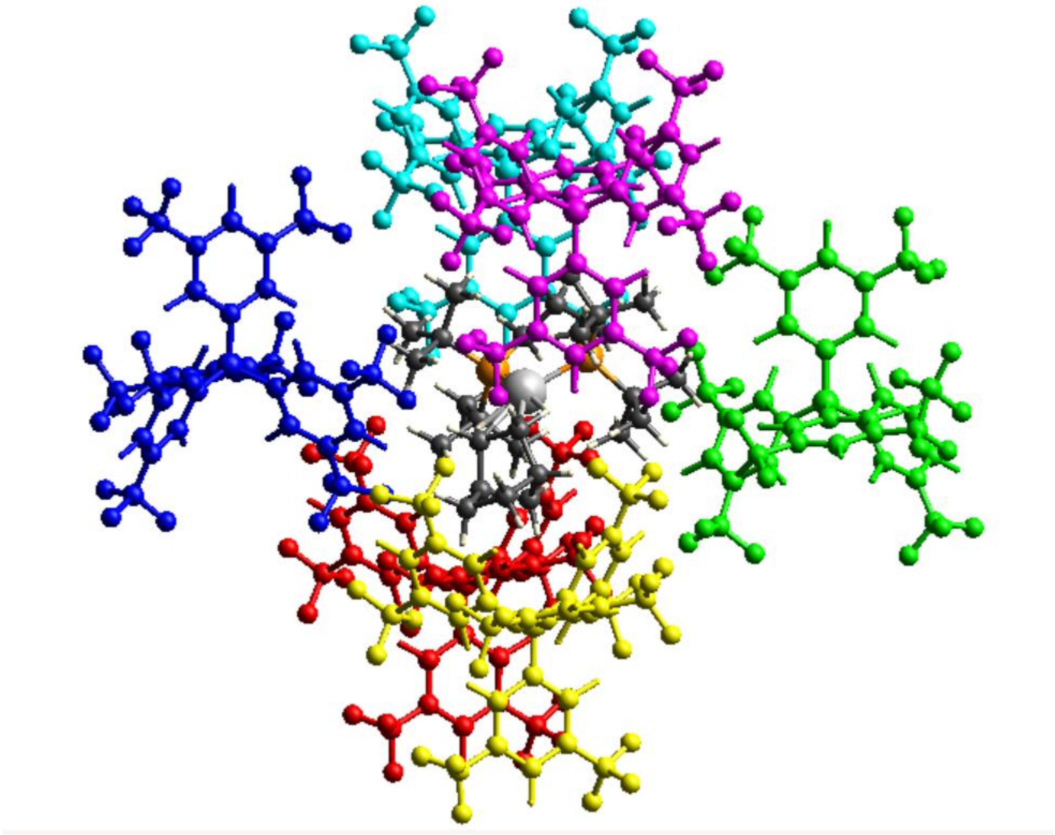

Figure S57: [^{t}Bu-*exo*-NBA][BAr$^{F}{}_{4}$] cation with the surrounding anions indicated by colour in the table below.

Table S10: Interaction energies for the **[^{t}Bu-*exo*-NBA][BAr$^{F}{}_{4}$]** cation with the surrounding anions.

**Anion**	**Distance (Å)**	**E_ele (kJ/mol)**	**E_pol (kJ/mol)**	**E_dis (kJ/mol)**	**E_rep (kJ/mol)**	**E_tot (kJ/mol)**
Bottom (red)	8.92	-144.70	-15.60	-63.80	17.60	-200.80
Front (yellow)	9.72	-141.10	-14.90	-64.60	27.50	-189.30
Right (green)	9.30	-143.80	-14.60	-58.20	20.90	-191.60
Back (cyan)	9.17	-147.70	-16.90	-57.60	15.00	-201.20
Left (blue)	9.40	-141.90	-14.60	-50.00	14.90	-187.00
Top (magenta)	8.71	-153.80	-18.60	-63.50	24.90	-205.80
Average	9.20	-145.50	-15.87	-59.62	20.13	-195.95

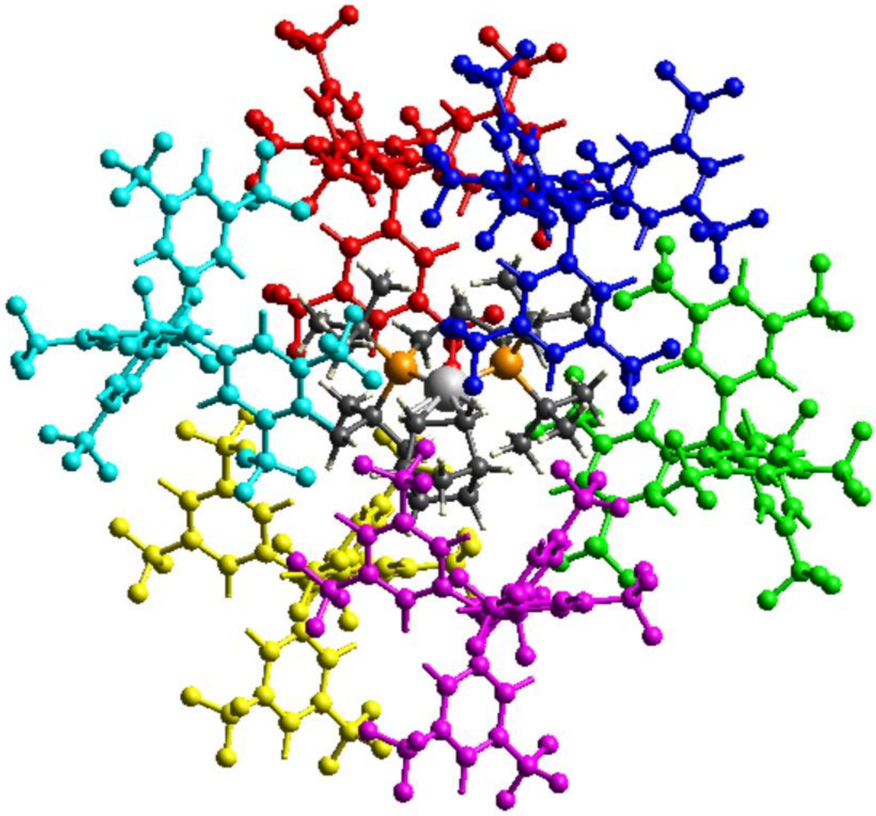

Figure S58: **[^{t}Bu-*endo*-NBA][BAr^F$_4$]** cation with the surrounding anions indicated by colour in the table below.

Table S11: Interaction energies for the **[^{t}Bu-*endo*-NBA][BAr^F$_4$]** cation with the surrounding anions.

**Anion**	**Distance (Å)**	**E_ele (kJ/mol)**	**E_pol (kJ/mol)**	**E_dis (kJ/mol)**	**E_rep (kJ/mol)**	**E_tot (kJ/mol)**
Back (red)	9.13	-147.60	-17.10	-56.70	13.30	-201.80
Bottom (yellow)	8.94	-145.70	-15.20	-65.60	22.40	-199.30
Right (green)	9.31	-144.20	-14.80	-59.10	21.90	-192.10
Left (cyan)	9.39	-143.70	-14.60	-53.20	20.50	-187.20
Top (blue)	8.71	-154.60	-19.10	-63.70	26.20	-206.10
Front (magenta)	9.73	-139.70	-15.10	-57.30	27.60	-181.50
Average	9.20	-145.92	-15.98	-59.27	21.98	-194.67

S13 Computed Energies for Molecular Species

Table S12. Computed Energies for the isomers of [RhH(dtbpp)(C_4H_8)]$^+$.

Species	SCF	G (298 K)	SCF(corr)[a]	G (298 K)(corr) [a]	Relative Energy /kcal/mol
[RhH(dtbpp)(η^3-C_4H_7)]$^+$, 3	-1028.85482	-1028.24431	-1698.597688	-1697.987181	0.0
[Rh(dtbpp)(cis-2-butene)], 2	-1028.85287	-1028.24334	-1698.596459	-1697.986934	+0.16
[Rh(dtbpp)(1-butene)], 1 (Diastereomer 1)	-1028.85290	-1028.24282	-1698.595968	-1697.985886	+0.81
[Rh(dtbpp)(1-butene)], 1 (Diastereomer 2)	-1028.85162	-1028.241644	-1698.594851	-1697.984872	+1.45

[a] includes corrections for dispersion (D3) solvent (dichloromethane) computed with a def2-TZVP basis set.

Table S13. Computed Energies for the [Cy-NBA]$^+$ and [^{t}Bu-NBA]$^+$ Molecular Cations.

Species	SCF	G (298 K)	SCF(corr)[a]	G (298 K)(corr) [a]	Relative Energy /kcal/mol
[Cy-*endo*-NBA]$^+$	-1454.755394	-1453.942287	-2124.59715	-2123.784043	+1.52
[Cy-*exo*-NBA]$^+$	-1454.757471	-1453.94631	-2124.597628	-2123.786467	0.00
[^{t}Bu-*endo*-NBA]$^+$	-1145.426442	-1144.75234	-1815.164671	-1814.490569	+0.95
[^{t}Bu-*exo*-NBA]$^+$	-1145.428491	-1144.754819	-1815.165754	-1814.492082	0.00

[a] includes a correction for dispersion (D3) computed with a def2-TZVP basis set.

S14 References

[1] T. R. Hoye, B. M. Eklov, M. Voloshin, *Org. Lett.* **2004**, *6*, 2567-2570.

[2] G. Giordano, R. H. Crabtree, in *Inorganic Syntheses*, pp. 88-90.

[3] A. J. Martínez-Martínez, A. S. Weller, *Dalton Trans.* **2019**, *48*, 3551-3554.

[4] aJ. Huber, S. Mecking, *Macromolecules* **2010**, *43*, 8718-8723; bB. Guzel, M. A. Omary, J. P. Fackler, A. Akgerman, *Inorg. Chim. Acta* **2001**, *325*, 45-50.

[5] G. R. Fulmer, A. J. M. Miller, N. H. Sherden, H. E. Gottlieb, A. Nudelman, B. M. Stoltz, J. E. Bercaw, K. I. Goldberg, *Organometallics* **2010**, *29*, 2176-2179.

[6] O. B. Peersen, X. L. Wu, I. Kustanovich, S. O. Smith, *J. Magn. Res., Series A* **1993**, *104*, 334-339.

[7] W. L. Earl, D. L. Vanderhart, *J. Magn. Res.* **1982**, *48*, 35-54.

[8] J. Cosier, A. M. Glazer, *J. Appl. Crystallogr.* **1986**, *19*, 105-107.

[9] J. VandeVondele, J. Hutter, *J. Chem. Phys.* **2007**, *127*, 114105.

[10] G. Sheldrick, *Acta Cryst. A* **2015**, *71*, 3-8.

[11] G. Sheldrick, *Acta Cryst. A* **2008**, *64*, 112-122.

[12] O. V. Dolomanov, L. J. Bourhis, R. J. Gildea, J. A. K. Howard, H. Puschmann, *J. Appl. Crystallogr.* **2009**, *42*, 339-341.

[13] A. T. Lubben, J. S. McIndoe, A. S. Weller, *Organometallics* **2008**, *27*, 3303-3306.

[14] N. Carr, B. J. Dunne, L. Mole, A. G. Orpen, J. L. Spencer, *J. Chem. Soc., Dalton Trans.* **1991**, 863-871.

[15] B. Nguyen, J. M. Brown, *Adv. Synth. Catal* **2009**, *351*, 1333-1343.

[16] L. Falivene, Z. Cao, A. Petta, L. Serra, A. Poater, R. Oliva, V. Scarano, L. Cavallo, *Nat. Chem.* **2019**, *11*, 872-879.

[17] S. Alvarez, *Dalton Trans.* **2013**, *42*, 8617-8636.

[18] J. VandeVondele, M. Krack, F. Mohamed, M. Parrinello, T. Chassaing and J. Hutter, *Comput. Phys. Commun.* **2005**, *167*, 103-128.

[19] J. Hutter, M. Iannuzzi, F. Schiffmann and J. VandeVondele, *Wiley Interdiscip. Rev. Comput. Mol. Sci.* **2013**, *4*, 15-25.

[20] J. VandeVondele and J. Hutter, *J. Chem. Phys.* **2007**, *127*, 114105.

[21] C. Hartwigsen, S. Goedecker and J. Hutter, *Phys. Rev. B* **1998**, *58*, 3641-3662.

[22] S. Goedecker, M. Teter and J. Hutter, *Phys. Rev. B* **1996**, *54*, 1703-1710.

[23] M. Krack, *Theor. Chem. Acc.* **2005**, *114*, 145-152.

[24] J. P. Perdew, K. Burke and M. Ernzerhof, *Phys. Rev. Lett.* **1996**, *77*, 3865-3868.

[25] S. Grimme, J. Antony, S. Ehrlich and H. Krieg, *J. Chem. Phys.* **2010**, *132*, 154104.

[26] G. Henkelman and H. Jónsson, *J. Chem. Phys.* **1999**, *111*, 7010-7022.

[27] G. Henkelman, B. P. Uberuaga and H. Jónsson, *J. Chem. Phys.* **2000**, *113*, 9901-9904.

[28] F. M. Chadwick, T. Krämer, T. Gutmann, N. H. Rees, A. L. Thompson, A. J. Edwards, G. Buntkowsky, S. A. Macgregor and A. S. Weller, *J. Am. Chem. Soc.* **2016**, *138*, 13369-13378.

[29] R. Y. Brogaard, B. M. Weckhuysen and J. K. Nørskov, *J. Catal.* **2013**, *300*, 235-241.

[30] G. Piccini and J. Sauer, *J. Chem. Theor. Comput.* **2013**, *9*, 5038-5045.

[31] A. Ghysels, T. Verstraelen, K. Hemelsoet, M. Waroquier and V. Van Speybroeck, *J. Chem. Inf. Model.* **2010**, *50*, 1736-1750.

[32] M. J. Frisch, G. W. Trucks, H. B. Schlegel, G. E. Scuseria, M. A. Robb, J. R. Cheeseman, G. Scalmani, V. Barone, B. Mennucci, G. A. Petersson, H. Nakatsuji, M. Caricato, X. Li, H. P. Hratchian, A. F. Izmaylov, J. Bloino, G. Zheng, J. L. Sonnenberg, M. Hada, M. Ehara, K. Toyota, R. Fukuda, J. Hasegawa, M. Ishida, T. Nakajima, Y. Honda, O. Kitao, H. Nakai, T. Vreven, J. A. Montgomery, J. E. Peralta, F. Ogliaro, M. Bearpark, J. J. Heyd, E. Brothers, K. N. Kudin, V. N. Staroverov, R. Kobayashi, J. Normand, K. Raghavachari, A. Rendell, J. C. Burant, S. S. Iyengar, J. Tomasi, M. Cossi, N. Rega, J. M. Millam, M. Klene, J. E. Knox, J. B. Cross, V. Bakken, C. Adamo, J. Jaramillo, R. Gomperts, R. E. Stratmann, O. Yazyev, A. J. Austin, R. Cammi, C. Pomelli, J. W. Ochterski, R. L. Martin, K. Morokuma, V. G. Zakrzewski, G. A. Voth, P. Salvador, J. J. Dannenberg, S. Dapprich, A. D. Daniels, Farkas, J. B. Foresman, J. V. Ortiz, J. Cioslowski and D. J. Fox in *Gaussian 09, Revision D.01*, Gaussian Inc., Wallingford CT, **2013**.

[33] A. D. Becke, *Phys. Rev. A* **1988**, *38*, 3098-3100.

[34] J. P. Perdew, *Phys. Rev. B* **1986**, *33*, 8822-8824.

[35] D. Andrae, U. Häußermann, M. Dolg, H. Stoll and H. Preuß, *Theor. Chim. Acta* **1990**, *77*, 123-141.

[36] A. Höllwarth, M. Böhme, S. Dapprich, A. W. Ehlers, A. Gobbi, V. Jonas, K. F. Köhler, R. Stegmann, A. Veldkamp and G. Frenking, *Chem. Phys. Lett.* **1993**, *208*, 237-240.

[37] W. J. Hehre, R. Ditchfield and J. A. Pople, *J. Chem. Phys.* **1972**, *56*, 2257-2261.

[38] P. C. Hariharan and J. A. Pople, *Theor. Chim. Acta* **1973**, *28*, 213-222.

[39] R. F. W. Bader, *Atoms in Molecules: A Quantum Theory*, Clarendon Press, **1994**.

[40] T. A. Keith in *AIMAll (Version 17.11.14)*, TK Gristmill Software, Overland Park KS, USA, **2017**.

[41] E. D. Glendening, C. R. Landis and F. Weinhold, *J. Comput. Chem.* **2013**, *34*, 1429-1437.

[42] E. R. Johnson, S. Keinan, P. Mori-Sánchez, J. Contreras-García, A. J. Cohen and W. Yang, *J. Am. Chem. Soc.* **2010**, *132*, 6498-6506.

[43] J. Contreras-García, E. R. Johnson, S. Keinan, R. Chaudret, J.-P. Piquemal, D. N. Beratan and W. Yang, *J. Chem. Theory Comput.* **2011**, *7*, 625-632.

[44] Chemcraft - graphical software for visualization of quantum chemistry computations. https://www.chemcraftprog.com

[45] C. F. Macrae, I. J. Bruno, J. A. Chisholm, P. R. Edgington, P. McCabe, E. Pidcock, L. Rodriguez-Monge, R. Taylor, J. van de Streek and P. A. Wood, *J. Appl. Cryst.* **2008**, *41*, 466-470.

[46] K. Momma and F. Izumi, *J. Appl. Cryst.* **2011**, *44*, 1272-1276.

[47] M. J. Turner, J. J. McKinnon, S. K. Wolff, D. J. Grimwood, P. R. Spackman, D. Jayatilaka and M. A. Spackman, **2017**, University of Western Australia. https://hirshfeldsurface.net.

[48] F. Weigend and R. Ahlrichs, *Phys. Chem. Chem. Phys.* **2005**, *7*, 3297-3305.

[49] F. Weigend, *Phys. Chem. Chem. Phys.* **2006**, *8*, 1057-1065.

[50] J. Tomasi, B. Mennucci and R. Cammi, *Chem. Rev.* **2005**, *105*, 2999-3093.

[51] L. J. L. Häller, M. J. Page, S. Erhardt, S. A. Macgregor, M. F. Mahon, M. A. Naser, A. Vélez and M. K. Whittlesey, *J. Am. Chem. Soc.* **2010**, *132*, 18408-18416.

[52] W. Humphrey, A. Dalke and K. Schulten, *J Mol. Graph.* **1996**, *14*, 33-38.

[53] G. te Velde, F. M. Bickelhaupt, E. J. Baerends, C. Fonseca Guerra, S. J. A. van Gisbergen, J. G. Snijders and T. Ziegler, *J. Comput. Chem.* **2001**, *22*, 931-967.

[54] E.J. Baerends, T. Ziegler, A.J. Atkins, J. Autschbach, O. Baseggio, D. Bashford, A. Bérces, F.M. Bickelhaupt, C. Bo, P.M. Boerrigter, L. Cavallo, C. Daul, D.P. Chong, D.V. Chulhai, L. Deng, R.M. Dickson, J.M. Dieterich, D.E. Ellis, M. van Faassen, L. Fan, T.H. Fischer, A. Förster, C. Fonseca Guerra, M. Franchini, A. Ghysels, A. Giammona, S.J.A. van Gisbergen, A. Goez, A.W. Götz, J.A. Groeneveld, O.V. Gritsenko, M. Grüning, S. Gusarov, F.E. Harris, P. van den Hoek, Z. Hu, C.R. Jacob, H. Jacobsen, L. Jensen, L. Joubert, J.W. Kaminski, G. van Kessel, C. König, F. Kootstra, A. Kovalenko, M.V. Krykunov, E. van Lenthe, D.A. McCormack, A. Michalak, M. Mitoraj, S.M. Morton, J. Neugebauer, V.P. Nicu, L. Noodleman, V.P. Osinga, S. Patchkovskii, M. Pavanello, C.A. Peeples, P.H.T. Philipsen, D. Post, C.C. Pye, H. Ramanantoanina, P. Ramos, W. Ravenek, J.I. Rodríguez, P. Ros, R. Rüger, P.R.T. Schipper, D. Schlüns, H. van Schoot, G. Schreckenbach, J.S. Seldenthuis, M. Seth, J.G. Snijders, M. Solà, M. Stener, M. Swart, D. Swerhone, V. Tognetti, G. te Velde, P. Vernooijs, L. Versluis, L. Visscher, O. Visser, F. Wang, T.A. Wesolowski, E.M. van Wezenbeek, G. Wiesenekker, S.K. Wolff, T.K. Woo and A.L. Yakovlev in *ADF 2019.3*, SCM, Theoretical Chemistry, Vrije Universiteit, Amsterdam, The Netherlands, http://www.scm.com.

[55] C. Adamo and V. Barone, *J. Chem. Phys*. **1999**, *110*, 6158-6170.

[56] E. van Lenthe and E. J. Baerends, *J. Comput. Chem.* **2003**, *24*, 1142-1156.

[57] G. Schreckenbach and T. Ziegler, *J. Phys. Chem.* **1995**, *99*, 606-611.

[58] M. Krykunov, T. Ziegler and E. van Lenthe, *Int. J. Quant. Chem.* **2009**, *109*, 1676-1683.
